# International Union of Basic and Clinical Pharmacology. CIX. Differences and Similarities between Human and Rodent Prostaglandin E_2_ Receptors (EP1–4) and Prostacyclin Receptor (IP): Specific Roles in Pathophysiologic Conditions

**DOI:** 10.1124/pr.120.019331

**Published:** 2020-10

**Authors:** Xavier Norel, Yukihiko Sugimoto, Gulsev Ozen, Heba Abdelazeem, Yasmine Amgoud, Amel Bouhadoun, Wesam Bassiouni, Marie Goepp, Salma Mani, Hasanga D. Manikpurage, Amira Senbel, Dan Longrois, Akos Heinemann, Chengcan Yao, Lucie H. Clapp

**Affiliations:** Université de Paris, Institut National de la Sante et de la Recherche Medicale (INSERM), UMR-S 1148, CHU X. Bichat, Paris, France (X.N., G.O., H.A., Y.A., A.B., S.M., H.D.M., A.S., D.L.); Université Sorbonne Paris Nord, Villetaneuse, France (X.N., H.A., Y.A., A.B., S.M., D.L.); Department of Pharmaceutical Biochemistry, Graduate School of Pharmaceutical Sciences, Kumamoto University, Chuo-ku, Kumamoto, Japan (Y.S.); Istanbul University, Faculty of Pharmacy, Department of Pharmacology, Istanbul, Turkey (G.O.); Department of Pharmacology and Toxicology, Faculty of Pharmacy, Alexandria University, Alexandria, Egypt (A.S., H.A., W.B.); Centre for Inflammation Research, Queen’s Medical Research Institute, The University of Edinburgh, Edinburgh, United Kingdom (C.Y., M.G.); Institut Supérieur de Biotechnologie de Monastir (ISBM), Université de Monastir, Monastir, Tunisia (S.M.); CHU X. Bichat, AP-HP, Paris, France (D.L.); Otto Loewi Research Center for Vascular Biology, Immunology and Inflammation, Division of Pharmacology, Medical University of Graz, Graz, Austria (A.H.); and Centre for Cardiovascular Physiology & Pharmacology, University College London, London, United Kingdom (L.H.C.)

## Abstract

Prostaglandins are derived from arachidonic acid metabolism through cyclooxygenase activities. Among prostaglandins (PGs), prostacyclin (PGI_2_) and PGE_2_ are strongly involved in the regulation of homeostasis and main physiologic functions. In addition, the synthesis of these two prostaglandins is significantly increased during inflammation. PGI_2_ and PGE_2_ exert their biologic actions by binding to their respective receptors, namely prostacyclin receptor (IP) and prostaglandin E_2_ receptor (EP) 1–4, which belong to the family of G-protein–coupled receptors. IP and EP1–4 receptors are widely distributed in the body and thus play various physiologic and pathophysiologic roles. In this review, we discuss the recent advances in studies using pharmacological approaches, genetically modified animals, and genome-wide association studies regarding the roles of IP and EP1–4 receptors in the immune, cardiovascular, nervous, gastrointestinal, respiratory, genitourinary, and musculoskeletal systems. In particular, we highlight similarities and differences between human and rodents in terms of the specific roles of IP and EP1–4 receptors and their downstream signaling pathways, functions, and activities for each biologic system. We also highlight the potential novel therapeutic benefit of targeting IP and EP1–4 receptors in several diseases based on the scientific advances, animal models, and human studies.

**SIGNIFICANCE STATEMENT:**

In this review, we present an update of the pathophysiologic role of the prostacyclin receptor, prostaglandin E_2_ receptor (EP) 1, EP2, EP3, and EP4 receptors when activated by the two main prostaglandins, namely prostacyclin and prostaglandin E_2_, produced during inflammatory conditions in human and rodents. In addition, this comparison of the published results in each tissue and/or pathology should facilitate the choice of the most appropriate model for the future studies.

## I. Introduction

In comparison with other prostanoids, prostaglandin (PG) E_2_ and prostacyclin (PGI_2_) are dramatically increased during inflammatory processes and pathologic conditions in different organs. Both mediators are synthesized from the same precursors. The process starts by the action of the enzyme cytosolic phospholipase A_2_ on plasma membrane phospholipids, which results in the production of arachidonic acid (AA). AA is then transformed by cyclooxygenase (COX)-1 and COX-2 into the unstable metabolite PGH_2_. Synthesis of the final PG product depends on the catalytic activity of the enzyme acting on PGH_2_. PGE_2_ is synthesized via the isomerization of PGH_2_ by PGE_2_ synthases, whereas PGI_2_ is produced by another isomerase, namely PGI_2_ synthase (PGIS) (Wu and Liou, 2005; Norberg et al., 2013). It is important to note that the rate-limiting step in this pathway is the conversion of AA to PGH_2_ by COX-1/2 (Cathcart et al., 2010).

COX-1 is constitutively expressed in most tissues and is responsible for the production of the majority of prostanoids that are involved in the homeostasis of normal physiologic processes, such as, for instance, gastric wall protection (Yang and Chen, 2016). COX-2, however, is both constitutively expressed in various human tissues (e.g., kidney and brain) and can be induced in numerous cells (including macrophages, vascular smooth muscle, endothelial cells) during inflammation and cancer (Patrono, 2016). Three different isoforms of PGES exist: cytosolic PGES and two microsomal isoforms, microsomal PGES (mPGES)-1 and mPGES-2. Both cytosolic PGES and mPGES-2 are constitutively expressed, whereas mPGES-1 is induced by inflammatory mediators along with COX-2 (Ricciotti and FitzGerald, 2011). Gene deletion of mPGES-1 will lead to a sustained reduction in cellular PGE_2_, showing the importance of this isoform in regulating PGE_2_ synthesis, but will also lead to a shift toward the biosynthesis of PGI_2_ (Ricciotti and FitzGerald, 2011). PGIS is constitutively expressed in several tissues, although it can also be induced during inflammation (Wu and Liou, 2005). The increase in expression of COX-2, mPGES-1, and PGIS, which is induced by inflammatory stimuli, leads to a corresponding increase in PGE_2_ and PGI_2_ levels.

PGE_2_ and PGI_2_ exert their biologic actions by binding to their respective receptors, namely E-Prostanoid [prostaglandin E_2_ receptor (EP)] and I-Prostanoid [prostacyclin receptor (IP)] receptors. Four subtypes of EP receptors (EP1–EP4) have been identified so far, although several splice variants of the EP3 receptor exist (for the characteristics of receptors see Tables 1 and 2). Prostanoid receptors are G-protein–coupled receptors with seven transmembrane domains, an extracellular N terminus, and an intracellular carboxyl terminus (Alexander et al., 2019). The seven transmembrane domains are connected by three intracellular and three extracellular loops (Narumiya et al., 1999; Sun and Li, 2018). The sequence homology between human and mouse IP, EP_1_, EP_2_, and EP_4_ receptors ranges from 79% to 88% (Narumiya et al., 1999; Mohan et al., 2012). These species differences in receptor sequences may have biologic and physiologic consequences (Narumiya et al., 1999). Compared with the synthetic pathways of prostanoids, it remains to be clarified which PG receptors are involved in each PG-elicited physiologic and pathophysiologic action, and this has mainly been due to lack of subtype-specific agonists and antagonists. In this review, by focusing on four subtypes of PGE_2_ receptors and PGI_2_ receptor, we summarize recent progress on molecular characterization of EP and IP receptors in various pathophysiologic processes.

**TABLE 1:** Signal-transduction mechanisms of EP1–4 and IP *receptor* subtypes References for this table could be found in *I. Introduction* section and in Breyer et al. (2019)

Receptor	Primary G-Protein	Classic Second Messenger	Other G-Protein
EP1	G_q_	Intracellular Ca^2+^	G_i_, G_12/13_
EP2	G_s_	cAMP	
EP3	G_i_	cAMP	G_q_, G_s_, G_12_
EP4	G_s_	cAMP	G_i_
IP	G_s_	cAMP	G_i_, G_q_

**TABLE 2:** Characteristics of EP1–4 and IP receptor subtypes in humans, rats, and mice

	Human	Rat	Mouse	References
EP1 Receptor				
Gene symbol	*PTGER1*	*Ptger1*	*Ptger1*	Funk et al., 1993; Watabe et al., 1993; Båtshake et al., 1995; Ishikawa et al., 1996; Okuda-Ashitaka et al., 1996; Boie et al., 1997
Gene ID	5731	25637	19216	
Chromosomal location	19p13.12	19q11	8 C2	
Number of exons	3	3	3	
Number of amino acids	402	405 Variant 366	405	
				
EP2 receptor				
Gene symbol	*PTGER2*	*Ptger2*	*Ptger2*	Regan et al., 1994b; Katsuyama et al., 1995; Boie et al., 1997; Nemoto et al., 1997; Smock et al., 1999
Gene ID	5732	81752	19217	
Chromosomal location	14q22.1	15p14	14 C1	
Number of exons	2	3	3	
Number of amino acids	358	357	362	
				
EP3 receptor^*a*^				
Gene symbol	*PTGER3*	*Ptger3*	*Ptger3*	Sugimoto et al., 1992, 1993; Irie et al., 1993; Takeuchi et al., 1993, 1994; Neuschäfer-Rube et al., 1994; Regan et al., 1994a; Kotani et al., 1995; Schmid et al., 1995; Boie et al., 1997; Kotani et al., 1997; Oldfield et al., 2001; Bilson et al., 2004
Gene ID	5733	24929	19218	
Chromosomal location	1p31.1	2q45	3 H4	
Number of exons	10	4	4	
Protein derived from splicing variants (UniProtKB accession number) and number of amino acids	EP3-I (P43115-1) 390	EP3*α* 365	EP3*α* 365	
	EP3-II (P43115-2) 388	EP3*β* 361	EP3*β* 361	
	EP3-III (P43115-3) 365	EP3*γ* 364	EP3*γ* 364	
	EP3-IV (P43115-4) 374	EP3*δ* 343		
	EP3-e (P43115-5) 418^*b*^			
				
EP4 receptor				
Gene symbol	*PTGER4*	*Ptger4*	*Ptger4*	(An et al., 1993; Honda et al., 1993; Bastien et al., 1994; Sando et al., 1994)^*c*^; Arakawa et al., 1996; Boie et al., 1997
Gene ID	5734	84023	19219	
Chromosomal location	5p13.1	2q16	15 A1	
Number of exons	7	3	3	
Number of amino acids	488	488	513	
				
IP receptor				
Gene symbol	*PTGIR*	*Ptgir*	*Ptgir*	Boie et al., 1994; Nakagawa et al., 1994; Namba et al., 1994; Sasaki et al., 1994
Gene ID	5739	292661	19222	
Chromosomal location	19q13.32	1q21	7 A2	
Number of exons	6	2	2	
Number of amino acids	386	416	415	

^*a*^ Ten transcripts produced by alternative splicing in human have been detected for the EP3 receptor. Here are presented the five human EP3 protein isoforms mostly found.

^*b*^ Correct value for EP3e amino-acid number, which has been described with mistake starting after Leu 373 in Schmid et al. (1995), Kotani et al. (1997), Lebender et al. (2018).

^*c*^ These publications present data for EP4 receptors under wrong nomenclature of EP2 receptors.

### A. Updated Aspects of General Characteristics of Prostaglandin E_2_ Receptors 1–4 and Prostacyclin Receptors

#### 1. Prostaglandin E_2_ Receptor 1

EP1 receptor was first cloned in 1993 by Watabe et al. (1993) (see Table 2). In rats, a splice variant of EP1 receptor was identified by Okuda-Ashitaka et al. (1996). The human and rat EP1 receptors share a sequence homology of 83%, whereas rat EP1 receptor is 96% homologous with mouse EP1 receptor (Funk et al., 1993; Watabe et al., 1993; Okuda-Ashitaka et al., 1996).

##### a. Prostaglandin E_2_ receptor 1 signaling

An increase in the concentration of intracellular Ca^2+^ is one of the main signaling events initiated upon EP1-receptor activation. Coupling of EP1 receptor to G_q_ with the subsequent activation of phospholipase C (PLC) and inositol triphosphate (IP_3_) synthesis has been considered as a possible mechanism for this Ca^2+^ mobilization (Table 1). Evidence showing that EP1 receptors couple to G_q_ was provided by the involvement of PLC in EP1-dependent human extravillous trophoblast migration, nuclear factor *κ* light chain enhancer of activated B cells (NF-*κ*B) activation in human endothelial kidney (HEK) cells, and increased bone formation in the rat osteoblast (Nicola et al., 2005; Tang et al., 2005; Neuschäfer-Rube et al., 2013). In addition, Ji et al. (2010) reported a dose-dependent increase of IP_3_ synthesis in HEK cells expressing human EP1 receptor in response to PGE_2_. Similarly, in the oocyte expression system, mouse EP1 receptors can stimulate Ca^2+^ mobilization through coupling to G_q_ (Tabata et al., 2002), and in glomerular mesangial cells of diabetic rats the EP1 signaling pathway is also associated with G_q_ activation (Ni et al., 2016).

The dependence of G_q_ coupling for EP1-receptor cell signaling has, however, been challenged by other studies (Woodward et al., 2011; Lebender et al., 2018). For instance, Watabe et al. (1993) showed that in CHO cells expressing the mouse EP1 receptor, the increase in intracellular Ca^2+^ was completely dependent on extracellular Ca^2+^ and was associated with limited IP_3_ formation. Two years later, the same group showed in the same expression system that the activation of EP1 receptor resulted in intracellular Ca^2+^ concentration increase through two mechanisms involving both extracellular and intracellular Ca^2+^ influx (Katoh et al., 1995).

In addition, other G-proteins have been associated with EP1 receptor. G_i_-mediated signaling was involved in the upregulation of hypoxia-inducible factor 1*α* that occurred upon the stimulation of human EP1 receptor in HEK cells (Ji et al., 2010). Furthermore, the downstream signaling of G_i_ involved the stimulation of phosphatidylinositol 3-kinase (PI3K)/protein kinase B (Akt)/mammalian target of rapamycin pathway. In another study, the same group found that human EP1 receptor upregulated the expression of nuclear receptor related-1 (Nurr1) in HEK cells by activating protein kinase (PK) A, cAMP response element-binding protein (CREB), and NF-*κ*B in a cAMP-independent manner (Ji et al., 2012). However, Rho signaling appears to be involved in the upregulation of Nurr1, which implies a possible coupling of the EP1 receptor to G_12/13_ (Ji et al., 2012).

Rat kidney highly expresses mRNAs and proteins of the EP1 and EP1-variant receptor, which possesses a transmembrane segment VII–like structure lacking an intracellular COOH-terminal tail. Overexpression of this EP1-variant in a CHO cell line inhibited EP1-mediated Ca^2+^ mobilization and EP4-mediated cAMP formation, suggesting that the rat EP1-receptor variant may be capable of inhibiting the signaling of other subtypes of EP receptors (Okuda-Ashitaka et al., 1996; Lebender et al., 2018).

#### 2. Prostaglandin E_2_ Receptors 2 and 4

EP2 and EP4 receptors share the same stimulatory G-protein (G_s_)–signaling pathway (Table 1), therefore we will discuss both in the same section. In fact, the EP4 receptor was detected by pharmacological studies in 1994 (Coleman et al., 1994), but before that it was believed that only three EP receptors existed. In some publications, the EP4 receptor was mistakenly designated EP2 receptor (see Table 2) until a fourth receptor, the genuine EP2 receptor, was cloned (Regan et al., 1994b), see Table 2. Despite the similarities in the functional coupling (discussed later) between EP2 and EP4 receptors, they share only ∼38% of the amino-acid sequence identity in the transmembrane domain. The human EP2 receptor consists of 358 amino acids, whereas the EP4 consists of 488 amino acids. The longer intracellular carboxyl terminus of the EP4 receptor (148 vs. 40) accounts for most of this difference, including the pattern of desensitization and internalization of the receptor in response to agonists (Desai et al., 2000). Additionally, the third intracellular loop of the EP4 receptor contains a stretch of 25 amino acids, which is not present in the EP2 receptor (Regan, 2003). Since these regions are important in coupling to G-proteins, it seems expected that there are differences in properties and/or the signal-transduction pathway between the two receptors.

##### b. Prostaglandin E_2_ receptor 2 and prostaglandin E_2_ receptor 4 signaling

Classically EP2 and EP4 receptors, which have broadly similar affinities (Ki of 1–5 nM) for the endogenous ligands PGE_2_ and PGE_1_ (Kiriyama et al., 1997; Abramovitz et al., 2000), have been shown to couple to G_s_ (Woodward et al., 2011). Stimulation of both receptors activates adenylate cyclase (AC), leading to an increase in cAMP and subsequent activation of cAMP-dependent protein kinase (PKA) and the transcription factor CREB (Honda et al., 1993; Regan et al., 1994b; Fujino et al., 2005). However, the signaling properties of these two receptors show some differences. The first indication was provided by Fujino et al. (2002), who found that the EP2 receptor could activate T-cell factor signaling mainly through a cAMP/PKA-dependent mechanism in contrast to the EP4 receptor, which was found to signal through a PI3K-dependent pathway.

Moreover, the amount of cAMP produced due to the activation of each receptor is different. In the same study described above, Fujino et al. (2002) reported that at the same level of receptor expression, HEK cells stably expressing human EP4 receptor produced only 20%–50% of the amount of cAMP produced by the cells expressing human EP2 receptors. Furthermore, the same group reported that, in addition to coupling to G_s_, human EP4 receptor can couple to G_i_ to activate PI3K signaling (Fujino and Regan, 2006). Another difference between EP2 and EP4 receptors is the rapid agonist-induced desensitization and internalization that occur with the EP4 receptor but not with the EP2 receptor (Nishigaki et al., 1996; Desai et al., 2000). Together the coupling to G_i_ and the short-term desensitization of EP4 receptors can, in part, justify the lesser amount of cAMP produced upon the stimulation of EP4 compared with EP2 and hence might seem to limit the efficiency of the functional coupling of EP4 receptor to cAMP/PKA compared with EP2 receptor. Furthermore, a positive feedback loop between PGE_2_ synthesis and EP2-receptor expression was detected in human fibroblasts and colon cancer cells (Sagana et al., 2009; Hsu et al., 2017).

In addition to the classic cAMP/PKA pathway, cAMP can also result in the activation of PKA-independent pathways, such as, for instance, the exchange proteins directly activated by cAMP (Epac1 and 2). There is accumulating evidence that EP2 and EP4 receptors signal through the Epac pathway. In human lung fibroblasts, Epac mediated the antiproliferative effects of the EP2 receptor (Haag et al., 2008), whereas in mouse neuronal cultures, EP2-receptor stimulation protected against hemin-induced neurotoxicity through activation of the Epac pathway (Mohan et al., 2015). In rat microglia, EP2-receptor activation (via Epac-signaling pathways) induced the proinflammatory mediators, COX-2, inducible nitric oxide synthase (iNOS), interleukin (IL)-1*β*, and IL-6 while decreasing the induction of proinflammatory tumor necrosis factor (TNF) *α* and the chemotactic factors [chemokine ligand (CCL)] CCL3 and CCL4 (Quan et al., 2013). EP4 receptor induced cystic-fibrosis transmembrane-regulator anion secretion by a mechanism involving cAMP/Epac signaling through PLC-induced Ca^2+^ mobilization in human bronchial epithelial cells (Ivonnet et al., 2015). In other situations, PGE_2_ regulates the function of Jurkat T cells (immortalized human T lymphocyte cell line) through the EP4-PKA/Epac pathway, increasing the expression of the immunoglobulin, T-cell immunoglobulin mucin-3 (Yun et al., 2019). PGE_2_ compromised bone formation by activating EP2/4-cAMP-Epac–signaling pathway via Akt phosphorylation in human bone-marrow cells (Mirsaidi et al., 2017). In mouse bone marrow–derived macrophages, PGE_2_ increased IL-1*β* due to activation of EP2/EP4 and stimulation of PKA and/or Epac in response to infection (Martínez-Colón et al., 2018).

Several studies now show that PI3K/extracellular receptor kinase (ERK) pathway is important for signaling by EP2 and EP4 receptors but not always in the same cell type. Fujino et al. (2003) showed that in HEK-293 cells stably transfected with the human EP2 and EP4 receptors, EP4 but not EP2 receptors induced ERK activation by a PI3K-dependent pathway. Subsequent studies further confirmed the link between EP4 receptors and ERK activation in human (Takahashi et al., 2015; Li et al., 2017c), rat (Mendez and LaPointe, 2005; Frias et al., 2007), and mice (Pozzi et al., 2004; Nandi et al., 2017; Ying et al., 2018) cells (cardiomyocytes, cancer cells, etc.). This PI3K-pathway signaling associated with Ca^2+^ influx after EP4-receptor activation has been described to promote cell migration in human oral-cancer cell lines (Osawa et al., 2020). Opposing the findings by Fujino et al. (2003), EP2-receptor activation can also activate PI3K signaling, leading to the differentiation of type 1 helper T (Th1) cells (Yao et al., 2009). EP2 receptors were also shown to activate PI3K in human colorectal cancer (Hsu et al., 2017), mouse dendritic (Yen et al., 2011), and rat brain glioma cells (Park et al., 2009). In mouse dendritic cells, this activation was cAMP-dependent and led to ERK phosphorylation (Yen et al., 2011).

EP2 and EP4 receptors have been shown to exert some actions by associating with *β*-arrestin (Hirata and Narumiya, 2011). Complexing of G-protein–coupled receptors with *β*-arrestin leads to receptor internalization and desensitization (Wendell et al., 2020). These events were originally considered to be a means of terminating receptor signaling until evidence (for *β*_2_-adrenergic receptors) was provided that *β*-arrestin mediates intracellular G-protein–independent signaling pathways (Luttrell et al., 1999). Likewise, in mouse brain microglia, PGE_2_ inhibited the production of IL-10 through EP2/*β*-arrestin pathway independent of G-protein signaling (Chu et al., 2015). In human colorectal cancer cells and mouse keratinocytes, activation of either the EP4 or EP2 receptor leads to transactivation of epidermal growth factor receptor through *β*-arrestin/Src pathway (Buchanan et al., 2006; Chun et al., 2010). Furthermore, in a mouse model of portal hypertensive gastropathy, PGE_2_ reduced mucosal apoptosis through the EP4/*β*-arrestin/Src/epidermal growth factor receptor/Akt cascade (Tan et al., 2017).

#### 3. Prostaglandin E_2_ Receptor 3

Among EP receptors, the EP3 receptor was the first to be cloned (Sugimoto et al., 1992, see Table 2), although this receptor is known to express various isoforms (Sugimoto et al., 1993). In humans, 10 different mRNA splice variants have been detected (Regan et al., 1994a; Kotani et al., 1995, 1997; Schmid et al., 1995; Kotelevets et al., 2007). At first, it was believed that these variants resulted in eight different EP3-receptor isoforms (Kotelevets et al., 2007), but subsequently three of these mRNA variants were recognized as noncoding (NR_028294.2, NR_028292.2, NR_028293.2). Therefore, it became clearer that only five receptor isoforms exist, which were named EP3-I, EP3-II, EP3-III, EP3-IV, and EP3-e. EP3-I isoform has three splice variants that are different in the 3′-untranslated region. These variants are designated EP3-Ia, EP3-Ib, and EP3-Ic (Regan et al., 1994a; Kotelevets et al., 2007). In all EP3 isoforms, the first 359 amino acids across the seven transmembrane helices are identical. However, the number of amino acids in the carboxy terminal of each isoform varies between 6 and 59 (Kotani et al., 1995; Bilson et al., 2004). Consequently, each isoform can initiate distinct signaling pathways, which points to the importance of the carboxyl-terminal region (Irie et al., 1994; Schmid et al., 1995; Jin et al., 1997; Woodward et al., 2011). In addition, this variation in the C-terminal domain leads to differences in agonist-induced internalization (Bilson et al., 2004). In rats, four splice variants were cloned (Takeuchi et al., 1993, 1994; Neuschäfer-Rube et al., 1994; Oldfield et al., 2001), whereas in mice, three isoforms exist, which are EP3*α*, EP3*β*, and EP3*γ* (Sugimoto et al., 1992, 1993; Irie et al., 1993). Similar to humans, the isoforms in rats and mice arise because of differences in the carboxyl-terminal tails (Takeuchi et al., 1994; Negishi et al., 1996; Oldfield et al., 2001). The mouse EP3*α* isoform is a homolog of human EP3A (International Union of Basic and Clinical Pharmacology Committee on Receptor nomenclature: EP3-I) receptor (Regan et al., 1994a). Notably, sometimes the same isoform has been assigned different terms by different investigators (Woodward et al., 2011; Lebender et al., 2018); however, International Union of Basic and Clinical Pharmacology Committee on Receptor nomenclature should be followed.

##### c. Prostaglandin E_2_ receptor 3 signaling

The major signal-transduction pathway for EP3 receptor is considered to be inhibition of AC via G_i_ coupling (Sugimoto and Narumiya, 2007). However, other signal-transduction pathways have been attributed to EP3 receptors. Some human EP3 isoforms have been associated with increasing intracellular Ca^2+^, generating IP_3_, and/or coupling to G_s_ proteins (An et al., 1994; Kotani et al., 1995; Schmid et al., 1995). Concerning mouse EP3-receptor isoforms, Yokoyama et al. (2012) demonstrated that EP3*β* can induce AC superactivation through coupling to G_q_/PLC/Ca^2+^ pathway in COS-7 cells but not in HEK cells. Furthermore, mouse EP3*γ* expressed in CHO cells was shown to couple to both G_i_ and G_s_ (Negishi et al., 1996). Mouse EP3*α*, EP3*β*, and EP3*γ* were found to couple to Rho activation via a pertussis toxin–insensitive G-protein (Hasegawa et al., 1997), possibly G_12_ (Hasegawa et al., 1997; Macias-Perez et al., 2008; Lu et al., 2015).

In a clear demonstration of differences in signaling between EP3 isoforms, Israel and Regan (2009) showed that, although human EP3-II and EP3-III isoforms can induce ERK activation in HEK cells, EP3-Ia could not. Furthermore, the underlying mechanisms of ERK phosphorylation were different. EP3-III induced a cascade involving G_i_/PI3K/PKC/Src, whereas EP3-II–signaling pathway did not involve PI3K and PKC. The significance of these differences is reflected in the variation of the downstream gene expression stimulated by these isoforms.

#### 4. Prostacyclin Receptor

The IP receptor is activated by the endogenous ligand PGI_2_ and was cloned in 1994 (Boie et al., 1994; Nakagawa et al., 1994), see Table 2. The human IP and rat IP receptors share a sequence homology of 79%, whereas the rat IP receptor is 94% homologous with the mouse IP receptor.

##### d. Prostacyclin receptor signaling

The IP receptor has also been recognized to classically couple to the G_s_ protein (Table 1). Therefore, its activation results in cAMP production and the subsequent activation of PKA (Hirata and Narumiya, 2011). In addition, some studies showed the ability of IP receptor to couple to G_q_ and/or G_i_ as well (Woodward et al., 2011). The activation of mouse IP receptor expressed in CHO cells resulted in the production of both cAMP and IP_3_, suggesting coupling to G_s_ and G_q_, respectively (Namba et al., 1994). In human erythroleukemia cell line and mouse adipocytes, IP-receptor stimulation produced a cAMP-independent increase in intracellular Ca^2+^, implying that this increase occurs simultaneously with G_s_ coupling (Vassaux et al., 1992; Schwaner et al., 1995). Lawler et al. (2001) reported that mouse IP receptor expressed in HEK cells and mouse erythroleukemia cells could couple to G_i_ and G_q_ and increase IP_3_ and intracellular Ca^2+^ in addition to G_s_. However, the G_i_- and G_q_-mediated effects required the presence of cAMP and activated PKA as a prerequisite. Interestingly, in other cell types, mouse IP-receptor activation was not associated with G_i_-dependent responses (Chow et al., 2003). Taken together, the difference between these findings suggests that the ability of mouse IP to couple to G_i_ depends on cell type involved (Hirata and Narumiya, 2011; Woodward et al., 2011). Similarly, human IP receptor expressed in CHO, HEK, and SK-N-SH did not show any evidence of G_i_ coupling (Chow et al., 2003).

In addition to classic signaling through G-proteins, the IP receptor has been shown to activate a family of transcription factors called peroxisome proliferator–activated receptors (PPARs), which can regulate cell function through nongenomic and genomic pathways (Clapp and Gurung, 2015). According to Falcetti et al. (2007), human IP expressed in HEK cells produced antiproliferative effects by activating PPAR-*γ* independently of cAMP. On the other hand, IP-receptor activation of potassium channels in human pulmonary artery smooth muscle occurred through PPAR-*β*/*δ* but in a PKA-dependent manner (Li et al., 2012). Furthermore, activation of IP induced migration of human breast-cancer cells and was reported to stimulate the PI3K/P38 pathway independently of PKA (Allison et al., 2015).

### B. Single-Nucleotide Polymorphisms and Dimerization of Prostaglandin E_2_ Receptors 1–4 and Prostacyclin Receptors

Other characteristics and information about these receptors (EP1–4, IP), such as subtypes, isoforms, single-nucleotide polymorphism (SNP), and dimerization, have yet to be fully elucidated. A putative second IP-receptor subtype was suggested by Wilson and colleagues (2011) in human-airway epithelial cells, although molecular evidence for the IP2 subtype is currently lacking. Furthermore, some SNPs have been described for EP and IP receptors (Cornejo-García et al., 2016). *PTGER* SNPs are associated with different pathologies: EP2 in essential hypertension (Sato et al., 2007), EP3 in Stevens-Johnson syndrome/toxic epidermal necrolysis (Ueta et al., 2015; Mieno et al., 2020), and in asthma (Park et al., 2007) as well as EP4 in African Americans with inflammatory bowel disease (Brant et al., 2017), in gastric cancer (Heinrichs et al., 2018), and in multiple sclerosis (Matesanz et al., 2012). Similarly, a single mutation in *PTGIR* reduces cAMP production (Stitham et al., 2007) and is associated with platelet activation and cerebral infarction (Shimizu et al., 2013). The prostanoid receptors, including IP and EP receptors, are known to have the ability to form homodimers or heterodimers (Midgett et al., 2011; Matsubara et al., 2017). Relatively few studies have been devoted to this issue, making comparisons between humans and rodents concerning homodimers and heterodimers difficult (Giguère et al., 2004; McGraw et al., 2006; Osborne et al., 2009; Ibrahim et al., 2013). The human IP receptor was the first to be described to form heterodimers, most interestingly with its physiologic opponent, the TP receptor, leading to unexpected TP-mediated cAMP formation, binding of isoprostanes to the heterodimer, and TP-receptor internalization induced by PGI_2_ (Wilson et al., 2004, 2007). By itself, human IP forms homo-oligomers via disulfide bonds, which might be essential for receptor trafficking to the cell surface (Giguère et al., 2004). Coexpression of EP1 and *β*-adrenergic receptors results in formation of heterodimers, which may contribute to *β*-agonist resistance in asthma (McGraw et al., 2006). The mouse EP2 receptor has been found to form heterodimers with the calcitonin receptor, thereby reducing its ability to induce Ca^2+^ flux (Matsubara et al., 2017).

### C. Crystal Structures of Human Prostaglandin E_2_ Receptor 3 and Prostaglandin E_2_ Receptor 4

Recently, Kobayashi and his colleagues reported the structural basis for prostanoid receptor ligand binding by crystallization of the human EP4 receptor with its antagonist ONO-AE3-208 and an inhibitory anti-EP4 antibody (Toyoda et al., 2019) and also crystallization of the human EP3 receptor–PGE_2_ complex (Morimoto et al., 2019). Two more papers regarding structures of EP3 (Audet et al., 2019) and TP (Fan et al., 2019b) receptors were published back-to-back. These papers provide us with several important insights in developing prostanoid receptor–targeted drugs. Firstly, the ligand-binding pocket, which is open toward the extracellular direction in *β*-adrenergic receptor, is covered by the *β*-hairpin structure of the second extracellular loop region (interestingly, the sequences within these regions are highly conserved among prostanoid receptors). In addition, the ligand-binding pocket is open toward the phospholipid membrane, and the pore entrance consists of the first and seventh helix regions (Toyoda et al., 2019). This suggests that the ligand for prostanoid receptor could enter the pocket by way of the plasma membrane and not via direct access from extracellular space like for the *β*-adrenergic ligand (Hollenstein, 2019). Secondly, an EP4 antagonist, ONO-AE3-208, was shown to directly bind to the entrance region of the ligand-binding pore by interacting with several amino acids within the seventh helix region of EP4 receptor, including R316^7.36^, which had previously been predicted as a potential binding site for carboxylic acid of prostanoid ligand (Toyoda et al., 2019). Thirdly, the natural ligand, PGE_2_ more deeply enters the ligand-binding pocket of the EP3 receptor (Morimoto et al., 2019); *ω*- and *α*-chain moieties of the PGE_2_ interact with several amino acids within sixth and seventh helices and those within second and seventh helices as well as second extracellular region, respectively. Moreover, polar functional groups in the cyclopentane ring are recognized by amino acids within first and second helices, which was previously suggested by a series of studies to identify the receptor domains important for ligand recognition by using chimeric and point-mutated prostanoid receptors (Kobayashi et al., 1997, 2000). Such structural information regarding interactions between specific amino-acid residues of the PG receptor and the particular structure of their ligands strongly promotes our understanding regarding how lipid-natured PG molecules access into specific PG receptors in plasma membranes and will facilitate the development of more specific compounds for PG-related diseases. Knowledge from the crystal structure data will also aid in our understanding of the potential functional consequence of polymorphisms in prostanoid receptors, particularly for the EP3 receptor, which has been linked with certain diseases (Jeffcoat et al., 2014; Ueta, 2018).

### D. Allosteric Modulators and Biased Ligands

Although classic EP- and IP-receptor agonists and antagonists have an orthostatic mode of binding (i.e., sharing the binding site with the natural ligand) allosteric modulators—both positive and negative—have been identified for the EP2 receptor (Jiang et al., 2010, 2018, 2020). Likewise, a positive modulator for the IP receptor (Yamamoto et al., 2017) and a negative modulator of EP4 (Leduc et al., 2013) have been reported. It is anticipated that such compounds might be more potent and selective, metabolically stable, and/or less costly than traditional prostaglandin receptor ligands.

Another option to hone the pharmacodynamic profiles of ligands is to introduce biased signaling properties, also referred to as functional selectivity. For instance, in cells overexpressing human EP4 receptors, PGE_2_ potently activates G_s_ proteins, whereas PGF_2*α*_ and PGE_1_ alcohol are biased toward activating G_i_ and *β*-arrestin, respectively (Leduc et al., 2009). Along the same lines, PGE_1_ and PGE_3_ were found to be biased EP4 ligands showing lower efficacy than PGE_2_ to stimulate T-cell factor/*β*-catenin–mediated activity, which is consistent with their antineoplastic properties, whereas they maintain full activity with regard to cAMP formation (Araki et al., 2017). A synthetic biased EP2 agonist showed 1000-fold increase in its potency stimulating cAMP formation but more than 50-fold reduced potency in *β*-arrestin recruitment as compared with PGE_2_ (Ogawa et al., 2016).

## II. Immune System

### A. Effects of Prostaglandin E_2_ Receptors 1–4 and Prostacyclin Receptor on Human and Mouse Immune Cells

#### 1. T Lymphocytes

For a long time, PGE_2_ through EP2 and EP4 activation and downstream cAMP-PKA signaling was believed to suppress both mouse and human T-cell activation and primary cytokine production [e.g., IL-2 and interferon (IFN)-*γ*] in response to antigens or mitogens (Brudvik and Tasken, 2012). However, this concept has been challenged recently with evidence that these receptors may actually act as an immune activator (Yao and Narumiya, 2019). In this respect, activation of EP2 and/or EP4 receptor by PGE_2_ or their selective agonists facilitated mouse Th1 cell differentiation, and this was prevented by their respective antagonists (Yao et al., 2009, 2013). Interestingly, these effects involve both cAMP-PKA– and PI3K-Akt–signaling pathways (Yao et al., 2009, 2013). Findings linking PGE_2_ and EP2/4 receptors to human autoimmune inflammatory diseases have been revealed by genetic association studies, which positively link enhanced PGE_2_ signaling to IL-23/ type 17 helper T cell (Th17) signature genes and disease severity (Yao and Narumiya, 2019).

In other studies, an EP1-receptor agonist, ONO-DI-004, increased Th1 cell differentiation in wild-type mouse T cells but had little effect on differentiation of EP1-deficient Th1 cells (Nagamachi et al., 2007). On the one hand, PGE_2_ and EP2/EP4-receptor agonists inhibit human Th1 cells by reducing the expression of the transcription factor T-box expressed in T cells and the production of the cytokine IFN-*γ* (Boniface et al., 2009; Napolitani et al., 2009). On the other hand, PGE_2_ favors the production of type 2 cytokines IL-4 and IL-5 from human T cells but does not affect mouse type 2 helper T cell (Th2) cytokine production in vitro (Hilkens et al., 1995). Moreover, PGE_2_ and EP2/EP4-receptor agonists significantly promote IL-17 and IL-22 production from both mouse and human Th17 cells because of induction of IL-23 and IL-1*β* receptors, an effect counteracted by their respective antagonists (Hilkens et al., 1995; Chizzolini et al., 2008; Boniface et al., 2009; Napolitani et al., 2009; Yao et al., 2009; Chen et al., 2010; Lee et al., 2019).

Although Th1, Th2, and Th17 cells modulate various proinflammatory and antimicrobial responses, regulatory T cells (Treg) usually act in an anti-inflammatory manner. It was previously reported that PGE_2_ promotes both mouse and human Treg cell differentiation, especially in the tumor microenvironment (Baratelli et al., 2005; Sharma et al., 2005). In contrast, it was recently found that PGE_2_ can also inhibit both mouse and human Treg development in vitro, which is mimicked by EP2- and EP4-receptor agonists and mediated by the cAMP-PKA pathway (Hooper et al., 2017; Li et al., 2017b; Maseda et al., 2018).

Similar to PGE_2_, PGI_2_ can also activate the cAMP pathway and regulate T-cell function, but depending on the context of disease model being investigated, it can have proimmunomodulatory or anti-immunomodulatory effects, including on T cells (Dorris and Peebles, 2012). In one of the early studies, IP-receptor agonists were reported to inhibit Th1 cell differentiation in vitro via a cAMP-dependent suppression of NF-*κβ* (Zhou et al., 2007a). In subsequent experiments by the same group, antigen- or IL-33–dependent Th2 cell function and allergic lung inflammation could be elicited by prostacyclin analogs both in vitro and in vivo, (Zhou et al., 2007b, 2018a). A critical role of the IP receptor in response to a fungal challenge in mouse lungs was also confirmed in PTGIR null mice, in which increased IL-5 and IL-13 responsiveness of CD4+ T cells to Alternaria sensitization, typically a response requiring IL-33, was observed (Zhou et al., 2018a). The situation appears to differ for other types of antigen reactions, in which activation of IP receptor by the agonist iloprost enhances Th1 differentiation (suppresses Th2 differentiation) in vivo and promotes Th1 cell–mediated inflammatory responses in a mouse model of contact hypersensitivity. This is also consistent with PTGIR null mice displaying much less contact hypersensitivity (Nakajima et al., 2010). Moreover, the IP-receptor agonists iloprost and cicaprost facilitated mouse Th17 cell differentiation and function and increased IL-17 and IL-22 production from human Th17 cells (Truchetet et al., 2012; Zhou et al., 2012). Collectively, the new findings indicate that PGE_2_ and PGI_2_ can use the cAMP pathway to promote inflammatory effector T-cell (e.g., Th1, Th17, and Th22) responses in vivo, although they primarily downregulate T cell–receptor activation. Thus, the role of G_s_-coupled prostanoid receptors on T-cell function, although compelling, is complex and remains somewhat conflicting. Immunomodulation appears to be dependent on the animal species, the type of inflammatory disease studied, and, to an extent, whether data has been collected in vitro or in vivo.

#### 2. Innate Lymphoid Cells

Innate lymphoid cells (ILCs) are a group of cells that secrete large amounts of prototypic T-cell cytokines, such as IFN-*γ*, IL-17, and IL-4, in response to appropriate stimuli, but, unlike T cells, they do not express T-cell receptors (Vivier et al., 2018). ILCs exert their functions by producing different cytokines (e.g., ILC1 cells produce IFN-*γ*, and ILC2 cells secrete IL-4, IL-5, and IL-13, whereas ILC3 cells mainly produce IL-17 and IL-22). In mice, PGE_2_ and an EP4-receptor agonist, L-902,688, increased the production of IL-22 from ILC3 cells in vitro. Inhibition of PGE_2_ production using a nonselective COX inhibitor indomethacin or blockade of EP4 signaling using either a selective EP4-receptor antagonist L-161,982 or deletion of EP4 receptor on T cells and ILCs reduced IL-22 production in vivo (Duffin et al., 2016). PGE_2_ also promotes IL-22 production from human ILC3 (Duffin et al., 2016). In contrast to experiments in ILC3 cells, an EP4-receptor agonist, PGE_2_-alcohol, mimicked PGE_2_ inhibition of ILC2 cytokine production in mice, whereas the EP2-receptor agonist, butaprost, did not reduce ILC2 cytokine production significantly in the same setting (Zhou et al., 2018b). Similarly, although the EP4-receptor agonist L-902,688 mimicked PGE_2_ suppression of cytokine production from human ILC2, an EP2-receptor agonist, butaprost, had little effect (Maric et al., 2018).

#### 3. Dendritic Cells

Dendritic cells (DCs) are important cells for regulating innate immunity and for presenting antigens to T cells, with PGE_2_ playing a critical role in the maturation of DCs (Kalinski, 2012; Jia et al., 2019). PGE_2_ is required for the migration of DCs, allowing their homing to draining lymph nodes, and this is mimicked by EP4-receptor agonist in mouse DCs and by both EP2- and EP4-receptor agonists in human monocyte-derived DCs (Kabashima et al., 2003; Legler et al., 2006). Engagement of EP2 and EP4 receptors by their agonists promotes the production of the proinflammatory cytokine IL-23, which is critical for development and maturation of Th17 cells by both mouse and human DCs. These effects were found to be mediated through activation of the cAMP-PKA pathway and transcription factors CREB, NF-*κ*B, and C/AATT enhancer-binding protein *β* (Kocieda et al., 2012;Shi et al., 2015; Ma et al., 2016). Moreover, EP2- and EP3-receptor agonists induced the generation of human tolerogenic DCs characterized by the induction of high levels of the immunosuppressant, IL-10, whereas an EP4-receptor agonist favored the development of inflammatory DC by promoting the production of IL-23 and Th17 polarization (Flórez-Grau et al., 2017). In other studies, the EP3-receptor agonist ONO-AE-248 was observed to inhibit the chemotaxis and costimulatory molecule expressions of mouse DCs in vitro and restricted DC cell function to fine-tune excessive skin inflammation in vivo (Shiraishi et al., 2013). A number of studies have reported a suppressive effect of the IP receptor in DCs. The IP-receptor agonists cicaprost, iloprost, and treprostinil inhibited the production of proinflammatory chemokines and cytokines from human monocyte-induced DCs stimulated by lipopolysaccharides (LPSs) or TNF-*α* (Hung et al., 2009; Yeh et al., 2011; Wang et al., 2017a). Furthermore, iloprost also suppressed mouse-airway DC function to inhibit Th2 differentiation and thereby reduced allergic lung inflammation in a mouse model of asthma (Idzko et al., 2007).

#### 4. Macrophages

In human macrophages, PGE_2_-EP2/EP4-cAMP signaling inhibits inflammatory cytokine production (e.g., TNF-*α*, IL-1*β*) and phagocytosis, promoting an M2-phenotype associated with an increase in IL-10 production. For example, the EP4-receptor agonist L-902,688 was found to inhibit human lung macrophage production of TNF-*α*, whereas the EP2 agonist butaprost was 400× less potent, suggesting a major role for the EP4 over the EP2 receptor in this study (Gill et al., 2016). In other studies, EP4 receptors may play a role in the resolution phase of inflammation (Sokolowska et al., 2015). It was found that PGE_2_ and the EP4 agonist CAY10598 inhibited the activation of nucleotide-binding oligomerization domain-like receptors family pyrin domain-containing 3 inflammasome and IL-1*β* production in human primary monocyte-derived macrophages, whereas EP4-receptor antagonist GW627368X (Wilson et al., 2006) or EP4 knockdown reversed the PGE_2_-mediated nucleotide-binding oligomerization domain-like receptors family pyrin domain-containing 3 inhibition (Sokolowska et al., 2015). Binding of advanced glycation end-products on human monocytes/macrophages activated T cells and reduced allograft survival, a process that was inhibited by PGE_2_, the EP2-receptor agonist ONO-AE1-259, and the EP4-receptor agonist ONO-AE1-329. The inhibitory effects of PGE_2_ were prevented by either by AH6809 (EP1/2 and DP1 antagonist) or AH23848 (EP4/TP antagonist) (Takahashi et al., 2010). In other situations, however, PGE_2_, butaprost, and the EP4 agonist CAY10598 could inhibit 1,25-dihydroxy vitamin D3–induced production of human cationic antimicrobial protein-18 from human macrophages during *Mycobacterium tuberculosis* infection (Wan et al., 2018). Given that responses could partially be reversed by AH6809 (EP1/2 and DP1 antagonist) or L-161,982 (EP4 antagonist) but not L-798106 (EP3 antagonist) suggests a dual role for EP2 and EP4 receptors in restraining the innate immune response and prolonging microbial survival. Likewise, the killing of *Klebsiella pneumoniae* by rat alveolar macrophages was prevented by PGE_2_ and treprostinil, with both agents acting in part through EP2 receptors (Aronoff et al., 2007).

As already documented with EP2/4 receptor agonists, PGI_2_ analogs (iloprost, beraprost, treprostinil, and ONO-1301) are also able to suppress LPS-induced proinflammatory monocyte chemoattractant protein-1 (MCP-1) production from human monocytes and macrophages (Tsai et al., 2015). More recently, Aoki and colleagues recently reported that inactivation of the PGE_2_-EP2- NF-*κ*B–signaling pathway in mouse macrophages reduced macrophage infiltration and proinflammatory cytokine (e.g., MCP-1) production, leading to the prevention of intracranial aneurysms. They found that administration of EP2-receptor antagonist PF-04418948 (af Forselles et al., 2011) in rats reduced macrophage infiltration and intracranial aneurysm formation and progression (Aoki et al., 2017a,b). The IP-receptor agonist cicaprost stimulated vascular endothelial growth factor secretion but inhibited MCP-1 production from TNF-*α–*treated human monocyte-derived macrophages, whereas administration of an IP-receptor antagonist, RO3244794 (Bley et al., 2006), significantly reduced neovascularized lesion area in mouse choroidal neovascularization model (Woodward et al., 2019). In the same experimental setting, such effects could be mimicked by PGE_2_ acting in part on EP4 receptors (as determined by the EP4 antagonist, GW627368). Taken together, this suggests that both IP and EP4 receptors may play a role macrophage-driven neovascularization.

#### 5. Neutrophils

To kill invading microbes, neutrophils release their nuclear contents in an NADPH oxidase and reactive oxygen species –dependent manner, with neutrophil extracellular traps (NETs) playing a critical role in killing bacteria, fungi, or viruses by physically trapping them. PGE_2_ plays a key role in this process and has been shown to inhibit human neutrophil function (such as superoxide production, migration, and antimicrobial peptide release), an effect that was prevented by AH6809 (EP1/2 and DP1 antagonist) but not by the EP4 receptor–selective antagonist ONO-AE2-227 (Turcotte et al., 2017). Other studies have confirmed that PGE_2_ inhibits human NET formation through EP2 and EP4 receptors in vitro and via the EP2 receptor in vivo, in which the EP2 agonist butaprost suppressed NET formation in mice (Shishikura et al., 2016). Both mouse and human neutrophils overexpress COX-2 and PGE_2_ post–bone marrow transplantation (Ballinger et al., 2006) and exhibit defective bacterial killing due to reduced NET formation (Domingo-Gonzalez et al., 2016). Reduced NET formation after bone-marrow transplantation in mice and humans could be restored by COX inhibitors or an EP2-receptor antagonist (PF-04418948) plus an EP4-receptor antagonist (ONO-AE3-208) (Domingo-Gonzalez et al., 2016). Activation of EP4 receptor prevented endotoxin-induced mouse neutrophil infiltration into airways (Konya et al., 2015). Similarly, EP4 receptor mediates PGE_2_-induced enhancement of human pulmonary microvascular barrier function against neutrophil infiltration (Konya et al., 2013). Taken together, these relatively recent studies provide good evidence that PGE_2_, through activation of both EP2 and EP4 receptors, suppresses the immune response of neutrophils. Thus, manipulation of these receptors therapeutically may prove useful in blocking pathologic NETosis in autoimmune diseases and/or aid the host response to infection.

#### 6. Eosinophils and Mast Cells

Human and mouse eosinophils express EP2 and EP4 receptors, which mediate the effects of PGE_2_ by blocking eosinophil responses, such as degranulation, chemotaxis, and production of reactive oxygen species (Mita et al., 2002; Sturm et al., 2008; Luschnig-Schratl et al., 2011). The underlying signaling pathways appear to involve PI3K, phosphoinositide-dependent kinase 1, and PKC but not the cAMP/PKA pathway (Luschnig-Schratl et al., 2011; Sturm et al., 2015). PGE_2_ via the EP4 receptor inhibited the interaction of eosinophils with human pulmonary endothelial cells in vitro, including adhesion and transmigration (Konya et al., 2011). In other experimental settings, the EP2 receptor appears to inhibit the mobilization of eosinophils from guinea-pig bone marrow and allergen-induced eosinophil recruitment to mouse lung (Sturm et al., 2008) and is involved in IgE-dependent human-airway constriction in vitro by inhibiting mast-cell activation (Safholm et al., 2015).

In human mast-cell lines and primary cord blood-derived mast cells, EP2-, EP3-, and EP4-receptor proteins are expressed (Feng et al., 2006; Torres-Atencio et al., 2014). PGE_2_ counteracted the hyperosmolar-induced degranulation of these mast cells via EP2 and EP4 receptors (Torres-Atencio et al., 2014). Human lung mast cells likewise express both EP2 and EP4-receptor mRNA, but it is the EP2 receptor that predominantly mediates the inhibitory effect of PGE_2_ on histamine release in vitro (Kay et al., 2013). In contrast, EP3-receptor activation causes migration, adhesion, antigen-dependent degranulation, and IL-6 release of mouse mast cells (Nguyen et al., 2002; Weller et al., 2007; Sakanaka et al., 2008) and potentiates histamine release in human peripheral blood-derived mast cells (Wang and Lau, 2006).

Similar to PGE_2_, PGI_2_ attenuates the locomotion of human peripheral blood eosinophils and guinea-pig bone-marrow eosinophils via IP-receptor activation (Konya et al., 2010; Sturm et al., 2011). Unlike EP2/EP4 signaling in eosinophils, the inhibitory-effect PGI_2_ is mediated by intracellular cAMP. Accordingly, endothelium-derived PGI_2_ controls eosinophil-endothelial interaction and promotes the barrier function of lung endothelial cells to limit eosinophil adhesion and transendothelial migration (Konya et al., 2010). Thus, these data explain previous findings that deletion of the IP receptor in mice augmented allergen-induced eosinophilia in the lung and skin and enhanced airway remodeling (Takahashi et al., 2002; Nagao et al., 2003).

#### 7. Hematopoietic Stem/Progenitor Cell and Leukemia

PGE_2_ treatment of hematopoietic stem cell (HSC)/hematopoietic progenitor cell (HPC) from mice and humans promotes survival, proliferation, and engraftment in vitro (Hoggatt et al., 2009) These effects are recapitulated by the EP2-receptor agonist ONO-AE1-259 or the EP4-receptor agonist ONO-AE1-329, which increased mouse and human HSC/HPC colony formation and long-term bone-marrow reconstitution capacity of Lineage^−^Sca-1^+^c-Kit^+^ cells (Ikushima et al., 2013; Wang et al., 2017b). Moreover, treatment of bone-marrow mesenchymal progenitor cells with PGE_2_ or the EP4-receptor agonist significantly increased their ability to support HSPC colony formation (Ikushima et al., 2013). Conversely, treatment with COX inhibitors increased HPCs in peripheral blood of both mice and humans. Similarly, administration of selective EP4-receptor antagonists L-161,982 or AH23848 expanded bone-marrow HPCs and enhanced HPC mobilization in mice induced by GM-CSF, whereas administration of selective EP4-receptor agonist (L-902,688), rather than EP1-, EP2-, or EP3-receptor agonists, reduced HPC mobilization (Hoggatt et al., 2013).

PGE_1_ treatment impaired the persistence and activity of leukemic stem cells in a preclinical mouse chronic myelogenous leukemia (CML) model and a xenograft model of transplanted CML patient CD34^+^ HSCs/HPCs, and a nonselective EP2/EP3/EP4 agonist, misoprostol, conferred similar protection against CML, suggesting potential therapeutic strategy of CML by using PGE_1_ or misoprostol (Li et al., 2017a). Along the same lines, the breakpoint cluster region-Abelson inhibitors imatinib and nilotinib were recently described to enhance PGE_2_ biosynthesis in monocytes of healthy volunteers and CML patients, an effect that might contribute to their clinical efficacy in the treatment of CML (Bärnthaler et al., 2019).

### B. Roles of Prostaglandin E_2_ in Human Autoimmune Diseases and Relevant Mouse Models

Given the critical effects of PGE_2_ on immune cell activation and function, this lipid mediator has been reported to be linked to the development and pathogenesis of various inflammatory diseases in various animal models. Simultaneously, genome-wide associated studies have suggested a role for the PGE_2_ pathway in immune-related human diseases, including multiple sclerosis (MS), rheumatoid arthritis (RA), inflammatory bowel disease (IBD), asthma, and inflammatory skin disease among others.

#### 1. Multiple Sclerosis

Numerous genome-wide associated studies suggest that polymorphisms in the 5p13.1 regulatory region near *PTGER4* (encoding human EP4) are significantly associated with *PTGER4* gene expression and the susceptibility to MS (De Jager et al., 2009; Matesanz et al., 2012). The level of PGE_2_ was increased in cerebrospinal fluid of MS patients (Bolton et al., 1984). Compared with healthy individuals, Th17 cells from MS patients have higher levels of EP2 receptor, resulting in increased expression of proinflammatory cytokines like IFN-*γ* and GM-CSF and pathogenicity of Th17 cells (Kofler et al., 2014). Administration of EP2-receptor agonist did not affect proinflammatory cytokine production from Th17 cells of healthy individuals but increased IFN-*γ* and cerebrospinal fluid (CSF) 2 production from Th17 cells isolated from MS patients (Kofler et al., 2014). Studies using an animal model of MS [experimental autoimmune encephalomyelitis (EAE)] demonstrated that EP4-receptor gene deletion or pharmacological blockade of EP4 receptor during the immunization stage prevented EAE development in mice and downregulated Th1/Th17 cells (Yao et al., 2009; Esaki et al., 2010). Administration of an EP4-receptor agonist after peak disease response still reduces the peak EAE severity. These results thus suggest distinct roles of the EP4 receptor at different stages of EAE disease.

#### 2. Rheumatoid Arthritis

In the animal model of carrageenan-induced paw inflammation, neutralization of PGE_2_ by a monoclonal antibody prevented the development of tissue edema and hyperalgesia in affected paws, an effect associated with reduced IL-6 production (Portanova et al., 1996). Blockade of PGE_2_-EP2/EP4 signaling using receptor antagonists similarly suppressed joint inflammation in the mouse model of allergen-induced arthritis, which again was related to a reduction in IL-6 (McCoy et al., 2002; Honda et al., 2006). IL-6 is the key cytokine that mediates Th17 cell development, and PGE_2_ facilitates Th17 immune responses. Misoprostol, a PGE_2_ analog binding to EP2, EP3, and EP4 receptors, exacerbated collagen-induced arthritis in mice through activating the inflammatory IL-23/IL-17 axis, whereas an EP4-receptor antagonist reduced arthritis in a mouse model (Sheibanie et al., 2007a; Chen et al., 2010). This supports previous observations in genetically modifed mice, in which deletion of inducible mPGES-1 and EP4, but not EP3 or EP2, reduced arthritic incidence and severity in simlar experimental models (McCoy et al., 2002; Trebino et al., 2003). The therapeutic perspectives associated with EP4-receptor blockade will be developed in Section *X. D. Arthritis*.

#### 3. Inflammatory Bowel Disease and Colon Cancer

COX-2 activity in the colonic epithelial cells of IBD patients (Singer et al., 1998) and PGE_2_ levels in the lesions of IBD patients are elevated (Schmidt et al., 1996). Similarly, gene polymorphisms in *PTGER4* loci are associated with increased *PTGER4* gene expression and susceptibility to Crohn disease, suggesting a critical role of EP4 receptors (Libioulle et al., 2007; Glas et al., 2012). PGE_2_ is known to act in different ways in the gastrointestinal tract. For example, PGE_2_ plays fundamental roles in maintaining the gastrointestinal epithelial barrier, and therefore, blockade of PGE_2_ synthesis or the EP4 receptor was found to promote acute gastrointestinal injury in mice (Kabashima et al., 2002; Duffin et al., 2016) and induce gut damage in humans. However, misoprostol, an EP2/EP3/EP4 agonist, aggravated intestinal inflammation induced by 2,4,6-trinitrobenzene sulfonic acid in mice through promoting the inflammatory IL-23/IL-17 pathway (Sheibanie et al., 2007b). In contrast, genetic deletion of the PGE_2_ synthase mPGES-1 or the EP4 receptor in T cells ameliorated T cell–mediated chronic intestinal inflammation in mice, which was associated with reduction of the development of inflammatory Th1 and Th17 cells in the intestine (Maseda et al., 2018). Therefore, PGE_2_ may also facilitate T cell–mediated chronic intestinal inflammation in both mice and humans.

PGE_2_ has long been known to be associated with the development and progression of colorectal cancer (CRC), and use of COX inhibitors has been suggested to prevent CRC (Chan et al., 2005). Genome-wide association studies have indicated that polymorphisms in the NAD+-dependent 15-hydroxyprostaglandin dehydrogenase (15-PGDH), an enzyme that breaks down PGE_2_ into biologically inactive 15-keto-PGE_2_, are associated with higher risk for CRC, whereas polymorphisms in *PTGER2* were associated with lower CRC risk (Hoeft et al., 2010). PGE_2_ promoted human LoVo colon cancer cell proliferation and migration through activation of PI3K/Akt and glycogen synthase kinase 3*β*/*β*-catenin pathways; this could be prevented by the nonselective EP2- or EP4-receptor antagonist, AH6809 (EP1/2 and DP1 antagonist) or AH23848 (EP4/TP antagonist), respectively (Hsu et al., 2017). PGE_2_ and the EP2-receptor agonist butaprost promoted the survival of human colorectal carcinoma-15 cells, another human colon cancer cell line, and this was also prevented by AH6809 (Shehzad et al., 2014). In mice, deficiency of the EP2 receptor or treatment with the EP2-receptor antagonist, PF-04418948, reduced azoxymethane and dextran sodium sulfate–induced colon tumorigenesis associated with downregulation of proinflammatory genes (e.g., TNF-*α*, IL-6, CXCL1, and COX-2) in tumor-associated fibroblasts and neutrophils (Ma et al., 2015). Thus, further studies are needed to understand the differential roles of PGE_2_ in gastrointestinal homeostasis, such as, for example, the underlying mechanisms of how PGE_2_ protects against acute gastrointestinal injury and promotes mucosal regeneration but also promotes chronic (especially T cell–mediated) intestinal inflammation and colorectal cancer.

#### 4. Lung Inflammation

PGE_2_ and PGI_2_ are generally bronchoprotective, although these two prostanoids can increase inflammatory Th1 and Th17 cell numbers and function under autoimmune inflammatory conditions in other organs. In patients with aspirin-exacerbated respiratory disease, the PGE_2_-EP2 pathway is downregulated, but is associated with upregulation of PGD_2_ and leukotrienes as well as overactivation of type 2 innate lymphoid cells (Rusznak and Peebles, 2019). Using immunohistochemistry on human bronchial biopsy, it was reported that patients with aspirin-sensitive asthma had increased bronchial mucosal neutrophil and eosinophil numbers but reduced percentages of T cells, macrophages, mast cells, and neutrophils expressing EP2 (Corrigan et al., 2012). Given that EP2-receptor agonists were the only prostanoid-EP agonists to inhibit cytokine production in peripheral blood mononuclear cells, the authors concluded that EP2 agonists might be beneficial in patients with asthma. Likewise, in mice, the PGE_2_-EP2 signaling suppressed allergen sensitization and thus attenuated the development of Th2-polarized immunity and airway inflammatory responses (Zasłona et al., 2014). On the other hand, EP2-deficiency had the opposite effect and enhanced type 2 eosinophilic responses and IgE production in ovalbumin (OVA)-sensitized mice, whereas administration of misoprostol (EP2/EP3/EP4 agonist) to WT rather than EP2-deficient mice suppressed this inflammatory response and attenuated the IgE production (Zasłona et al., 2014). Results of this study provide evidence for a critical role of EP2 receptors in inhibiting airway inflammation through the dampening down of the Th2-cell cytokine surge. Such a view is not supported by Church and colleagues (2012), who reported that mPGES1-mediated PGE_2_ production in the lung contributed to the enhancement of allergic responses at the effector phase after allergen challenge. Based on parallel studies in congenic COX-1/2–deficient mice, they concluded that the primary prostanoid that was protecting against allergic inflammation was PGI_2_ and not PGE_2_. This is consistent with much earlier studies reporting that IP receptor–deficient mice have more severe allergic reactions in the lung compared with WT mice (Takahashi et al., 2002) and with more recent studies, in which deletion of mPGES-1 increased vascular production of PGI_2_ presumed to be from the redistribution of precursor PG substrate (Avendaño et al., 2018). Evidence supporting this notion that PGE_2_ might actually drive allergic inflammation comes from Gao and colleagues (2016), who found that PGE_2_-EP2 signaling in B cells actually promoted IgE production in OVA-induced asthma models. In trying to reconcile these conflicting results, it is important to note that genetic drift of mouse colonies almost certainly exists in different laboratories. This may in turn affect the nature of the allergic and inflammatory response action, including the amount of IgE and the cytokine profile generated with different immunization (e.g., the length of challenge) and experimental protocols (see Church et al., 2012; Gao et al., 2016 for further discussion).

Recently, PGE_2_-EP4/EP2 signaling has been reported to inhibit mouse as well as human ILC2 cell activation, which may contribute to control of allergic lung inflammation (Maric et al., 2018; Zhou et al., 2018b). By using agonists and antagonists in mouse models, EP2/EP4 receptors were reported to abrogate acute lung injury and inflammation through actions on various immune cells, including T cells, macrophages, B cells, eosinophils, innate lymphoid cells, and endothelial cells (Sheller et al., 2000; Sturm et al., 2008; Birrell et al., 2015; Konya et al., 2015; Draijer et al., 2016; Felton et al., 2018). Cicaprost, a potent and relatively selective IP agonist, was shown to inhibit IL-33–induced allergic lung inflammation through suppression of Th2 and ILC2 responses (Zhou et al., 2016, 2018a; Jian et al., 2017). Similarly, administration of ONO-1301, a novel prostacyclin analog with TxA_2_ synthase inhibitory activity, protected against OVA- and house dust mite–induced airway inflammation and remodeling in mice (Yamabayashi et al., 2012; Kimura et al., 2013).

These effects of PGE_2_ on lymphocytes are relevant for lung diseases, such as hypersensitivity pneumonitis, sarcoidosis, pulmonary fibrosis, and bronchial asthma. In mouse models of asthma, the activation of the EP3 receptor on bronchial epithelial cells inhibited the allergen-induced expression of chemokines (Hirata and Narumiya, 2012). EP2-receptor agonists inhibited GM-CSF release. EP4 receptors in human macrophages inhibited proinflammatory cytokines (IL-8) release (Gill et al., 2016), whereas EP4-receptor agonists inhibited neutrophils infiltration in the mice lung (Konya et al., 2015). In knockout mice, the EP4 receptor was involved in eosinophil and neutrophil infiltration in in vivo animal models of asthma, chronic obstructive pulmonary disease (COPD), and inflammation (Birrell et al., 2015). In human lung-transplant recipients, genetic variation in PGES and EP4 coding genes have been found to be associated with primary graft dysfunction and decreased Treg suppressor cell function (Diamond et al., 2014). It is imperative to further investigate why PGE_2_ acts mainly as a suppressant in lung inflammation, whereas it can exert both protective and proinflammatory activities in most other organ systems. With respect to manipulating PGE_2_ levels experimentally, it is important to note that EP4 (and EP3) will be activated at 10-fold lower concentrations (of PGE_2_) than EP2, whereas at high concentrations (≥100 nM), the PGF_2*α*_ (FP) receptor will be significantly activated as well as, in humans, the DP1 receptor (Clapp and Gurung, 2015). Taken together, this is likely to make the interpretation of the role of PGE_2_ in regulating inflammation complex potentially hard to extrapolate between different studies.

#### 5. Skin Inflammation and Cancer

PGE_2_ synthases and EP receptors are expressed in both human and mouse skin. Multiple types of cells within the skin, such as mast cells, macrophages, dendritic cells, and keratinocytes, can all produce PGE_2_. In a mouse model of delayed-type hypersensitivity, PGE_2_ and its receptor agonists suppressed skin inflammation by increasing the production of immunosuppressive type 2 cytokines (e.g., IL-4 or IL-10) (Shreedhar et al., 1998; Miyauchi-Hashimoto et al., 2001). Blockade of endogenous PGE_2_ production by a COX inhibitor or EP2 signaling by a relevant antagonist enhanced the production of the cytokine thymic stromal lymphopoietin from keratinocytes, whereas type 2 immune responses in the skin were attenuated by administering an EP2-receptor agonist (Sawada et al., 2019). Later on, PGE_2_ was shown to promote contact hypersensitivity, whereas EP4-receptor antagonist or EP4 deficiency reduced hapten-induced skin inflammation, probably through the action of EP4 receptors on skin dendritic-cell migration and Th1/Th17 cell expansion (Kabashima et al., 2003; Yao et al., 2009, 2013). Administration of a selective EP1-receptor antagonist ONO-8713 during the sensitization stage also suppresses hapten-induced skin inflammation (Nagamachi et al., 2007). Furthermore, Lee and colleagues (2019) suggested that blockade of EP2 and EP4 signaling (using genetically modified animals and receptor antagonists) reduced the generation of pathogenic Th17 cells and psoriatic skin inflammation. Blockade of the PGE_2_-EP4 pathway restricted allergic contact dermatitis in mice associated with reduced IL-22 production from T cells (Robb et al., 2018). In human inflamed skin (both atopic and psoriatic), the levels of PGE_2_ and gene expression of its synthases and receptors were found to be increased, and effective therapies downregulated PGE_2_ pathway–related gene expression (Fogh et al., 1989; Robb et al., 2018; Lee et al., 2019). In human keratinocytes, PGE_2_ suppressed CCL7 expression through EP2 and EP3 receptors, leading to a reduction of inflammatory T-cell homing within the skin (Kanda et al., 2004). Thus, PGE_2_ seems to have differential and partially opposing effects on different types of cells in human skin.

Development of skin tumor has long been known to be associated with enhanced COX-2–PGE_2_-EP signaling (Rundhaug et al., 2011). Deficiency of mPGES-1, the key enzyme mediating PGE_2_ synthesis, prevented B16 melanoma cell growth in vivo, and treatment with an EP4-receptor antagonist similarly inhibited not only the growth of B16 tumor cells but metastasis to bone marrow in mice (Inada et al., 2015). Oral, uveal, and cutaneous melanoma cells isolated from patients showed reduced IL-8 production in vitro, which was mimicked by the (EP3 > EP1) receptor agonist sulprostone and prevented by the EP3-receptor antagonist L-798106 (Venza et al., 2018).

Moving forward, a comprehensive understanding of the actions for PGE_2_ and its receptors on distinct cell types during skin inflammation will be particularly important.

## III. Cardiovascular System

### A. Healthy Condition

#### 1. Vascular Tone Regulation

The characterization of prostanoid receptors in human blood vessels is particularly important, and these results could provide therapeutic approaches for different diseases. Because of the fact that obtaining fresh human blood vessels on a regular basis is tough, many studies have instead focused on vessels obtained from different experimental animal models. However, several differences occur between vessels derived from human and rodents in terms of the responsible EP-receptor subtype for vasoconstriction/vasorelaxation induced by PGE_2_. For example, the control of vascular tone by PGE_2_ in human renal artery is regulated somewhat differently than rodent renal artery. For example, in rat renal artery, 11-deoxy-PGE1 (EP2 and EP4-receptor agonist) induced relaxation, whereas butaprost (EP2-receptor agonist) is relatively ineffective (Tang et al., 2000). The contraction induced by higher concentrations of PGE_2_ is mimicked by sulprostone, an (EP3 > EP1) receptor agonist (Tang et al., 2000). Similarly, in vivo renal blood flow studies in rats indicated that sulprostone caused transient renal vasoconstriction, whereas prolonged relaxation was obtained with EP4-receptor activation (Purdy and Arendshorst, 2000). In mice, the deletion of individual EP receptors demonstrated that EP2 receptor is partly involved in renal vasodilatation, whereas EP1 and EP3 receptors are involved in renal vasoconstriction (Imig et al., 2002). On the other hand, in human renal artery, no role for the EP2, EP1, and EP3 receptors was detected. Instead, the PGE_2_-induced relaxation was mimicked by CAY10598 (EP4 agonist), and the PGE_2_-induced contraction was blocked by the TP-receptor antagonist, S18886 (Eskildsen et al., 2014). Moreover, some studies have demonstrated that the pharmacology and mechanism underlying the effect of IP-receptor activation on vascular tone are different between human and rodent blood vessels (Clapp and Gurung, 2015).

Another difference between human and experimental animals has been observed in the regulation of pulmonary artery vascular tone by PGE_2_. Studies performed in human pulmonary artery demonstrated that sulprostone (EP3 > EP1 agonist) contraction is insensitive to TP-receptor antagonists (EP169 and GR32191) (Qian et al., 1994). This finding suggests the involvement of EP3 receptors in PGE_2_-induced contraction in human pulmonary artery. However, PGE_2_-induced relaxation occurs via EP2 or EP4 receptors in rabbit pulmonary artery contracted by norepinephrine (Kitamura et al., 1976), unlike in proximal human pulmonary artery, in which PGE_2_ fails to cause relaxation of arteries preconstricted with norepinephrine (Walch et al., 1999).

On the other hand, in some vessels similarities do exist between humans and experimental animals in terms of the role of PGE_2_ in regulating vascular tone. Studies report that PGE_2_-induced vasodilatation occurs via the EP4 receptor in saphenous vein derived from many species, including from human, rabbit, piglet, or guinea-pig tissue (Coleman et al., 1994; Lydford et al., 1996; Jones and Chan, 2005; Wilson and Giles, 2005; Foudi et al., 2011). It is important to note that pharmacological tools for the characterization of EP-receptor subtypes should be chosen carefully since they can have different effects on human and rodents. For example, GW627368X is frequently used as a selective EP4-receptor antagonist, but it can also bind to TP receptors in humans, whereas this is not the case in other species (Wilson et al., 2006).

Several studies demonstrated that PGE_2_-induced contraction is mostly mediated by EP3 receptor in human “healthy” vessels, such as coronary, internal mammary, intercostal, and pulmonary arteries (Foudi et al., 2011; Kozłowska et al., 2012; Longrois et al., 2012; Ozen et al., 2015). By contrast, the EP1 receptor is involved in the contraction evoked by PGE_2_ in several rodent vessels, including in rat renal artery, aorta, or mesenteric artery (Michel et al., 2007; Xavier et al., 2009; Silveira et al., 2014). On the other hand, the relaxant effect of PGE_2_ in the majority of human vessels is mediated by the EP4 receptor, whereas in rodents, vasodilatation induced by EP2-receptor activation is also observed (Imig et al., 2002; Davis et al., 2004; Foudi et al., 2008, 2011; Maubach et al., 2009). In accordance with this finding, PGE_2_ produces substantial hypertension in EP2 null mice (Kennedy et al., 1999). Somewhat variable effects of gene deletion on systolic blood pressure were reported, although mice in both studies developed profound salt-sensitive hypertension but not in controls (Kennedy et al., 1999; Tilley et al., 1999). Taken together, this suggests a major role for EP2 receptors in regulating vascular tone and sodium handling in the kidney, at least in mice.

PGI_2_ induces vasodilatation by the activation of IP receptor. The effects of PGI_2_ analogs on vascular tone have mostly been determined in in vitro studies using pulmonary arteries. PGI_2_ analogs, such as treprostinil, iloprost, and beraprost induced relaxation in both human and rat pulmonary arteries (Benyahia et al., 2013, 2015; Shen et al., 2019). However, when the precontractile agent is endothelin-1, iloprost and treprostinil are able to relax human pulmonary artery but not rat pulmonary artery (Benyahia et al., 2015). On the other hand, TP-receptor activation by high doses of PGI_2_ elicits contraction in some rodent vessels, although such an effect is not exhibited in human vessels (Xavier et al., 2009; Baretella and Vanhoutte, 2016). Relaxation by prostacyclin analogs in normal vascular preparations is consistently enhanced over a wide agonist concentration range through blocking EP3-receptor function or G_i_ coupling and suggests tonic activation of these receptors opposes the action of PGI_2_ and its analogs in the cardiovascular system (Clapp et al., 2020). Overall, characterization of EP-receptor subtypes and effects of IP/EP-receptor agonist/antagonist on vascular tone are still not determined in several human vessels, such as coronary artery, carotid artery, or aorta. Further studies are warranted and will provide therapeutic approaches for different diseases, such as atherosclerosis or aneurysm.

### B. Cardiovascular Diseases

#### 1. Hypertension

The substantial roles of prostanoids in the regulation of blood pressure are highlighted by the prohypertensive effects of nonsteroidal anti-inflammatory drugs (NSAIDs) and COX-2 inhibitors (Snowden and Nelson, 2011; Ruschitzka et al., 2017). Likewise, when the PGIS gene is deleted, mice become hypertensive and show fibrosis and vascular remodeling in the kidney (Yokoyama et al., 2002) but not when the IP receptor is deleted (Hoshikawa et al., 2001), suggesting additional targets for PGI_2_ in the regulation of vascular function, most likely through PPARs (Clapp and Gurung, 2015). Elevated expressions of mPGES-1 and COX-2 are found in hypertensive patients, mice, and rats versus their normotensive controls (Boshra et al., 2011; Avendaño et al., 2016). Furthermore, deletion of the mPGES-1 gene in hypertensive mice prevented the increased vasoconstrictor response to angiotensin 2 (Ang-II) (Avendaño et al., 2018). In accordance with this finding, an mPGES-1 inhibitor decreased the contractile response to noradrenaline in human arteries and veins (Ozen et al., 2017). Overall, both in human and rodents, the enzymes responsible for the synthesis of PGI_2_ and PGE_2_ are involved in the pathogenesis of arterial hypertension.

Under physiologic conditions, there is a balance between the effects of EP1/EP3 receptor (vasoconstriction) and those of EP2/EP4 receptor (vasodilation), whereas in hypertensive models and patients with cardiovascular disease, this balance is likely to be disrupted (Clapp and Gurung, 2015). Consistent with this notion, EP1 receptor participated in impaired vascular function observed in hypertensive animal models (Avendaño et al., 2016). Moreover, in mice, a reduced systolic blood pressure was observed upon treatment with various EP1-receptor antagonists (ONO-8713, AH6809, SC51322) and with deletion of the EP1 gene (Stock et al., 2001; Guan et al., 2007; Rutkai et al., 2009). EP3-receptor agonists, such as sulprostone (EP3 > EP1 agonist), MB28767, or SC46275 increased mean arterial pressure in wild-type mice (Zhang et al., 2000), and increased renal blood flow was observed in EP3-deficient mice (Audoly et al., 2001). Furthermore, EP2- and EP4-receptor knockout mice have elevated systolic blood pressure (Kennedy et al., 1999; Tilley et al., 1999; Xu et al., 2019). The role of EP2 in the regulation of blood pressure in mice is supported by the study, which revealed an association between a polymorphism of the EP2 gene and essential hypertension in men (Sato et al., 2007). In contrast, mostly EP3 agonists, such as sulprostone and misoprostol, which are used as clinical practice, are associated with an increased incidence of ischemic stroke or myocardial infarction in patients probably due to coronary constriction. Globally, these results suggest a strong role for the EP3 receptor in the control of arterial blood pressure (Guerci et al., 2013; Vital et al., 2013; Masclee et al., 2018; Mazhar et al., 2018; Schink et al., 2018). These in vivo observations are supported by the numerous in vitro data on the vasoconstrictor role of the EP3 receptor in human vasculature as mentioned previously (Qian et al., 1994; Norel et al., 2004a; Foudi et al., 2011; Longrois et al., 2012; Ozen et al., 2015).

The expression of PGIS and the concentrations of the stable PGI_2_ metabolite (6-keto-PGF_1*α*_ or 2,3-dinor-6-keto-PGF_1*α*_) measured in hypertensive patients were found to be similar or lower than those obtained for normotensive patients (Lemne et al., 1992; Klockenbusch et al., 2000; Vainio et al., 2004; Hellsten et al., 2012). A number of polymorphisms in the PGIS gene have been described that are not associated with essential hypertension in humans (Nakayama et al., 2002a, 2003). However, several studies performed in hypertensive animal models, including spontaneously hypertensive rats, Dahl salt-sensitive rats, deoxycorticosterone-salt hypertensive rats, and renovascular hypertensive rats, demonstrate increased levels of 6-keto-PGF_1*α*_, probably via the induction of COX-2 expression (Ishimitsu et al., 1991; Matsumoto et al., 2016). Infusion of PGI_2_ or its mimetics exhibited similar results in humans and rats and caused reductions of blood pressure (Pickles and O’Grady, 1982; Frölich, 1990; Kato et al., 1992; Zlatnik et al., 1999; Picken et al., 2019). Finally, polymorphisms of the PGIS promoter have been discovered and are associated with increased synthesis of PGIS (Stearman et al., 2014).

The potent vasodilatory effects of PGI_2_ in the systemic and pulmonary circulation are well documented (Clapp and Gurung, 2015). The evidence that IP receptors per se regulate blood pressure under physiologic conditions is not supported by gene-deletion studies, in which mice are normotensive (Hoshikawa et al., 2001). However, loss of the IP receptors leads to the development of renovascular hypertension, and mice have an exaggerated hypertensive and remodeling effect of hypoxia in the lung. Thus in disease, the IP receptor appears to become dysfunctional in the vascular system and, in doing so, may unmask the contractile effects of PGI_2_ as observed in spontaneously hypertensive rats (Félétou et al., 2009). Not only in hypertensive rodent models but also in diabetic and aged rats, IP-receptor signaling appears to be impaired. PGI_2_ no longer caused vasodilatation but became a prominent endothelium-derived vasoconstrictor by activating TP receptors (Vanhoutte, 2011). However, in most human vascular preparations, PGI_2_ or its analogs induced only relaxation (Benyahia et al., 2015; Foudi et al., 2017).

Data from clinical trials in pulmonary hypertensive (PH) patients showed that chronic treatment with PGI_2_ analogs leads to a significant fall in pulmonary arterial pressure and pulmonary vascular resistance but does not lead to a drop in systemic blood pressure with standard doses (Picken et al., 2019). This may point to a “relative” pulmonary-selective effect of these agents, although common side effects of all IP agonists are headache and flushing, suggesting a potent vasodilatory effect on cerebral and skin blood vessels, respectively. The situation may be different in patients with systemic hypertension, although the effects of IP agonists on blood systemic arterial pressure in this condition have not been routinely studied.

#### 2. Diabetes

The plasma concentrations of PGE_2_ in diabetic patients were found to be similar or higher than those obtained for nondiabetic patients (Arisaka et al., 1986; Axelrod et al., 1986; Mourits-Andersen et al., 1986). In diabetic rats, the plasma levels of PGE_2_ were also increased (Axelrod and Cornelius, 1984; Craven et al., 1987).

Studies performed in diabetic mice and rats demonstrated that increased vascular tone and diabetic nephropathy were reversed by either AH6809 or ONO-8713 (EP1-receptor antagonists). EP1-receptor overexpression was detected in diabetic rat glomeruli by using Northern blot analysis, and confirmation was obtained by using in situ hybridization. This observation suggests that EP1 receptors could contribute to the development of hypertension and nephropathy in diabetic rodents (Makino et al., 2002; Rutkai et al., 2009); however, the contribution of EP1 receptor in diabetic patients needs to be evaluated. The EP2 receptor could also be involved in diabetic retinopathy in both humans and rats, possibly because of accelerated retinal vascular leakage, leukostasis, and endothelial cell apoptosis (Wang et al., 2019). On the other hand, other studies performed on streptozotocin-induced diabetic rats showed that EP2 and EP4 receptors were involved in the protective roles of PGE_2_ (Vennemann et al., 2012; Yasui et al., 2015), highlighting the complex role of these prostanoid receptors in different experimental models. Moreover, the suppression of EP4 receptor–associated protein has been suggested as a novel strategy for the treatment of diabetes in mice (Vallerie et al., 2016; Higuchi et al., 2019).

In both diabetic patients and mice models, upregulation of the EP3 receptor in pancreatic islet of Langerhans has been reported by using real-time PCR (Kimple et al., 2013; Amior et al., 2019). Blockade of the EP3 receptor by DG-041 (Su et al., 2008) in combination with activation of EP4 receptor increased *β*-cell proliferation in humans but not in mice (Carboneau et al., 2017). Moreover, DG-041 had no significant effects on the diabetic phenotype of mice (Ceddia et al., 2019), whereas L-798106 (EP3-receptor antagonist) has been shown to decrease insulin resistance in db/db mice (Chan et al., 2016). In contrast with this finding, genetic deletion of all three EP3 isoforms or deletion of only EP3*α* and EP3*γ* isoforms resulted in increased insulin resistance in mice when they were fed a high-fat diet (Ceddia et al., 2016; Xu et al., 2016).

The release of 6-keto-PGF_1*α*_ was significantly decreased in both diabetic patients and rats (Lubawy and Valentovic, 1982; Jeremy et al., 1987a; Brunkwall and Bergqvist, 1992; Kalogeropoulou et al., 2002; Bolego et al., 2006; Peredo et al., 2006), suggesting loss of circulating PGI_2_. The expression of PGIS was lower in subcutaneous arteries from diabetic patients, whereas IP-receptor expression remained unchanged (Mokhtar et al., 2016b; Safiah Mokhtar et al., 2013). In contrast, another study performed on human platelet showed that IP-receptor expression was inversely correlated with HbA1c levels (Knebel et al., 2015), which may contribute to platelet hyperactivity in humans with type 2 diabetes. This is certainly consistent with the heightened thrombotic state observed in mice not expressing the IP receptor (Hoshikawa et al., 2001). Furthermore, PGIS- and IP-receptor expressions were decreased in Zucker diabetic fatty rats and streptozotocin-induced diabetic rats, respectively (Nasrallah and Hebert, 2004; Lu et al., 2005). Even though the expression of the IP receptor was not changed in diabetic patients (Safiah Mokhtar et al., 2013), PGI_2_-induced relaxation was increased in coronary arterioles of patients with diabetes, presumably to compensate for decreased nitric oxide bioavailability (Szerafin et al., 2006). In contrast, there was no compensatory role of PGI_2_ in streptozotocin-induced diabetic rats (Mokhtar et al., 2016a), whereas PGI_2_-induced contraction was increased, and the relaxation induced by IP-receptor agonist was decreased in diabetic mice (Kimura et al., 1989; Przygodzki et al., 2015).

#### 3. Abdominal Aortic Aneurysm

Dilatation and weakening of the aorta in abdominal aortic aneurysms (AAAs) are accompanied by an alteration in the blood vessel, such as an increase of local inflammation, smooth muscle cell apoptosis, elevated oxidation stress, and especially extracellular matrix degradation via matrix metalloproteinase (MMP) activity (Han et al., 2018). Several research groups (including our own) have demonstrated that PGE_2_ release and mPGES-1 expression were increased in vascular preparations derived from AAA patients (Camacho et al., 2013; Solà-Villà et al., 2015; Gomez et al., 2016). These in vitro results are supported by clinical studies, which demonstrated a lower aneurysm growth rate in patients receiving NSAIDs (Walton et al., 1999). In line with human studies, Ang-II–induced AAA formation in mice resulted in increased PGE_2_ levels and deletion of mPGES-1 protected against AAA formation (King et al., 2006; Wang et al., 2008).

The role of EP-receptor subtypes has also been investigated in the pathogenesis of AAA. In human AAA samples, higher EP4-receptor expression versus normal samples was demonstrated by real-time PCR studies (Camacho et al., 2013). Exposure of aortic smooth muscle cells (SMCs) and macrophages derived from human AAA preparations to EP4-receptor antagonists (CJ-42794 or ONO-AE3-208) decreased MMP activation and proinflammatory cytokines secretion (Cao et al., 2012; Yokoyama et al., 2012; Mamun et al., 2018). These findings suggested that EP4-receptor antagonists could be a therapeutic target for the treatment of AAA. Similarly, administration of EP4-receptor antagonist or deletion of the EP4 receptor in ApoE knockout mice reduced AAA formation via diminution of cytokine/chemokine levels and MMP activities (Cao et al., 2012; Yokoyama et al., 2012; Mamun et al., 2018). However, there is one contradictory study performed in hyperlipidemic mice. In this study, deficiency of EP4 receptor increased AAA formation induced by Ang-II (Tang et al., 2011b). As described above (in section *II. A. 4. Macrophages*), it has recently been shown that deletion or antagonism (PF-04418948) of the EP2 receptor is responsible for reduced macrophage infiltration and intracranial aneurysm formation in rodents (Aoki et al., 2017a,b; Shimizu et al., 2019). Taken together, these results suggest that aneurysm development involves SMC and macrophage crosstalk with EP2 and/or EP4-receptor activation, in human and rodent models. As a hypothesis, the EP receptors involved could be dependent on the type of aneurysm (aortic or intracranial artery). Overall, most studies have addressed the role of the EP4 receptor in human AAA development, and the roles of EP2 and EP4 receptors were investigated in rodent models. EP3-receptor subtypes were also detected in aortic SMCs derived from patients with or without AAA (Bayston et al., 2003), and some EP3 mRNA splice variants were differentially expressed between human aortic SMC derived from control versus AAA patients. Further investigations may shed light on a potential novel role of EP3 receptors in arterial aneurysms.

In contrast, only a few studies investigated the role of PGI_2_ and its receptor in the pathogenesis of AAA. Solà-Villà and colleagues (2015) demonstrated that there were increased PGE_2_ levels in human AAA samples, which were significantly correlated with enhanced levels of PGI_2_ released from the tissue samples, whereas in other studies there was no change in PGI_2_ levels from human AAA samples obtained from patients with Marfan syndrome (Soto et al., 2018). However, Wang et al. (2008) demonstrated that deletion of mPGES-1 gene increased 2,3-dinor-6-keto-PGF_1*α*_ concentrations in urine of mice with Ang-II–induced AAA. Although this suggested that PGI_2_ has a protective effect against AAA formation in mice, further studies in both human and rodents need to be conducted to substantiate the role and mechanism of PGI_2_ in AAA development.

#### 4. Obesity

Obesity is defined as a low-grade inflammatory disease and associated with many cardiovascular diseases, including diabetes, hypertension, and metabolic syndrome (Ceddia et al., 2016). Since PGE_2_ and PGI_2_ are induced under inflammatory conditions, several studies have focused on the role of these mediators in the development of obesity.

Recently, we and other groups have demonstrated that PGE_2_ levels in plasma and omental adipose tissue are greater in obese patients (García-Alonso et al., 2016; Ozen et al., 2019), whereas there are conflicting results in terms of the PGE_2_ levels measured in obese rodent models (Pham Huu Chanh et al., 1987; Cunha et al., 2010; Rocha-Rodrigues et al., 2017). Another difference has been in the detected level of mPGES-1 expression between obese patients and obese rodents: mPGES-1 expression remained unchanged in the adipose tissue of obese patients, whereas it is decreased in those obtained for obese mice (Hétu and Riendeau, 2007; García-Alonso et al., 2016). EP3 mRNA expression is significantly and consistently upregulated in primary adipocytes isolated from high-fat diet–induced obese rats and human subjects (Chan et al., 2016). Moreover, EP3 mRNA levels are positively correlated with the body mass index in humans and TNF-*α* and MCP-1 levels in adipose tissue (Chan et al., 2016). On the other hand, downregulation of EP3 isoforms in high-fat diet–induced obese mice was reported by using both Western blot and real-time PCR techniques (Xu et al., 2016). In accordance with these findings, other studies indicated that EP3 knockout mice had an obese phenotype with abnormal lipid distribution and accumulation versus wild-type mice (Sanchez-Alavez et al., 2007; Ceddia et al., 2016). In contrast, short-term treatment DG-041, an EP3-receptor antagonist, had no effect on body composition and glycemic control in obese diabetic mice (Ceddia et al., 2019). Overall, the roles of mPGES-1 enzyme and EP3 receptor in obesity have been evaluated in many studies; however, there are several differences documented between human and rodents.

The roles of other EP-receptor subtypes have so far only been determined in obese animal models but not in obese patients, and this should be evaluated in future studies. The EP4-receptor agonist TCS 251 induced a greater relaxation in coronary arterioles derived from obese rats (Santiago et al., 2016), and this could be due to increased EP4-receptor expression in obesity. Enhanced EP4-receptor expression could have protective roles since the activation of EP4 receptor is involved in the reduction of adipose tissue inflammation (Tang et al., 2015; Yasui et al., 2015). On the other hand, only one study demonstrated that EP2 levels are significantly decreased in macrophages of obese diabetic mice (Hellmann et al., 2013); however, the role of EP2 receptor in the development of human obesity remains to be elucidated. Furthermore, it should be noted that in the Hellman study, only Western blot techniques were used for quantification of the EP receptor. Since antibodies for EP receptors are generally not very specific unless verified by small interfering RNAs, gene deletion, or expression systems, other techniques, such as in situ hybridization or PCR, are necessary for verification.

In humans, weight reduction induces a significant decrease of 6-keto-PGF_1*α*_ production in adipose tissue (Katz and Knittle, 1991). In contrast, in high-fat diet–induced obese rats, spontaneously hypertensive obese rats, or obese Zucker rats, there is no change, or there is a decrease in 6-keto-PGF_1*α*_ levels (Goodwill et al., 2008; Hodnett et al., 2009; Mendizábal et al., 2013; Vendrame et al., 2014; Lee et al., 2017; Lemaster et al., 2017). PGI_2_-mediated vasodilatation is impaired in patients with obesity and metabolic syndrome (Limberg et al., 2013). In accordance with this finding, in obese Zucker rats, a decrease of PGI_2_-induced vasodilatation is observed, and the contraction response induced by PGI_2_ via TP-receptor activation is increased (Xiang et al., 2006; Baretella and Vanhoutte, 2016). On the other hand, another study performed on these rats demonstrated that neither IP nor TP-receptor expression was changed by immunofluorescence studies (Hodnett et al., 2009). Overall, even though 6-keto-PGF_1*α*_ production is different between human and rodents in obesity, vasodilator effects of PGI_2_ were decreased in both obese patients and rodents. The mechanism underlying this decrease needs to be elucidated in obese patients; future investigations could thus provide novel therapeutic aspects especially for obesity-related cardiovascular diseases.

#### 5. Atherosclerosis

The levels of PGE_2_ are increased in preparations with atherosclerotic lesions and plasma from atherosclerotic patients and rodents (Rolland et al., 1984; Gómez-Hernández et al., 2006a; Pang et al., 2019). Elevated levels of mPGES-1 expression were observed in human atherosclerotic preparations (Gómez-Hernández et al., 2006a), and the deletion of mPGES-1 gene in mice retarded atherosclerosis and neointimal hyperplasia (Wang et al., 2006, 2011). This protection may in part be conferred by a compensatory increase in PGI_2_ levels, which is associated with deletion of mPGES-1 in mice. Using double-knockout mice (mPGES-1 and IP), protection from injury was lost, and neointima formation was more severe compared with IP-deficient mice, confirming a role for PGE_2_ as well as PGI_2_ in restraining the neointimal response to injury (Hao et al., 2018).

There are contradictory results for the involvement of EP1 receptor in the pathogenesis of atherosclerosis. One study demonstrated that EP1 receptor was not detected in human carotid plaques (Cipollone et al., 2005), whereas another study demonstrated the expression of EP1 receptors in the shoulder of plaques by Western blot, PCR, and immunohistochemical techiques (Gómez-Hernández et al., 2006a). In addition, patients treated with statins had decreased EP1-receptor expression in atherosclerotic plaques, which may be associated with the beneficial effects of statins (Gómez-Hernández et al., 2006b). EP2-receptor expression was detected in human carotid and femoral plaques, but there was no difference in EP2-receptor expression levels between atherosclerotic and nonatherosclerotic preparations, as determined by Western blot, PCR, and immunohistochemical studies (Gómez-Hernández et al., 2006a,b; Muto et al., 2010). Interestingly, some oxidized phospholipids that accumulate in atherosclerotic lesions have been found to activate EP2 receptors and might hence play a role in atherogenesis in humans (Li et al., 2006). EP2 receptors have also been implicated in a mouse atherosclerosis model, although with a different angle: vascular SMC from EP2 knockout mice showed increased migration and proliferation, suggesting that EP2-receptor activation could be beneficial for the treatment of vascular remodeling, as observed in atherosclerosis (Zhu et al., 2011).

There is a significant increase of EP3-receptor expression in human carotid plaques, which was detected by Western blot, PCR, and immunohistochemical studies (Gómez-Hernández et al., 2006a). On the other hand, another study revealed that oxidation of low-density lipoprotein decreased EP3-receptor expression in human macrophages. The downregulation of EP3 expression by oxidized low-density lipoprotein resulted in impairment of EP3-mediated anti-inflammatory effects (Sui et al., 2014). Hypercholesterolemia and increased diet-induced atherosclerosis were observed in mice with genetic deletion of hepatocyte-specific EP3 receptor (Yan et al., 2017). These studies suggest that EP3-receptor activation could have beneficial roles in the treatment of atherosclerosis and hypercholesterolemia. In contrast, the study performed in mice using different prostaglandin receptor antagonists and small interfering RNA revealed that EP3*α* and *β* splice variants are involved in neointimal formation in response to injury (Zhang et al., 2013a).

The overexpression of EP4 receptor was detected in human carotid atherosclerosis by Western blot, PCR, and immunohistochemical studies, and the EP4 receptor was shown to be involved in destabilization of the plaques by the regulation of MMPs (Cipollone et al., 2005; Gómez-Hernández et al., 2006a). Similarly, PGE_1_-OH, an EP4-receptor agonist, stimulated MMP-9 expression in macrophages of mice (Pavlovic et al., 2006). The deletion of the EP4 receptor in mice macrophages reduced aortic atherosclerosis (Babaev et al., 2008), whereas deletion of the EP4 receptor in bone marrow–derived cells in mice did not change atherosclerotic lesion size but increased inflammation (Tang et al., 2011a). A recent study demonstrated that hypercholesterolemia was observed in EP4 knockout mice, and treatment with EP4-receptor agonist (CAY10580) in mice fed with a high-fat diet prevented diet-induced hypercholesterolemia (Ying et al., 2018). Consistent with a role for EP4 receptors in re-endothelialization after angioplasty-wire injury, deletion of EP4 in endothelial cells was enhanced while EP4 agonists protected against neointimal formation (Hao et al., 2018). In humans, PGE_2_ decreased chemokine levels in human macrophage through EP4-receptor activation, and this could prevent atherosclerotic plaque development (Takayama et al., 2002, 2006). The role of EP4 receptor in atherosclerosis was studied in more detail in both humans and mice by using genetic deletion or selective receptor agonist/antagonists; however, results regarding the role of other EP-receptor subtypes are contradictory, and mechanisms of their beneficial or harmful effects are not fully understood yet.

The link between PGI_2_ signaling and atherosclerosis is highlighted by the side effects of COX-2 inhibitors via their inhibitory effect on PGI_2_ levels. Generally, PGI_2_ has been found to play athero-protective roles. At the time of the development of PGI_2_ for the treatment of pulmonary arterial hypertension (PAH), much of the focus of the clinical use of PGI_2_ was on the treatment of peripheral vascular diseases, such as critical limb ischemia associated with atherosclerosis and Buerger disease reviewed in Clapp and Gurung (2015). Moreover, PGI_2_ has an inhibitory effect on platelet-derived growth factor (PDGF) production, which plays an important role on SMC proliferation and neointimal formation in atherosclerosis. Both antiproliferative and also lipid-lowering effects of PGI_2_ were observed in aortic cells derived from both human and rodents and lead to antiatherosclerotic effects (Clapp and Gurung, 2015). The urinary levels of 2,3-dinor-6-keto-PGF_1*α*_ are greater in patients and mice with atherosclerosis and suggest that greater PGI_2_ production could act as a compensatory mechanism. In addition, it should be noted that the measurement of the urinary metabolite 2,3-dinor-6-keto-PGF_1*α*_ reflects kidney synthesis PGI_2_ and is not representative of total production in whole body. However, another study performed on patients with atherosclerotic diseases demonstrated that PGI_2_ levels are not associated with major adverse cardiovascular events and vascular inflammation (Wang et al., 2018).

IP-receptor expression was decreased in human atherosclerotic plaques (Di Taranto et al., 2012), and an IP-receptor mutation was associated with atherothrombosis in a patient cohort with a high risk of cardiovascular disease (Arehart et al., 2008). Similarly, the genetic deletion of IP receptor in mice or pharmacological inhibition of PGI_2_ by COX-2 inhibitors resulted into a significant acceleration in atherogenesis (Kobayashi et al., 2004; Gitlin and Loftin, 2009). In accordance with this finding, PGI_2_ analogs (octimibate and BMY 42393) reduced early atherosclerosis in hyperlipidemic hamster (Kowala et al., 1993).

#### 6. Cerebral Stroke

Two types of strokes are reported in literature: ischemic and hemorrhagic stroke (Mehndiratta et al., 2015). In a rat model of ischemia stroke, an upregulation of COX-2 mRNA is observed after the occlusion of the middle cerebral artery, and this leads to increased production of PGE_2_ by 292% ± 57% after 24 hours (Nogawa et al., 1997). Similarly, PGE_2_ production is increased after 24 hours of brain ischemia in mice, and it is associated to a significant increased level of COX-2 mRNA and proteins (Yokota et al., 2004). Similar results are found in humans, with increased mRNA and protein levels of COX-2 found in post-mortem infarcted human brain (Sairanen et al., 1998). The COX-2 inhibitor, NS-398, attenuated the elevation of PGE_2_ in the postischemic brain and reduced the volume of infarction (Nogawa et al., 1997). Another study demonstrated that deletion of mPGES-1–coding gene in mice abolished postischemic PGE_2_ production in the cortex, and that is associated with a reduction of myocardial infarction, edema, and cell death in comparison with WT mice (Ikeda-Matsuo et al., 2006). In rats, inhibition of autophagy after an ischemic stroke shows a significant decreased level of proinflammatory molecules, such as PGE_2_ (He et al., 2019). The important role of PGE_2_ is observed in adult human, wherein COX-2/PGE_2_ pathway is associated with the middle cerebral artery occlusion and the hemorrhagic stroke in patients with Moyamoya disease caused by blocked arteries at the base of the brain. Furthermore, COX-2 and mPGES-1 were found abundant in the vascular walls of middle cerebral artery and superficial temporal artery in patients with Moyamoya disease (Zhang et al., 2016a). In line with these results, it was found that the polymorphism of COX-2 (-765 G>C) in humans is linked to a decreased risk of stroke (Cipollone et al., 2004), highlighting a strong link between COX-2 and enhanced cardiovascular risk.

The genetic deletion of EP1 receptor is related to a neurotoxic effect in mouse model of brain transient ischemia (Zhen et al., 2012). Treatment with the EP1-receptor antagonist, SC51089 or EP1 gene deletion demonstrated an improvement of middle cerebral artery occlusion in mice (Kawano et al., 2006) and reduced neuronal death after an episode of transient forebrain ischemia (Shimamura et al., 2013). Moreover, EP1 knockout mice were associated with a decreased ischemic lesion after stroke and increased cerebral blood flow (Ahmad et al., 2006; Saleem et al., 2007). Another study showed that in an ischemic stroke model, pretreatment with a specific EP1-receptor antagonist, ONO-8713, reduced the size of the infarct (Ahmad et al., 2008). Finally, it was shown that inhibition of EP1 receptor improved the survival of hippocampal slices (in culture) from mice with ischemic stroke induced by oxygen-glucose deprivation (Zhou et al., 2008). These observations suggested that EP1-receptor antagonists could be therapeutic targets in ischemic stroke.

The EP2 receptor appears to have an important beneficial role in reducing cerebral ischemia in an experimental model of stroke (Andreasson, 2010). Consistent with this, genetic deletion of EP2 receptor resulted in increased infarct volumes in mice (McCullough et al., 2004; Liu et al., 2005). Moreover, pharmacological activation of EP2 by ONO-AE1-259-01 significantly reduced the infarct volume in mice (Ahmad et al., 2010). However, recent studies demonstrated that neuronal EP2-receptor expression is induced after cerebral ischemia (Liu et al., 2019). Validation of the anti-EP2 antibody used in that study was performed by using cerebellar lysates and HEK cells overexpressing the EP2 receptor. The blockade of EP2 in mice contributed to cerebro-protection by reducing neuroinflammation (Liu et al., 2019), and EP2 knockout mice were shown to be more protected from intracerebral hemorrhage strokes (Leclerc et al., 2015b).

EP3 receptor is the most abundant receptor in brain (Leclerc et al., 2016) and appears to play an important role in acute ischemic stroke. A study showed that activation of EP3 receptor with ONO-AE-248 increased infarct size in experimental stroke (Ahmad et al., 2007). In the same context, the genetic deletion of EP3 receptor in a model of cerebral ischemia resulted in a reduction in cell death and infarction volumes induced by oxygen and glucose deprivation (Saleem et al., 2009). Deletion of EP3 receptor in mice suppressed damage to the blood-brain barrier, activation of microglia, and neutrophil infiltration into the ischemic cortex (Ikeda-Matsuo et al., 2011). In intracerebral hemorrhage, the genetic suppression of EP3 resulted in a decrease in intracerebral hemorrhage–induced brain damage and improved functional recovery. In addition, EP3 knockout mice showed a significant reduction in astrogliosis, microglial activation, blood-brain barrier degradation, and neutrophil infiltration. Overall, these studies suggested a detrimental role of the PGE_2_-EP3–signaling axis in modulating brain injury, inflammation, and neurologic functional recovery (Leclerc et al., 2015a).

In a mouse model of cerebral ischemia, EP4 activation by ONO-AE1-329 reduced infarct volume, and deletion of EP4 exacerbated stroke injury (Liang et al., 2011). Similarly, treatment by an EP4 agonist, L-902,688, reduced infarct volume after ischemic stroke in mice and rats (Akram et al., 2013; DeMars et al., 2018). In humans, no difference of EP4-receptor expression was detected in blood of ischemic stroke patients versus asymptomatic patients by real-time PCR studies (Ferronato et al., 2011). Further studies are necessary to examine the roles of EP receptors and the potentially beneficial effects of agonists/antagonists in cerebral stroke.

The possible beneficial effect of PGI_2_ in stroke was explored many years ago in humans. Accordingly, treatment with PGI_2_ in patients diagnosed with ischemic stroke had a positive impact on stroke recovery with no neurologic deficit or minor residual hemiparesis (Gryglewski et al., 1983). Other clinical trials and studies in humans demonstrated that PGI_2_ infusion showed beneficial effects after a stroke (Hsu et al., 1987). Moreover, PGI_2_ agonists climprost (TTC-900) or TEI-7165 were described to have protective effects on postischemic neuronal damage in a gerbil model (Matsuda et al., 1997; Cui et al., 1999).

IP-receptor activation can attenuate anatomic and functional damage after ischemic stroke. The infarct volumes and neurologic deficit scores are significantly greater in IP knockout mice after both transient and permanent middle cerebral artery occlusion. Treatment with the IP-receptor agonists beraprost or MRE-269 before and after transient middle cerebral artery occlusion reduced the neurologic deficit score and infarct volume in WT mice (Saleem et al., 2010; Yang et al., 2017). Moreover, several studies performed on animals demonstrated beneficial roles of PGI_2_ in cerebral blood flow (Bentzer et al., 2003; Lundblad et al., 2008). However, administration of PGI_2_ did not change cerebral blood flow in human after subarachnoid hemorrhage (Rasmussen et al., 2015). On the other hand, in another study performed in patients with cerebral infarction, beraprost plus aspirin was found to be more effective than aspirin alone to reduce the recurrence of cerebral infarction or death (Chen et al., 2017). Moreover, an association between polymorphism of the IP-receptor gene and platelet activation was found in patients with cerebral infarction (Shimizu et al., 2013).

#### 7. Arrhythmia

The study performed on rabbits demonstrated that PGE_2_ prevented drug-induced torsade de pointes, which is life-threatening arrhythmia (Farkas and Coker, 2003). Antiarrhythmic effect of PGE_2_ was also demonstrated in humans (Mest and Rausch, 1983). In contrast, microinjection of PGE_2_ in rats or superfusion of the rat cardiac myocytes with PGE_2_ resulted in tachycardia (Li et al., 1997; Zaretskaia et al., 2003), whereas 41% of women receiving misoprostol, EP2/EP3/EP4 agonist, had late decelerations or bradycardias (Kolderup et al., 1999). EP3 receptors located presynaptically on sympathetic nerve fibers supplying the heart of pithed rats strongly inhibit the neurogenic tachycardia. Sulprostone (EP3 > EP1), but not the IP/EP1-receptor agonist iloprost, inhibited the increase in electrically provoked heart rate dose-dependently. L-826266 (EP3-receptor antagonist) has no effect on basal heart rate or diastolic blood pressure but reduces the inhibitory effect of sulprostone (Kozłowska et al., 2012).

PGI_2_ induced a marked reduction in the contraction rate of the rat cardiac myocytes and had a protective effect against the arrhythmias (Li et al., 1997). Similar effect was also observed in in vivo studies performed on rats and showed that low doses of PGI_2_ reduced arrhythmias induced by coronary artery ligation or aconitine (Mest and Förster, 1978; Johnston et al., 1983). However, in humans, PGI_2_ does not appear to have a cardiac antiarrhythmic effect and may increase the atrial and ventricular recurrent response. This effect could be related to an increase in adrenergic tone (Brembilla-Perrot et al., 1985). Increased excretion of 6-keto-PGF_1*α*_ has been observed in patients with ventricular arrhythmia (Chlewicka and Ignatowska-Switalska, 1992). There are very few studies regarding the role of IP and EP receptors in arrhythmia so far.

#### 8. Pulmonary Circulation and Hypertension

In non-PH large pulmonary vessels (>2-mm diameter), activation of PGE_2_ and PGI_2_ receptors function in multiple ways to control vascular tone. Constriction of human pulmonary arteries induced by PGE_2_ is mediated by activation of EP3 receptor (Qian et al., 1994; Norel et al., 2004a), whereas EP1-receptor activation mediates constriction of human pulmonary veins (Walch et al., 2001). Indeed, EP1 antagonists enhance iloprost and PGE_2_-induced relaxation in human pulmonary veins and also underlie the contractions evoked by these two agents in the same tissue (Walch et al., 1999; Foudi et al., 2008). In large human pulmonary veins, relaxation to PGE_2_ is mediated by the EP4 receptor (Foudi et al., 2008), whereas the EP2 agonist ONO-AE1-259 produces relaxation at relatively high concentrations, although a nonselective effect on the EP4 receptor is not excluded (Foudi et al., 2008). It is possible that the different effects of PGE_2_ or EP-receptor agonists on pulmonary arteries as compared with veins are related either to differential expression of receptor subtypes or to differential coupling of receptors, such as the EP4 receptor (that can activate cAMP synthesis through G_s_ and also activate PI3K) (Hirata and Narumiya, 2012). On the other hand, IP-receptor agonists are well known to induce potent relaxation of pulmonary arteries (and veins) derived from human and rodent lungs (Haye-Legrand et al., 1987; Walch et al., 1999; Norel et al., 2004a,b; Benyahia et al., 2013, 2015). Apart from treprostinil, PGI_2_ and other stable analogs (iloprost and beraprost) appear to be weaker venous rather than arterial dilators (Benyahia et al., 2013), likely to reflect the differential expression of prostanoid receptors involved in regulating tone in the lung. It should be noted that the functional roles of EP-receptor subtypes on pulmonary vascular tone are not well studied in resistance vessels, particularly in relation to human lung microvessels.

Contraction and thickening of arterial vascular wall in lung are characteristics of PH. The urinary excretion of the stable metabolite of PGI_2_ (2,3-dinor-6-keto-PGF_1*α*_) is decreased in patients with PH compared with control patients (Christman et al., 1992). In parallel with this observation, the density of PGIS detected by immunohistochemistry was lower in the pulmonary arterial endothelium of patients with severe PH compared with controls (Tuder et al., 1999). Recently, diminution of PGIS density and 6-keto-PGF_1*α*_ levels in pulmonary artery, pulmonary artery smooth muscle cells, and distal lung tissue derived from patients in PH group-III have been described (Ozen et al., 2020a). Furthermore, PGIS polymorphisms appeared to protect against the development of PAH in families known to harbor mutations that are strongly linked to the disease, suggesting that PGIS might act as a modifier gene influencing the penetrance in hereditary PAH (Stearman et al., 2014). Very recently, three rare loss-of-function PGIS variants were found in patients with idiopathic PAH, providing evidence that PGIS might be a susceptibility gene for PAH, possibly by causing endothelial apoptosis (Wang et al., 2020). Interestingly, patients with variants of the gene coding for PGIS (*PTGIS*) were more sensitive to the vasodilatory effects of iloprost, although the nature of such potentiation remains unknown.

Ex vivo studies performed on preparations derived from lung or pulmonary arteries have consistently shown not only a reduction of PGI_2_ synthesis but also of the IP-receptor expression in both patients with PH and rats treated with monocrotaline and hypoxia that develop PH (Lai et al., 2008; Falcetti et al., 2010; Jiang et al., 2013; Li et al., 2018; Fan et al., 2019a; Clapp et al., 2020; Ozen et al., 2020a,b). The impairment of the PGI_2_ pathway in PH lungs underlies the rationale for why the administration of vasodilators, such as PGI_2_ or mimetics (IP agonists), are beneficial in the treatment of PH patients awaiting lung transplantation. This contrasts with EP4 and EP2 receptors, whose vascular expression was preserved or enhanced in human and experimental PAH, as confirmed by Western blot, real-time PCR, and immunohistochemical studies (Lai et al., 2008; Patel et al., 2018; Clapp et al., 2020). However, some of these IP agonists (epoprostenol, iloprost) have affinity for the EP1 receptor (Abramovitz et al., 2000; Whittle et al., 2012; Clapp and Gurung, 2015) with the potential of constriction of human pulmonary veins (Walch et al., 2001; Norel et al., 2004a). So far, there is no evidence for such a role of EP1 receptors in rodent pulmonary veins. Furthermore, some other IP agonists like treprostinil are also potent EP2 agonists (Whittle et al., 2012), which may combine with its potent activation of other prostanoid receptors (IP and DP1) to promote venodilation in pulmonary resistance vessels (Orie et al., 2013).

For these reasons, PGE_2_ and activation of the EP receptors are also of interest in PH. PGE_2_ concentrations in plasma were reduced in chronically hypoxic PH rats (Fan et al., 2019a), whereas in human pulmonary arteries exposed to hypoxia, increased levels of PGE_2_ were detected (Yang et al., 2002). Recently, it has been shown that EP2-receptor expression levels were increased in human pulmonary artery SMCs and in the lungs derived from PAH patients (Patel et al., 2018; Clapp et al., 2020). This may not be surprising, given that EP2-receptor expression can be enhanced in response to PDGF and transforming growth factor-*β* (TGF-*β*), which are key drivers of SMC proliferation in PAH (Clapp et al., 2020). Based upon the high relative abundance of EP2 over IP (84-fold) and the fact that treprostinil has a 10-fold greater affinity at the EP2 receptor compared with the IP receptor (Whittle et al., 2012), it can be predicted that treprostinil will be more than two orders of magnitude (>800-fold) more active at EP2 versus IP receptors in PAH cells. This might help explain why activation of the EP2 receptor becomes a more prominent mechanism than the IP receptor to drive inhibition of pulmonary SMC proliferation by treprostinil. It should be noted that EP2 receptors are even more heavily expressed in adventitial fibroblasts in PAH with little evidence of IP-receptor expression (Clapp et al., 2020). EP2 receptors are known to have a range of inhibitory effects on fibroblast function, inhibiting migration, proliferation, and the transition of fibroblasts to myofibroblasts. These findings offer a new therapeutic perspective of how vascular wall thickening and fibrosis could be targeted via a signaling pathway that is robustly expressed in PAH.

It is important to understand the role of contractile prostanoid receptors because these could limit the doses of PGI_2_ mimetics given therapeutically or potentially give rise to unwanted side effects. From studies conducted so far, it would appear that the EP3-receptor pathway is upregulated in PH, and this could occur for a number of reasons (Clapp et al., 2020). Increased expression of the EP3 receptor is found in human and mice pulmonary arteries after exposure to hypoxia by real-time PCR studies (Lu et al., 2015) in a monocrotaline model of PAH (Morrison et al., 2012) or in pulmonary arteries derived from PAH patients (Clapp et al., 2020). Furthermore, deletion of the EP3 receptor strongly inhibited the progression of PH induced by chronic hypoxia in rats. Similar results were obtained with the treatment of EP3-receptor antagonist, L-798106 (Lu et al., 2015). Likewise, increased sensitivity to the (EP3 > EP1) agonist sulprostone occurred in PAH arteries obtained from monocrotaline-treated rats, whereas beraprost (IP > EP3/TP agonist) caused contraction in distal human pulmonary arteries obtained from PAH patients with end-stage disease (Shen et al., 2019). Such studies suggest a gain of function of the EP3 receptor in PAH. This could be driven by EP3-TP–induced vasoconstrictive synergism, which has been described in numerous blood vessels, wherein priming with a TxA_2_ mimetic or *α*1 receptor agonist markedly increases both the potency and size of contraction to EP3 agonists (Benyahia et al., 2015; Clapp and Gurung, 2015). The consequence of enhanced EP3-receptor activation would be to lower cAMP levels via its coupling to G_i_, which would thus have the potential to counteract the effects of any IP-receptor agonist irrespective of whether they can directly activate these contractile receptors (Orie and Clapp, 2011). This may help to explain why vasorelaxation induced by PGI_2_ analogs is consistently enhanced over the entire concentration range when EP3 receptors are inhibited (Orie and Clapp, 2011; Morrison et al., 2012). Overall, these findings suggest that EP3-receptor antagonists could be a therapeutic target for PH. Thus enhanced EP3-receptor expression together with IP-receptor downregulation may curtail the action of prostacyclin in PAH patients with severe disease or limit their therapeutic efficacy (Clapp et al., 2020).

EP4-receptor expression was not modified in pulmonary arteries or lungs derived from chronically hypoxic PH rats or monocrotaline-treated rats and patients with PAH; this was demonstrated by real-time PCR, Western blot, and immunohistochemical studies (Lai et al., 2008; Li et al., 2018; Fan et al., 2019a). However, our recent study demonstrated that there is a decrease of EP4-receptor expression in bronchi derived from PH group-III patients by Western blot and real-time PCR techniques (Ozen et al., 2020b). Another recent study performed on hypoxia-induced PH rats revealed that the beneficial effect of beraprost (PGI_2_ analog) is mediated via the EP4 receptor–related pathway (Tian et al., 2019). In accordance with this finding, the EP4-receptor agonist, L-902,688, decreased PH right ventricular hypertrophy in hypoxic PH mice and monocrotaline-induced PH rats (Lai et al., 2018). In contrast, one study showed that during hypoxia, the vasoconstrictor effect of PGE_2_ is mediated through the activation of the EP4 receptor on the rat intrapulmonary artery (Yan et al., 2013).

Finally, PH as well as parturition, abortion, or gastrointestinal ulcers are the only domains in which EP and IP receptors are therapeutic targets in clinical practice. Better knowledge of the prostanoid receptors involved and the selectivity and the potency of the compounds used in these clinical conditions is therefore of utmost importance. Most of our knowledge about PH and the development of pharmacological/therapeutic strategies has focused on PH group-I (PAH). More studies in PH patients from other groups are necessary; the recently published work with human bronchi (Ozen et al., 2020b) suggests that inhaled PGI_2_ analogs may also have a promising therapeutic effect in PH group-III, which is one of the most common and lethal forms of PH. In PH group-III, PH is secondary to respiratory diseases, such as COPD, so in this case, inhaled PGI_2_ could have a dual effect by decreasing airway resistance, thus supplying more of this vasorelaxant drug and oxygen to pulmonary arteries.

## IV. Thrombosis

Platelets are involved in the development of atherothrombotic diseases, such as stroke and myocardial infarction, and, therefore, antiplatelet therapies are a mainstay in cardiovascular diseases (Kuriyama et al., 2010; Hubertus et al., 2014). In human and mouse platelets, activation of the AA pathway leads to the formation of various prostanoids (Mawhin et al., 2015). Although in this review we focused on the effects of PGE_2_ and PGI_2_ in thrombogenesis, it should be noted that other prostanoids, such as TxA_2_ and PGD_2_, are also involved in the regulation of thrombogenesis by inducing (TP receptor) and inhibiting (DP1 receptor) platelet aggregation, respectively (Armstrong, 1996; Song et al., 2012; Crescente et al., 2019).

### A. Role of Prostaglandin E_2_ on Platelet Function

Activated human platelets produce and release PGE_2_, although at 30-fold lower concentration than TxA_2_ (Petrucci et al., 2011). PGE_2_ does not activate platelet aggregation itself but has a concentration-dependent biphasic effect on the aggregation of human and mouse platelets (Philipose et al., 2010; Mawhin et al., 2015; Pasterk et al., 2015). These effects comprise potentiation of platelet aggregation at low concentration (nanomolar) and inhibition of platelet aggregation at higher concentration (micromolar) (Petrucci et al., 2011; Pasterk et al., 2015). It is known that PGE_2_ activates different membrane receptors on platelet named EP1, EP2, EP3, and EP4 (Petrucci et al., 2011; Pasterk et al., 2015). In mice, however, PGE_2_ at high concentration was also found to activate IP receptors (Fabre et al., 2001; Kuriyama et al., 2010).

#### 1. Expression of Prostaglandin E_2_ Receptors in Platelets

A large number of studies demonstrated that human and mouse platelets express EP2, EP3, and EP4 receptors (Paul et al., 1998; Kuriyama et al., 2010; Hubertus et al., 2014; Pasterk et al., 2015). On the other hand, although Petrucci et al. (2011) showed the presence of the EP1 receptor in human platelets, other studies were not able to detect EP1 receptor in human and mouse platelets (Ma et al., 2001; Hubertus et al., 2014). Supporting these latter studies, the EP1-receptor agonist, ONO-DI-004, and the EP1-receptor antagonist, ONO-8713, did not alter human platelet aggregation (Iyú et al., 2010).

#### 2. Prostaglandin E_2_ and Prostaglandin E_2_ Receptor 2

The action of PGE_2_ to inhibit platelet aggregation at high concentrations is mediated by two receptors, namely EP2 and EP4. Both receptors are coupled to the G_s_ protein and increase the intracellular cAMP concentration. Real-time PCR (RT-PCR) and Southern blot analysis demonstrated that there is relatively low expression of EP2 receptor in human and mice platelets (Paul et al., 1998; Ma et al., 2001). Despite the low levels of EP2 receptors, the EP2-receptor agonists butaprost and ONO-AE1-259 inhibited platelet aggregation induced by the TP-receptor agonist U-46619 in human and mouse platelets to a similar extent (Kuriyama et al., 2010; Smith et al., 2010). This effect of the EP2-receptor agonist was absent in platelets of EP2-receptor knockout mice (Kuriyama et al., 2010).

#### 3. Prostaglandin E_2_ and Prostaglandin E_2_ Receptor 3

Multiple isoforms of the EP3 receptor are present in human platelets, whose structures differ only by their carboxy-terminal tails responsible for the specificity for G-proteins (Kotani et al., 1995; Paul et al., 1998). EP3 has the potential to couple to G_s_, G_i_, or G_q_ proteins. In human platelets, four isoforms of the EP3 receptor (termed EP3-1b, EP3-II, EP3-III, and EP3-IV) were detected (Paul et al., 1998). EP3 splice variant distribution and function remain to be determined, but the overall effect of the EP3 receptor in human and mouse platelets is inhibition of cAMP production via G_i_ protein (Gray and Heptinstall, 1991; Ma et al., 2001). The activation of EP3 receptor potentiated platelet aggregation induced by different agents in both humans and mice (Philipose et al., 2010; Petrucci et al., 2011; Hubertus et al., 2014). These in vitro results were also confirmed in vivo using EP3 knockout mice, in which thrombotic responses to AA were decreased (Ma et al., 2001; Gross et al., 2007). Similar results were obtained in ferric chloride–induced thrombosis in mice (Gross et al., 2007).

To investigate the role of EP3 in thrombosis, EP3-receptor antagonist (DG-041) and agonists [sulprostone (EP3 > EP1 agonist), 17-phenyl trinor PGE_2_ (EP1 agonist)] were used. EP3-receptor agonists increased platelet aggregation in humans and mice induced by different platelet agonists (Heptinstall et al., 2008; Pasterk et al., 2015; Theiler et al., 2016). Moreover, the (EP3 > EP1) receptor agonist, sulprostone, augmented the adhesion of human platelets to fibrinogen and collagen under low shear stress. This effect was prevented by the EP3-receptor antagonist L-798106 (Pasterk et al., 2015). Potentiation of PGE_2_-induced platelet aggregation was inhibited by DG-041 in human, rat, and mouse platelets (Heptinstall et al., 2008; Singh et al., 2009; Smith et al., 2010; Tilly et al., 2014). In vivo studies performed on mice also demonstrated that DG-041 reduced thrombosis but had no effect on bleeding time (Tilly et al., 2014). In line with these findings, one clinical study performed on healthy volunteers demonstrated that DG-041 inhibited platelet function without increasing bleeding time (Fox et al., 2013). EP3-receptor antagonists could therefore be a novel therapeutic approach for the treatment of atherothrombosis without increasing the risk of hemorrhage, such as stroke or GI bleeding.

#### 4. Prostaglandin E_2_ and Prostaglandin E_2_ Receptor 4

The inhibitory effect of PGE_2_ on human platelet aggregation was abolished in the presence of MF-191, an EP4-receptor antagonist. Moreover, EP4-receptor agonist, ONO-AE1-329, inhibited platelet aggregation induced by the TP-receptor agonists U-46619, adenosin diphosphate, or collagen in human and mouse platelets. These data suggested that the inhibition of platelet aggregation by PGE_2_ is mediated by EP4 receptor in humans (Philipose et al., 2010; Smith et al., 2010) and mice (Kuriyama et al., 2010). However, there is a greater inhibitory potency of ONO-AE1–329 in human platelets than in mouse platelets (Kuriyama et al., 2010; Mawhin et al., 2015). The PGE_2_-induced inhibition of platelet aggregation was dramatically increased in EP3 and IP double-knockout mice, suggesting that EP2- and EP4-mediated inhibitory effect is augmented when the EP3 receptor is absent (Kuriyama et al., 2010). In both human and mouse platelets, the potentiating effect of PGE_2_ on platelet aggregation via EP3 receptor is predominant over any inhibitory effects of EP2 and EP4 receptors (Gross et al., 2007; Iyú et al., 2010; Kuriyama et al., 2010; Hubertus et al., 2014). The inhibitory role of the EP4 receptor had probably been concealed for some time by the fact that it is the IP receptor rather than the EP2 or EP4 receptors that mediate the inhibitory effect of PGE_2_ on mouse platelets, as revealed by studies using IP knockout mice (Fabre et al., 2001; Kuriyama et al., 2010).

### B. Role of Prostacyclin on Platelet Function

Among prostanoids, PGI_2_ is the most potent inhibitor of human and rat platelet aggregation by binding to its cognate IP receptor (Jones et al., 2006; Smith et al., 2010; Crescente et al., 2019). IP-receptor expression was demonstrated in both human and mouse platelets (Kuriyama et al., 2010; Tourdot et al., 2017). IP receptor activates AC through G_s_ protein and increases the production of cAMP. High levels of cAMP activate PKA, which suppresses various signaling pathways involved in platelet function. For example, activation of PKA decreases the release of Ca^2+^, thereby reducing the activation of cytosolic phospholipase A_2_ and release of AA from the phospholipid membrane, which in turn decreases the production of prostanoids (i.e., TxA_2_ by platelets) (Crescente et al., 2019). The in vivo role of PGI_2_ in platelet aggregation was also examined in IP-receptor knockout mice, which showed pronounced susceptibility to thrombosis in response to ferric chloride associated with elevated thromboxane levels (Murata et al., 1997).

The role of PGI_2_ in atherothrombosis in humans was highlighted by the cardiovascular side effects observed with COX-2 inhibitors, such as rofecoxib (VioxxTM) and celecoxib (CelebrexTM). FitzGerald and colleagues demonstrated that inhibition of PGI_2_ biosynthesis by these drugs could lead to hazardous cardiovascular events, including myocardial infarctions and thrombotic stroke (Catella-Lawson et al., 1999; McAdam et al., 1999). Furthermore, polymorphisms in PGIS gene in humans were found to be associated with myocardial infarction (Nakayama et al., 2002b) or enhanced platelet activation in patient with deep venous thrombosis or stroke (Patrignani et al., 2008; Shimizu et al., 2013). The role of platelets as a disease-modifying process in PAH is not well understood but is of particular interest because platelets produce and release vasoactive substances, such as TxA_2_, 5-hydroxytryptamine, and platelet-derived growth factor, which may cause harmful vasoconstriction and contribute to vascular remodeling in PAH. With the exception of selexipag, which is a highly selective, nonprostanoid IP-receptor agonist devoid of activity at other prostanoid receptors (Bruderer et al., 2014), all other IP agonists used clinically or being developed (e.g., ralinepag) will inhibit platelet function in vivo (Clapp et al., 2020). The reason for this differential effect on platelet function may be explained in part by selexipag acting as a partial agonist in cAMP assays (Gatfield et al., 2017). As already discussed, platelets have an active EP3 and TP-receptor system, which will negatively regulate basal cAMP levels, thereby likely preventing selexipag from generating enough cAMP to oppose platelet aggregation by endogenous circulating TxA_2_ and PGE_2_. Interestingly, the novel PGI_2_ mimetic ONO-1301 does not undergo receptor desensitization in platelets because of its inhibitory action on TxA_2_ synthesis, suggesting that the TP-receptor activation is strongly linked to IP-receptor desensitization (Kashiwagi et al., 2015), which is particularly relevant when TxA_2_ levels rise as they do in PAH and in other cardiovascular diseases.

## V. Central and Peripheral Nervous System

### A. Central Nervous System

Many studies in humans and in mouse models showed important roles of PGE_2_ and PGI_2_ in the development and/or progression of central nervous system disorders (Bazan et al., 2002; Yagami et al., 2016).

#### 1. Alzheimer Disease

Alzheimer disease (AD) is an age-related dementia that is not only characterized by *β* amyloid (A*β*) protein aggregation and accumulation but also by *τ* protein hyperphosphorylation in neurons (Guan and Wang, 2019). Neuroinflammation seems to play a critical role in the physiopathology of AD (Akiyama et al., 2000). Indeed, PGE_2_ levels in CSF were increased in AD patients with mild memory impairment, whereas PGE_2_ levels were decreased in end-stage AD patients (Combrinck et al., 2006). Interestingly, neuronal COX-2 expression was also reported to be downregulated in end-stage AD patients (Yermakova and O’Banion, 2001). mPGES-1 enzyme was present in neurons, microglia, and endothelium in human healthy brains, but its expression levels were upregulated in both mRNA and protein levels in AD patients (Chaudhry et al., 2008). The mPGES-1 gene disruption protected neurons from cytotoxic effect of A*β* 31–35 fragment in mice (Kuroki et al., 2012), indicating that mPGES-1-PGE_2_ axis takes part in A*β*-eliciting harmful effects on neurons.

The role of EP1 receptor is still not clear in AD initiation and progression in humans. The genetic deletion of EP1 receptor decreased basal levels of A*β* in Swedish amyloid precursor protein (APPS)/presenilin-1 (PS1) mice model (APPS- and PS1-mutated AD model), and therefore, neurons in the absence of EP1 receptor are presumably more resistant to A*β*-induced toxicity (Zhen et al., 2012). The exacerbating role of EP1-receptor signaling was also suggested by an in vitro study: the blockade of EP1-receptor signaling with its antagonist (SC51089) in human neuroblastoma cell line (MC65) resulted in approximately 50% of reduction of A*β*-induced neurotoxicity (Li et al., 2013).

The genetic deletion of EP2 receptor in APPS mice decreased the protein levels of A*β*40 and A*β*42 as well as oxidative stress. Based on this study, an EP2-receptor signaling is considered to elicit proinflammatory and proamyloidogenic actions, at least in this AD model (Liang et al., 2005).

Another likely possibility is that EP2-receptor pathway may potentiate phagocytosis of A*β*42 by microglia in mouse AD model. Indeed, EP2 receptor has been shown to activate microglial function in other neurologic disorders, such as Parkinson Disease (PD) (see next paragraph). It was also reported in mouse primary neurons that pharmacological activation of EP2 or EP4 receptor rescued A*β*42-induced cell death in a cAMP-dependent manner (Echeverria et al., 2005). However, there is no strong evidence showing a protective role of EP2 pathway against AD in humans, and to date there is no ongoing clinical trial by targeting EP2 receptor for the treatments of AD patients (Cudaback et al., 2014).

Quantitative Western blot and immunohistochemistry analysis with human temporal cortex (postmortem tissues) demonstrated increased expression of EP3 receptor in mild cognitive impairment and a further increase in AD patients (Shi et al., 2012). These results were in accordance with the elevated expression of the EP3-receptor mRNA in hippocampus of mild-age APPS mice (AD model). The deletion of the EP3 receptor in these APPS mice decreased both A*β*40 and A*β*42 protein levels, with an attenuation of neuroinflammation and the reduction of proinflammatory gene expression, cytokine production, and oxidative stress (Shi et al., 2012). A recent study described a particular aspect of the EP3 pathway in APPS/PS1 mice; the treatment with EP3 > EP1 agonist, sulprostone, impaired synaptic plasticity of specific neurons in the hippocampus. Such a harmful effect of sulprostone was reversed by ONO-AE3-240, an EP3-receptor antagonist (Maingret et al., 2017). The studies on AD patients still remain to be evaluated for the use of EP3-receptor antagonists for clinical purposes (Cudaback et al., 2014).

EP4-receptor pathway involves the regulation of immune and proinflammatory responses in mouse AD models (Woodling and Andreasson, 2016). Indeed, the pharmacological suppression of EP4 receptor by using ONO-AE3-208 and also the genetic ablation of EP4 signaling lead to an improvement of cognitive function in mouse AD model (Hoshino et al., 2012). Nevertheless, EP4-targeted strategies have not been carried out clinically (Cudaback et al., 2014).

On the other hand, it was reported that PGI_2_ counteracts PGE_2_-induced IFN-*γ* production levels in mouse brain in an A*β*-dependent mechanism (Wang et al., 2016b). Interestingly, another group also demonstrated that PGI_2_ ameliorated the exacerbating effect of PGE_2_ on cognitive disorder in APPS/PS1 mice (Zheng et al., 2017). Overall, the studies investigating the role of IP and EP receptors in AD are mostly restricted to animal models, thus further studies are warranted to examine whether and how PGI_2_ and PGE_2_ are involved in the development and/or treatment of AD in humans.

#### 2. Parkinson Disease

PD is characterized by a progressive loss of dopaminergic neurons in the substantia nigra. This complex and multifactorial disease could be explained by several molecular and cellular mechanisms, including neuroinflammation and microglial dysfunctions. The levels of PGE_2_ were reported to be significantly elevated in the substantia nigra and other areas of PD patients’ brains (Mattammal et al., 1995).

EP1 receptor has been suggested to mediate PGE_2_-induced neurotoxicity in rat dopaminergic neurons isolated from substantia nigra: EP1-receptor agonist 17-phenyl trinor PGE_2_ induced toxic effects on dopaminergic neurons along with PGE_2_, and 16-phenyl tetranor PGE_2_ (stable analog of PGE_2_) induced toxic effects on dopaminergic neurons, whereas EP2-receptor agonist butaprost and the (EP3 > EP1) receptor agonist sulprostone failed to exert any toxic actions (Carrasco et al., 2007). In rat microglia, an EP2-receptor signaling was reported to activate microglial functions in a cAMP-dependent manner, and thus, EP2 pathway appears to aggravate neuroinflammation. Indeed, EP2-receptor agonist, butaprost, as well as PGE_2_ stimulated cAMP production in microglial cells, whereas EP1-receptor agonist, 17-phenyl trinor PGE_2_, and EP4-receptor agonist, CAY10598, failed to elicit such actions. It is interesting that EP2-receptor activation exacerbated the rapid upregulation of mRNAs of proinflammatory genes, such as COX-2, IL-6, and iNOS (Quan et al., 2013). Kang et al. (2017) also found that COX-2–PGE_2_-EP2-cAMP axis is involved in oxidopamine-induced neurotoxic effects in Neuro-2a (mouse neuroblastoma) and SH-SY5Y (originated from human bone marrow of a 4-year-old patient with neuroblastoma) cell lines, both of which mainly consist of dopaminergic neurons. Moreover, Johansson et al. (2013) demonstrated in an 1-methyl-4-phenyl-1,2,3,6-tetrahydropyridine–induced PD model that microglia-specific EP2 deficiency attenuates disease-induced microglial activation and prevents a loss in dopaminergic neurons in substantia nigra. In contrast to such an in vivo study, in rat primary in vitro midbrain cultured cells, an EP2-receptor pathway appears to exhibit a neuroprotective effect on dopaminergic neurons (Carrasco et al., 2008).

Although EP4 receptor is coupled to cAMP stimulation like EP2, the EP4-receptor pathway substantially works in a neuroprotective direction: in mouse in vivo model of PD, microglia-specific EP4 deficiency exacerbated microglial activation and T-cell infiltration in substantia nigra, and systemic administration of an EP4 agonist prevented a loss of dopaminergic neurons (Pradhan et al., 2017).

The exact role of PGI_2_ in PD remains to be determined. However, it was reported that enforced PGI_2_ synthesis by adenoviral gene transfer into substantia nigra prevents loss of dopaminergic neurons in oxidopamine-induced PD model. Based on this report, PGI_2_ has a potential to exert a neuroprotective action in PD (Tsai et al., 2013). Further studies on the role of EP and IP receptor in patients with PD are necessary.

#### 3. Huntington Disease

Huntington Disease (HD) is a neuropathology in which a genetic mutation can cause a large spectrum of symptoms, such as chorea and other motor disorders, but also cognitive disorders caused by an atrophy in basal ganglia (Anglada-Huguet et al., 2014).

In addition to the genetic etiology, the role of inflammation has been also described in the progression of HD. In fact, EP1-receptor antagonist, SC51089, has been shown to slow down the motor disorders and to ameliorate long-term memory decline in a mouse model of HD. Thus, the antagonism of EP1 receptor appears to improve many disorders in HD (Anglada-Huguet et al., 2014).

Another study from the same group demonstrated that an EP2/EP3/EP4 agonist, misoprostol, can also reduce memory decline in mouse model of HD. Authors concluded that among EP receptors, EP2 receptor promotes synaptic plasticity and delays neurodegeneration by stimulating the brain-derived neurotrophic factor expression (Anglada-Huguet et al., 2016). Despite these recent works, the impact and roles of IP/EP receptors in the pathophysiology of HD remain to be elucidated in humans also.

#### 4. Multiple Sclerosis in Central Nervous System

MS is a chronic demyelinating disease of the CNS that leads to permanent cognitive and motor disabilities. MS is characterized by inflammation, oligodendrocyte loss, and axonal pathology. It was reported that both PGE_2_ levels in CSF and COX-2 expression levels in demyelinating plaques are increased in MS patients, suggesting that COX-2–PGE_2_ axis is involved in neuroinflammation (Mattsson et al., 2009; Palumbo et al., 2012). In addition, Kihara et al. (2009) demonstrated by using lipidomics that the main products of eicosanoid synthesis pathway in spinal cord are shifted from PGD_2_ into PGE_2_ in association with the onset of mouse MS model. In addition, they found that mPGES-1 deficiency can attenuate the symptoms of the disease, and there is a positive correlation between mPGES-1 immunoreactivity in microglia/macrophages and the severity of MS disease, indicating that mPGES-1-PGE_2_ pathway plays a role in the progression of MS (Kihara et al., 2009). Kihara et al. (2009) proposed that at least EP1/EP2/EP4 receptors may participate in the PGE_2_-elicited MS pathogenesis in various cell types, and the suppression of mPGES-1 activity by specific inhibitor may be a potential treatment against MS in humans.

In another mouse model of MS (induced by cuprizone), COX-2–PGE_2_-EP2 axis has been proposed to play an important role by aggravating oligodendrocyte apoptosis during the onset of MS: AH6809 (EP1/2 and DP1 antagonist), reduced cuprizone-induced oligodendrocyte apoptosis, demyelination, neuroinflammation, and motor deficits. Indeed, the gene expression levels of EP receptors, including EP2, in brain were upregulated in the progressive stage of MS (after 5 weeks of cuprizone treatment) (Palumbo et al., 2012). In this MS model, PGI_2_ level in the CSF is likely to be unchanged upon cuprizone administration (Palumbo et al., 2012). Interestingly, it was reported in mouse model of MS that a stable IP agonist, iloprost, suppresses demyelination and motor dysfunction, indicating that an IP-receptor agonist has a potential to prevent (or alleviate) symptoms of MS (Muramatsu et al., 2015). However, there are no data available on the role of IP receptor in humans with MS.

#### 5. Amyotrophic Lateral Sclerosis

Amyotrophic lateral sclerosis (ALS) is a progressive neurodegenerative disease primarily involving motor neurons and characterized by several molecular and cellular dysfunctions, including oxidative stress, apoptosis, neuroinflammation, and glutamate toxicity. This disease has been shown to be closely associated with upregulation of proinflammatory pathway; PGE_2_ levels in CSF, brain, and plasma were all increased in ALS patients (Almer et al., 2002; Iłzecka, 2003).

EP2 receptor has been shown to participate in neuroinflammation and progression of ALS in mouse model of familial ALS containing G93A-mutation in superoxide dismutase gene. The EP2-receptor deficiency improves motor strength and extends survival in association with systemic reductions in the levels of proinflammatory effectors, such as iNOS and NADPH oxidase, suggesting that suppression of EP2-receptor signaling may be a novel strategy for the treatment of ALS (Liang et al., 2008). It should be noted that the EP2-receptor signaling can induce the motor-neuron–like cell death in an in vitro system (Miyagishi et al., 2013).

The mRNA expression levels of alternatively spliced isoforms of EP3 receptor were characterized during the pathogenesis of ALS; EP3*α* and EP3*γ* mRNAs were detected in the WT lumbar spinal cord, but EP3*β* mRNA was undetectable. When the authors analyzed motor neurons dissected out of spinal cord, EP3*γ* mRNA was predominantly detected in motor neurons, whereas EP3*α* and EP3*β* mRNAs were undetectable. Such an EP3*γ*-biased expression in motor neurons was unchanged in ALS-model (G93A-mutated in superoxide dismutase gene) mice (Kosuge et al., 2015).

Bilak et al. (2004) reported that the EP2-receptor agonist butaprost as well as the EP3 > EP1 agonist sulprostone exerted a neuroprotective effect on motor neurons in slice culture model of ALS. It remains to be elucidated why G_s_-coupled EP2 and G_i_-coupled EP3 pathways share similar protective actions in chronic glutamate–induced ALS model.

Interestingly, it was reported that administration of the IP-receptor agonist, ONO-1301-MS, to ALS-model mice attenuates the expression of hypoxia-inducible factor 1*α*, which is a hypoxia marker known to be elevated in the spinal cord of mouse model of ALS and ALS patients (Tada et al., 2019). There has been no study yet on the role of PGI_2_ in humans with ALS. Moreover, although PGE_2_ levels have been shown to increase in patients with ALS, further studies are necessary to confirm the contribution of each EP-receptor subtype in these patients.

### B. Peripheral Nervous System

#### 1. Prostaglandin E_2_ and Prostacyclin Receptors in Peripheral Nervous System/Dorsal Root Ganglion Neurons

Aspirin and other NSAIDs are used for several pharmaceutical actions, especially as analgesics. Inflammation, caused by injury or other reasons, could be then perceived by the brain and all other systems as a signal of danger and/or attention through pain. PGE_2_, through its EP1**–**4 receptors, and PGI_2_, through its IP receptor, are considered to be pain mediators.

Dorsal root ganglion (DRG) neurons play an important role in the pain perception (i.e., nociception) and pain transmission. IP, EP1, EP3, and EP4 receptors were detected in DRG neurons of mice with in situ hybridization studies (Sugimoto et al., 1994; Oida et al., 1995). Another study also demonstrated that EP1, EP2, and EP4 receptors and only EP3*γ* isoform (but not EP3*α* or EP3*β*) were expressed in rat dissociated sensory DRG neurons with PCR detection (Southall and Vasko, 2001).

Several pharmacological studies focused on the roles of EP and IP receptors in DRG neurons, indicating that only IP and EP4 receptors are involved in cAMP production. An IP-receptor agonist, cicaprost, and EP4-receptor agonist, ONO-AE1-329, increased cAMP levels in rat DRG neurons in a concentration-dependent manner. On the other hand, ONO-DI-004 (EP1-receptor agonist), ONO-AE1-259 (EP2 agonist), sulprostone (EP3 > EP1 agonist), and ONO-AE-248 (EP3 agonist) were unable to alter cAMP levels, suggesting that PGE_2_-induced cAMP production appears to be mediated by EP4 receptor without being affected by EP3 receptors (Wise, 2006).

#### 2. Roles of Prostaglandin E_2_ Receptor 3 in Pain Perception

Several studies have been performed to clarify the role of EP3 receptor in pain perception. Pharmacological stimulation with the EP3-receptor agonist, ONO-AE-248, resulted in antinociceptive effect in rat models of joint inflammation (Natura et al., 2013). Another study demonstrated in a mechanical nerve ligation-induced neuropathic pain model that among the four EP receptors, only EP3 deficiency attenuated nociceptive behavior and mechanical allodynia. Recently, EP3-induced CCL2 chemokine release has been proposed as a possible mechanism underlying EP3-induced neuropathic pain (Treutlein et al., 2018). Thus, EP3-receptor antagonist could be a therapeutic target to reduce chronic neuropathic pain, and studies performed in humans with neuropathic pain are necessary.

#### 3. Roles of Prostaglandin E_2_ Receptor 1, Prostaglandin E_2_ Receptor 2, Prostaglandin E_2_ Receptor 4, and Prostacyclin Receptor in Pain Perception

Apparently, EP1 receptor is involved in pain perception through its major role in peripheral nervous system (PNS) but not in CNS. The studies using EP1 knockout mice revealed that EP1 receptor is implicated in the perception of inflammation induced-heat/pain sensitization in PNS (Johansson et al., 2011). Moriyama et al. (2005) demonstrated that EP1 deficiency attenuates the activation efficiency of TRPV1, a nonselective cation channel, which is expressed in sensory neurons and activated by various noxious stimuli, such as heat, proton, and pepper constituent. Moreover, EP1-receptor antagonist, ONO-8713, mimicked the effect of EP1 deficiency, whereas an EP1-receptor agonist, ONO-DI-004, exacerbated capsaicin-induced TRPV1 activation in mice DRG neurons. The mechanisms underlying PGE_2_-induced TRPV1 potentiation are likely different between human and mouse DRG neurons. PGE_2_-induced TRPV1 potentiation was suppressed by the activation of metabotropic glutamate receptors 2 and 3 in mouse but not human DRG neurons (Sheahan et al., 2018). The inhibition of pain perception by selective EP1 antagonist remains to be confirmed in human models.

Inflammatory pain occurs in endometriosis. In a preclinical mouse model of endometriosis, EP2 receptor has been shown to mediate peripheral and central hyperalgesia. Real-time PCR studies demonstrated that the expression level of EP2/EP4 receptors and also COX-2 were significantly increased in endometriosis lesions compared with controls. An EP2-receptor antagonist, PF-04418948, was the most efficiently analgesic rather than EP4- or TRPV1- antagonist, since this drug showed suppressive effects on both peripheral and central hyperalgesia (Greaves et al., 2017). Using an chimeric endometriosis model in which human endometriotic cells were xenografted into nude mice, Arosh et al. (2015) demonstrated that selective inhibition of both EP2 and EP4 receptors suppresses proinflammatory state of DRG neurons and attenuates pelvic pain in endometriosis. Lin et al. (2006) reported that EP4 receptor plays pivotal roles in nociception and promotes inflammatory pain hypersensitivity, at least in mouse model. Indeed, they found that EP4-receptor expression is increased both in mRNA and protein levels in DRG neurons upon peripheral inflammation (and EP1-3 expression levels are unchanged) (Lin et al., 2006). The use of EP4-receptor antagonists, such as MF498 or AH23848, was associated with pain reduction, and hence, these drugs had analgesic effects in murine inflammation models (Lin et al., 2006; Clark et al., 2008). These analgesic actions of EP4-receptor antagonists have already been verified in humans (Jin et al., 2018), and these drugs may serve for the treatment of pain-associated inflammatory diseases, such as rheumatic disease or osteoarthritis. Indeed, Grapiprant, an EP4 antagonist, is already approved for the treatment of pain and inflammation in osteoarthritis in dogs (Rausch-Derra et al., 2016).

The IP receptor is highly involved in nociception, hyperalgesia, and inflammation. The effect of IP-receptor activation on the sensitization of rat sensory neurons is mediated by stimulation of adenylyl cyclase and phospholipase C in sensory neurons (Smith et al., 1998). Furthermore, the involvement of IP receptors in inflammatory pain was addressed by using IP-receptor knockout mice and acetic acid–induced writhing test (Murata et al., 1997; Bley et al., 1998). The observed effect was also confirmed by using two selective IP antagonists, RO1138452 (CAY10441) and RO3244794 (Bley et al., 2006). These antagonists were tested in a rat model of nociception; both antagonists inhibited carrageenan-induced mechanical hyperalgesia and edema formation (Bley et al., 2006). Furthermore, in a rat model of neuropathic pain, a stable PGI_2_ analog (carbaprostacyclin) increased the neuronal activities in DRG and dorsal horn in a dose-dependent manner (Omana-Zapata and Bley, 2001).

The inhibition of PGI_2_-IP pathway in rodent models of hyperalgesia and chronic arthritis showed a significant reduction of pain and associated inflammation. In this study, the injection of a prostacyclin analog, beraprost (IP > EP3/TP agonist), dose-dependently induced hyperalgesia, and such an effect was abolished by the simultaneous administration of IP-receptor antagonist (*N*-[4-(imidazo- lidin-2-ylideneamino)-benzyl]-4-methoxy-benzamide) (Pulichino et al., 2006). In contrast, a 7-day clinical use of another prostacyclin analog, iloprost (IP = EP1 > EP3 agonist), in rheumatoid arthritis patients demonstrated that iloprost has anti-inflammatory and analgesic properties (Gao et al., 2002). Another study reported on the analgesic effect of iloprost being similar to the use of tramadol, a powerful analgesic, in a clinical trial for patients with arthritis (Mayerhoefer et al., 2007). Such different efficacies of beraprost and iloprost could be explained by species difference and/or the different specificity profiles between iloprost (IP = EP1 > EP3) and beraprost (IP > EP3/TP) (Whittle et al., 2012; Alexander et al., 2019).

In patients with pulmonary hypertension, so far all IP-receptor agonists produce adverse events related to pain, including site pain, jaw pain, flushing, headache, and extremity pain, suggesting a key role of the IP receptor in mediating pain (Picken et al., 2019). Given that the highest odds ratio for jaw pain from meta-analysis was seen with beraprost (Picken et al., 2019), this might indicate involvement of EP3 in the joint pain. Transitioning from epoprostenol (synthetic PGI_2_) to subcutaneous treprostinil increases site pain (Rubenfire et al., 2007), again suggesting additional receptors contributing—probably EP2 and DP1 because they are both expressed in the skin, and EP2 is expressed in the dorsal horn—which receive sensory information from primary afferents. One of the mechanisms underlying PGI_2_-elicited pain modulation is explained by the potentiation of the TRPV1 receptor in mouse DRG neurons (Moriyama et al., 2005). Alternatively, as suggested for the role of PGE_2_, PGI_2_-IP signaling is also likely to sensitize glutamate pathway by inducing phosphorylation and translocation of GluR1 receptor in mouse DRG neurons in zymosan-induced mechanical hyperalgesia model (Schuh et al., 2014).

## VI. Respiratory System

Endogenous PGE_2_, secreted by epithelial cells, endothelial cells, SMCs, macrophages, and fibroblasts, exerts complex effects on resident and infiltrating lung cell types. The predominant effects of PGE_2_ on the lung, as opposed to many other tissues/organs, are considered to be anti-inflammatory and protective (Vancheri et al., 2004; Safholm et al., 2015). Nevertheless, the use of exogenous PGE_2_ as a pharmacological/therapeutic tool in patients with lung diseases is limited because of the multiplicity of EP receptors types with receptor-specific effects that can be both beneficial or detrimental in patients. For instance, in asthma, the effects of PGE_2_ or different EP receptors agonists can have beneficial effects on bronchial SMC, such as inhibition of proliferation through EP2 and EP4 receptors (Zaslona and Peters-Golden, 2015) and relaxation via EP2 in mice and EP4 in humans (Buckley et al., 2011; Benyahia et al., 2012). PGE_2_ could also have a detrimental effect by promoting cough through the EP3 receptor (Maher et al., 2009).

Because clinical pharmacology/therapeutic interventions require both a reductionist and an integrated approach, we present the effects of PGE_2_ and the involvement of different EP-receptor types in the lung at three levels of integration: 1) cell types/tissue; 2) pathophysiologic processes that are common to many lung diseases [e.g., contraction or proliferation of SMC; pulmonary vascular remodeling (Lundequist et al., 2010)]; and 3) specific diseases (e.g., asthma, COPD, pulmonary fibrosis, etc.).

### A. Bronchial Smooth Muscle Cells

The effects of PGE_2_ on human bronchial SMC are well documented and globally considered as being “bronchoprotective.” Bronchoprotection is explained by several direct and indirect mechanisms. Low (<1 µM) concentrations of PGE_2_ attenuated histamine- or anti-IgE–induced contraction in small (<1 mm in diameter) and larger human bronchi ex vivo through an EP4 receptor–dependent effect (Buckley et al., 2011; Benyahia et al., 2012; Safholm et al., 2015; Ozen et al., 2020b). This EP4 receptor–mediated relaxation was related to a direct effect on the human bronchial SMC. Interestingly, in mice, relaxation of bronchial SMC was mediated by activation of EP2 receptors (Sheller et al., 2000), whereas in humans, it was mediated via the EP4 receptor (Benyahia et al., 2012), although EP2 receptors were reported to inhibit mast cell–induced bronchoconstriction (Safholm et al., 2015). Cooperativity between EP2 and EP4 receptors on human bronchial SMC growth inhibition has been demonstrated (Michael et al., 2019). Higher concentrations of PGE_2_ (10–100 µM) contracted human bronchi preparations by activating TP receptors (Safholm et al., 2015). On the other hand, PGI_2_ and its analogs (iloprost, treprostinil) induced potent bronchodilation ex vivo in bronchial preparations derived from either pathologic (PH, COPD, lung fibrosis, emphysema) or nonpathologic (Haye-Legrand et al., 1987; Norel et al., 1999; Ozen et al., 2020b) human lung specimens. In a similar way, in a rat model of PH, iloprost treatment was effective at reducing bronchial hyper-reactivity induced by methacholine (Habre et al., 2011). Although the majority of effects of PGI_2_ analogs on bronchial muscle tone have been attributed to the IP receptor, EP4 and DP1 receptors may also contribute to the actions of treprostinil, particularly at higher analog concentrations (Ozen et al., 2020b).

Indirect effects that explain PGE_2_-related “bronchoprotection” are mediated by: 1) activation of the EP2 receptors on mastocytes, which inhibits the IgE-triggered mediators release from mastocytes (Safholm et al., 2015); 2) inhibition of allergen-stimulated PGD_2_ release (Hartert et al., 2000); and 3) inhibition of eosinophil responses (e.g., chemotaxis and degranulation), effects that are mediated by both EP2 and EP4 receptors (Peinhaupt et al., 2017). In addition, the PGE_2_-mediated activation of the EP4 receptors inhibited the interaction (adhesion and transmigration) of eosinophils with endothelial cells (Peinhaupt et al., 2017).

Globally, in asthma, the prevention of the early airway response to allergen depends on bronchodilation and inhibition of the release of mast-cell mediators, such as histamine, leukotrienes, and PGD_2_ (Vancheri et al., 2004); protection against allergen-induced airway hyper-responsiveness and late asthmatic reaction (>24 hours) is secondary to the reduced recruitment of inflammatory cells (Vancheri et al., 2004). Other delayed, potentially protective effects of PGE_2_ on bronchial SMC are related to inhibition of migration mediated through both EP2 and EP4 receptors (Lebender et al., 2018). Another interesting protective effect of PGE_2_ concerns aspirin-induced asthma characterized by decreased PGE_2_ secretion by peripheral blood cells and lung epithelial cells with resulting increased synthesis of cysteinyl-leukotrienes and bronchoconstriction (Vancheri et al., 2004). Inhaled PGE_2_ can decrease the release of cysteinyl-leukotrienes from blood leukocytes occurring after aspirin challenge in patients with aspirin-induced asthma (Vancheri et al., 2004).

### B. Effects of Prostaglandin E_2_ and Prostacyclin on Lung Fibroblasts

In human or rodent lungs, the increased production of cAMP after EP2-, EP4-, or IP-receptor activation are known to induce antifibrotic signaling by decreasing fibroblast proliferation, motility, and extracellular matrix synthesis (Insel et al., 2012).

The IP receptor is expressed in primary human lung fibroblasts from patients with and without idiopathic pulmonary fibrosis. In a recent publication, it was shown that ACT-333679 (MRE-269, a selective IP agonist) exerts potent antifibrotic effects on primary human lung fibroblasts by reducing Yes-associated protein/transcriptional coactivator with PDZ-binding motif–dependent profibrotic gene transcription (Zmajkovicova et al., 2019). A similar protective effect was found in rats submitted to inhalation of nanoparticle INS1009 containing treprostinil (IP agonist) prodrug (C16TR), which inhibited bleomycin-induced pulmonary fibrosis (Corboz et al., 2018). In other studies, iloprost and treprostinil protected against bleomycin-induced pulmonary fibrosis (Zhu et al., 2011; Nikitopoulou et al., 2019). In both studies, mice treated with bleomycin+iloprost showed a normal alveolar structure and reduced lung inflammation compared with those treated with bleomycin alone, with lower proinflammatory cytokine (TNF-*α*, IL-6, TGF-*β*1) concentrations in broncho-alveolar lavage reported in the former study and reduced inflammatory cell infiltration in the latter.

PGE_2_ has a range of inhibitory effects on human fibroblast function, including inhibition of chemotaxis, TGF-*β–*induced transition of fibroblasts into myofibroblasts, collagen synthesis, and cell proliferation (Li et al., 2011). In terms of the role of prostanoid receptors involved, lung fibroblasts treated with EP1- and EP3-receptor agonists showed enhanced chemotaxis (Li et al., 2011). However, human lung fibroblasts treated with the EP2-receptor agonists (ONO-AE1-259, butaprost) and the EP4-receptor agonist (ONO-AE1-329) showed reduced cell migration (White et al., 2005; Li et al., 2011) probably resulting from inhibition of chemotaxis (Li et al., 2011). Additionally, human fetal lung fibroblast treated concomitantly with AH6809 (EP1/2 and DP1 antagonist) and ONO-AE3-208 (EP4 antagonist) showed a reduced antifibrotic effect of PGE_2_ (Li et al., 2011), whereas the antifibrotic effects of PGE_2_ or the EP2-receptor agonist, butaprost, were absent in mouse lung fibroblasts lacking the EP2 receptor (White et al., 2005), confirming an important role for EP2 receptors in regulating fibroblast proliferation. However, some of the studies do not support the antifibrotic role of EP4 receptor in human fibroblast (Kach et al., 2014), or it was only detectable when both EP2 and EP4 antagonists were used together (Sieber et al., 2018). Furthermore, neither the EP1-receptor antagonist (ONO-8713) nor the EP3-receptor antagonist (ONO-AE3-240) modified the antifibrotic effect of PGE_2_ in human lung fibroblasts (Li et al., 2011).

PGE_2_ mainly promoted an antifibrotic phenotype (inhibition of proliferation and of collagen synthesis; reduced biosynthesis of extracellular matrix proteins) in a G_s_/AC/cAMP-dependent manner by activation of EP2 in humans (Liu et al., 2004). This antifibrotic effect of PGE_2_ mediated by the EP2 receptor was demonstrated in a bleomycin-induced pulmonary fibrosis mouse model (Wei et al., 2014). Indeed, the genetic deletion of EP2 receptor resulted in an excessive fibrotic response (Moore et al., 2005). A protective role of EP2-receptor agonist (butaprost) against mice pulmonary fibrosis was also demonstrated (Moore et al., 2005). It should also be mentioned that some of the above effects can be replicated by EP4 receptors since it was shown in cardiac fibrosis (Lai et al., 2018; Lebender et al., 2018), suggesting some overlapping function of EP2 and EP4 receptors in regulating fibroblast function. Indeed, in a high-throughput screen to assess the therapeutic potential of novel drugs in pulmonary fibrosis, drugs acting at either the EP2 or EP4 receptor were identified as among the most effective agents at inhibiting TGF-*β–*induced myofibroblast differentiation via the SMAD2/3 pathway (Sieber et al., 2018).

When epithelial damage occurs at the bronchial or alveolar level, there is decreased PGE_2_ synthesis and consequently a loss of their capacity to promote the antifibrotic phenotype (Vancheri et al., 2004). The proposed PGE_2_-mediated antifibrotic model (e.g., bronchial tissue remodeling in asthma and pulmonary fibrosis) is based on initial damage with activation of epithelial cells with subsequent activation of inflammatory cells, fibroblasts, endothelial cells, and SMCs. These cells secrete cytokines, chemokines, and growth factors with the double aim of eliminating the “damaging” agent and initiating adaptive tissue repair. Impairment of PGE_2_ production for reasons related to the host and/or to the nature of the damaging agent might lead to persistent inflammation and nonadaptive tissue repair processes (fibrosis/adverse remodeling) (Vancheri et al., 2004).

EP2- and EP4-receptor agonists accelerate the senescence of bronchial fibroblasts, and this could be relevant to bronchial remodeling in COPD. Induced senescence and inflammatory profile were reported in human and mice lung fibroblasts in response to a single exposure to PGE_2_. An induced production of senescence markers (p21 and p53) and proinflammatory mediators (IL-6, CX3CL1, fibroblast growth factor 2, vascular endothelial growth factor, MMP-2) were observed in WT mice fibroblasts but not in knockout mice for p53. Similarly, in control and COPD fibroblasts, increased production of senescence markers and inflammatory mediators were induced by PGE_2_ exposure in vitro (Dagouassat et al., 2013). This effect of PGE_2_ was mediated by EP2/EP4 receptors since it was mimicked by selective agonists like ONO-AE1-259-01 (EP2 agonist) and CAY10598 (EP4 agonist). Indeed, in both COPD or control fibroblasts treated with EP2 and EP4 antagonists together (AH6809 and GW627368X or PF-04418948 and L-161,982), PGE_2_-induced senescence was significantly reduced (Dagouassat et al., 2013). Moreover, EP2- and, to a lesser extent, EP4-receptor expression was found to be enhanced in lung fibroblasts derived from patients with COPD (Dagouassat et al., 2013; Horikiri et al., 2017), suggesting potential sensitization of these two G_s_-coupled receptors in airway remodeling.

### C. Prostaglandin E_2_ and Prostacyclin in Lung Cancer

The link between cigarette smoking and lung cancer is now well established, and COX-2–derived PGE_2_ has a well known role in cancer, stimulating tumor-associated angiogenesis, cell invasiveness, and cell proliferation as well as inhibiting apoptosis (Huang and Chen, 2011). PGE_2_ and an EP4-receptor agonist, PGE1-OH, are known to promote human A549 lung cancer–cell migration (Kim et al., 2010; Hirata and Narumiya, 2012).

Specific EP3- or EP4-receptor agonists (ONO-AE-248 and ONO-AE1-329) stimulated CXCL12 expression by mice fibroblasts in vitro, whereas EP3- or EP4-receptor deficiency reduced stromal expression of CXCL12/ C-X-C motif chemokine receptor 4 in mice implanted with Lewis lung carcinoma cells (Katoh et al., 2010). The EP2 receptor was found in human nonsmall cell lung carcinoma cell lines (H1838, H2106) by Western blot and real-time PCR and is also responsible for lung tumorigenesis (Han and Roman, 2004). In these cell lines, treatment with EP2 agonists [butaprost, 16,16-dimethyl-PGE_2_ (EP3 > EP2/EP4 agonist)] enhanced cell proliferation. Additionally, treating these cells with PPAR-*γ* ligands for 24 hours had an inhibitory effect on EP2-receptor expression (Han and Roman, 2004). Similarly, a protumorigenic effect of PGE_2_ was also mediated by EP2 receptor in mice since EP2 receptor-depleted mice were protected against lung tumorigenesis (Keith et al., 2006). After 20 weeks of exposure to butylated hydroxytoluene (a tobacco carcinogen) and to 3-methylcholanthrene (a food additive), knockout mice for EP2 receptor presented fewer lung tumors compared with wild-type mice. However, the proinflammatory role of PGE_2_ was maintained even in absence of EP2 (Keith et al., 2006).

In contrast to EP2 agonists, IP agonists like iloprost were shown to inhibit human nonsmall cell lung cancer growth (Tennis et al., 2010). Likewise, administration of a PGI_2_ analog, beraprost, reduced tumor metastasis in a mouse lung metastasis model using Lewis lung carcinoma cells (Minami et al., 2015). This contrasts with another study, which showed that a second-generation PGI_2_ analog, treprostinil, which has potent IP and EP2 affinity, failed to prevent tumors in a mouse lung adenocarcinoma model using JF32 cells (Dwyer-Nield et al., 2017). Despite this, and the known role of EP2 receptor in lung cancer through its activation of the epidermal growth factor (Huang and Chen, 2011), there is no evidence so far that this analog increases the incidence of cancer, including in the lung. This may result from treprostinil’s known activation of PPARs and their generally antitumor effects (Clapp and Gurung, 2015).

Lung cancer induced by tobacco carcinogens could be inhibited by PGI_2_ in both human and mice models. In human bronchial epithelial cells (HBECs) exposed to cigarette smoke condensate (CSC), the COX-2 expression and PGI_2_ synthesis are increased after 4 weeks of exposure, whereas [PPAR-*γ*, 15-PGDH, and carboxylesterase 1 (CES1)] mRNA expressions are downregulated after 16 weeks of exposure. The treatment with iloprost of these HBECs already exposed to CSC for 4 weeks reversed the effect of CSC on PPAR-*γ* and CES1 expression (New et al., 2018). Similarly, mice receiving urethane (tobacco carcinogen) during 20 weeks developed multiple adenomas, and the same gene (PPAR-*γ*, 15-PGDH, and CES1) expressions were downregulated while COX-2 expression increased (New et al., 2018). In this study, when transgenic mice for PGIS were used, they were protected against cancer by high levels of produced PGI_2_. However, the authors assume that PGI_2_/iloprost effects in HBEC or mice are PPAR-*γ* mediated, and the IP-receptor role remains to be explored.

## VII. Upper and Lower Urinary Tract

Prostanoids participate in controlling the relaxation and contraction of urinary bladder and urethra, thus affecting voiding and micturition, respectively (Andersson et al., 2018). They also play an important role in various parts of the kidney and ureters, thus controlling salt and water retention as well as renin secretion in human and experimental animals (Grantham and Orloff, 1968; Sonnenburg and Smith, 1988; Hao and Breyer, 2008).

### A. Urinary Bladder

Biopsies of human urinary bladder mucosa were shown to release eicosanoids in the following order: PGI_2_, PGE_2_, prostaglandin F_2_ *α* (PGF_2*α*_), and TxA_2_. The amounts of eicosanoids released were similar to those reported for the rat urinary bladder (Jeremy et al., 1987b). EP1 receptor is gaining much importance being involved in initiation of the micturition reflex (Lee et al., 2007). However, blocking EP1 receptor (using PF2907617) caused a rightward shift of the PGE2 concentration-response curve in the rat bladder but not in the human one, and the same was observed using CJ24979, a selective EP3-receptor antagonist. It seems that although PGE_2_ is of equal importance in both human and rat bladder, difference in receptors that mediate its effects may exist. In a monkey model, the dual EP2/EP3 agonist ONO-8055 dose-dependently improved voiding dysfunction of underactive bladder (Kinoshita et al., 2018).

Recent reports show that PGE_2_ is involved in the development of bladder overactivity in both human (Rahnama’i et al., 2013) and rats (Wada et al., 2018). Moreover, increased PGE_2_ production and mRNA expression of EP1 and EP2 receptors were observed in the bladder of patients with interstitial cystitis (Wada et al., 2015). The same was reported in an equivalent rat model (Zhang et al., 2016b). Deficiency in PGI_2_ production was also implicated in the development of idiopathic primary detrusor instability in human (Bergman et al., 1991). Consistent with this finding, PGI_2_ was proven to facilitate the micturition reflex in rats (Cefalu et al., 2007). Elevated PGE_2_ levels were observed in bladder carcinogenesis in human and rats, respectively (Eschwège et al., 2003; Shi et al., 2006). In invasive bladder cancer in mice, mRNA levels for EP2 and EP4 receptors were increased by 2–3-fold after 4–8 weeks from administration of *N*-butyl-*N*-(4-hydroxybutyl)-nitrosamine, a carcinogen inducing bladder cancer. In addition, expression of COX-2 was also upregulated by 3–4-fold while expression of 15-PGDH was downregulated by 50%–80% (Taylor et al., 2009). In human, inhibition of PGE_2_ formation plays a major role in tumor escape from immune system during bladder cancer progression, as reported by Prima et al. (2017). Moreover, a clinical study favored EP1-receptor antagonist ONO-8539 as a potential treatment of non-neurogenic overactive bladder syndrome (Chapple et al., 2014).

### B. Urethra

As for the urethra, it is established that PGE_2_ relaxes contracted urethral muscles, which seems complementary for bladder emptying. This effect has been proven in humans (Klarskov et al., 1983) as well as in guinea pigs (Finkbeiner and Bissada, 1981), in which the effect of PGE_2_ was completely blocked by SC19220, a supposed EP1 antagonist (Finkbeiner and Bissada, 1981). No data are reported in other rodents.

### C. Kidney

Literature highlights PGE_2_ and PGI_2_ as the two main prostanoids of functional importance in kidney. Rat glomeruli in the cortex produce mainly PGE_2_ and less PGI_2_, but human glomeruli synthesize mainly PGI_2_ and some PGE_2_ (Schlondorff, 1986; Bonvalet et al., 1987). Colocalization of mPGES-1, whose deletion affects PGE_2_ levels in urine (Li et al., 2017d), and COX-2 in rat cortical thick ascending limb and medullary interstitial cells suggests that mPGES1 is functionally coupled to COX-2. In the collecting ducts, on the other hand, mPGES-1 is coupled to COX-1 (Hao and Breyer, 2008). This data has been confirmed in rodents but not in humans. This necessitates further studies to explore the relationship between selective COX inhibition and PGE_2_ production in the collecting ducts, which greatly control water permeability. On the other hand, abundant expression of PGIS was observed in the nephrogenic cortex in humans, and in situ hybridization revealed an identical pattern in mice (Klein et al., 2015).

The EP and IP receptors have all been detected in the kidney and in renal vessels, and their species-dependent vascular presence and roles are discussed above in the sections *III. A. 1. Vascular Tone Regulation* and *III. B. 1. Hypertension*. Most of these receptors are associated with specific renal functions (Hao and Breyer, 2008); however, very few studies found EP2-receptor expression in (nonvascular) kidney except one work with RT-PCR in rat (Jensen et al., 2001). EP1-receptor mRNA expression appears to be restricted to the collecting duct in both mouse and human (Guan et al., 1998). EP3-receptor mRNA are strongly expressed in human thick ascending limb and in outer and cortical collecting ducts (Breyer et al., 1996; Hao and Breyer, 2008). However, no change was detected in urine osmolality and volume in EP3 knockout mice (Fleming et al., 1998); these results suggest that EP3 receptors could have different roles in human and mouse kidneys. Further investigations should explore this discrepancy. Photomicrographs of EP4-receptor mRNA and protein were observed in human glomeruli (Breyer et al., 1996; Hao and Breyer, 2008; Thieme et al., 2017). EP4 receptors were more abundant in rodents and were found in glomeruli, distal convoluted tubule, and cortical collecting duct (Jensen et al., 2001; Thieme et al., 2017). IP receptors in human and rodents were localized by different techniques (Western blot and mRNA in situ hybridization) in glomeruli (cortex), the medulla, distal tubules, and collecting ducts (nephron-collecting ducts where they are coupled to inhibition of cAMP production) (Kömhoff et al., 1998; Nasrallah et al., 2001).

When PGI_2_ release is genetically abrogated, mice become hypertensive and show fibrosis and vascular remodeling in the kidney (Yokoyama et al., 2002). In an attempt to sustain renal blood flow and thereby prevent hypoxic damage to the tubulointerstitium, the orally active prostacyclin analog beraprost was tested in patients with chronic kidney disease and showed some positive effects regarding the decline of kidney function (Koyama et al., 2015). Similarly, iloprost prevented contrast media–induced nephropathy in patients with renal dysfunction undergoing coronary angiography or intervention (Spargias et al., 2009).

An EP4 receptor–derived peptide, which acts as a negative allosteric modulator, restored renal function in models of acute renal failure (Leduc et al., 2013), and EP4 antagonists prevented inflammation and renal impairment in a mouse model of acute glomerulonephritis (Aringer et al., 2018).

#### 1. Water and Salt Regulation

It is well known that PGE_2_ is the most important prostanoid in regulating water and solutes balance. Although COX-1 is constitutively expressed in the kidney, mice deficient in COX-1 appear to be healthy with no obvious renal defects. In contrast, COX-2 seems to play a more important role in regulating renal water transport (Li et al., 2017d). In rats, the EP3 receptor is reported to regulate water excretion in response to high salt intake; it decreases collecting duct-water permeability and increases water excretion. High-salt treatment increased COX-2–dependent PGE_2_ production when the EP3 receptor was blocked by L-798106 in the thick ascending limb, whereas urine output was decreased when the EP3 receptor was activated by sulprostone (EP3 > EP1 agonist) (Hao et al., 2016). EP2 and EP4 receptors are reported to bypass vasopressin signaling and increase water reabsorption (Olesen and Fenton, 2013). In rodents, it was also shown that PGE_2_ inhibits AC and NaCl reabsorption in thick ascending loop of Henle (Schlondorff and Ardaillou, 1986). By radioligand membrane binding and autoradiography, the localization of [^3^H]PGE_2_ was demonstrated in proximal tubule as well as the glomeruli of human kidney, a distribution that is in accordance with the assumed site of action for the salt and water regulatory function of PGE_2_ (Eriksson et al., 1990). However, mechanistic studies have been confined to animal experiments, and these findings need to be examined in humans.

#### 2. Renin Release

PGE_2_ and PGI_2_ stimulate renin secretion and renin gene expression by activating cAMP formation in human juxtaglomerular cells (Wagner et al., 1998) probably through activation of the EP4 receptor (Kaminska et al., 2014). The same mechanism was demonstrated in mice (Jensen et al., 1996; Wang et al., 2016a).

According to Hao and Breyer (2007), in nephrotic syndrome, maintenance of normal renal function in human becomes dependent on COX-2–derived prostanoids, particularly PGE_2_ and PGI_2_, with an active participation of EP4, EP2, and IP receptors that mediate their vasodilator effect. In an equivalent rat model, a selective EP4 antagonist (L-161982) exacerbated proteinuria and glomerular cell apoptosis (Aoudjit et al., 2006). More specifically, and in diabetic nephropathy, the involvement of EP1 (Kennedy et al., 2007) and EP3 receptors (Hassouneh et al., 2016) in mediating disease progression has demonstrated that arginine vasopressin–mediated water reabsorption was reduced in sulprostone-treated or EP_1_^−/−^ rats. These pathophysiologic effects were not proven in human. On the other hand, elevation of COX-2 and PGE_2_ expression is the main feature of renal cell carcinoma in both human (Kaminska et al., 2014) and rat (Rehman et al., 2013).

### D. Ureters

The EP1 receptor, rather than EP2–4, was highly expressed in human ureters (Oll et al., 2012). PGE_2_ increases contractility in obstructed human ureters and relaxes nonobstructed ureters (Lowry et al., 2005). The expression of EP2 and EP4 receptors as well as contracting TP and EP1 receptors were reported in rat ureters (Nørregaard et al., 2006).

## VIII. Reproductive System

Prostanoids affect the contractility of different genital tract organs, including prostate, testicular capsule, epididymis, vas deferens, and corpus cavernosum in males as well as uterus and ovary in females. These lipids also influence the transportation of spermatozoa and the ovulation process, suggesting that they may have important roles in both male and female reproductive functions (Hafs et al., 1974).

### A. Vas Deferens

A previous study suggested that intrinsic PG contents in rat and mouse vas deferens are higher than the other reproductive tissues and may partially regulate sperm transportation (Badr et al., 1975). In mouse vas deferens, the epithelium is the most likely site for PG production (Marshburn et al., 1989). However, in human, only a small part of the seminal PGs might originate from human vas deferens, whereas the seminal glands are likely to be a main source of the PGs in human semen (Gerozissis et al., 1982). PGs affect the maturation and function of human sperm. For instance, PGE_2_ is suggested to stimulate the motility of human sperm (Didolkar and Roychowdhury, 1980). It has also been reported that sperm function is improved when human spermatozoa are incubated with low physiologic level of PGE_2_ (Rios et al., 2016). These reports with different levels of PGs production between human and rodent vas deferens require further investigations.

The rat vas deferens releases predominantly PGE_2_ and PGF_2*α*_ under basal conditions in vitro. Indeed, incubation of this tissue with AA results in an increase in PGE_2_ production (Gerozissis and Dray, 1983). In contrast, the human vas deferens synthesizes PGs only when AA is supplied exogenously (Patra et al., 1990). Interestingly, iloprost did not show any effects on adrenergic neurotransmission in human vas deferens, suggesting less contribution of PGI_2_ to this system (Holmquist et al., 1991).

There is no sufficient data showing the PG receptors expressed in vas deferens. However, based on the previous reports, PGE_2_ released from the epithelium of rat vas deferens upon ATP stimulation might act on smooth muscle, possibly via EP2/EP4 receptors, and the cAMP-dependent pathway leading to the activation of K^+^ channels, membrane hyperpolarization, and hence the inhibition of smooth muscle contraction (Ruan et al., 2008).

Although PGs are not sufficiently synthesized under basal conditions, these lipids may affect contractility of human vas deferens. In isolated human vas deferens, both PGE_1_ and PGE_2_ inhibited adrenergic responses by a prejunctional mechanism that involves the activation of large-conductance Ca^2+^-activated K^+^ channels and Na^+^/K^+^-ATPase (Medina et al., 2011). On the other hand, it was reported that PGE_2_ itself induces contractile response in the rat vas deferens (Amadi et al., 1999). Meanwhile, another study reported that in rat and human vas deferens the endogenously synthesized PGs have no effects on contractility (Patra et al., 1990). More focused research should be directed toward investigating the effect of PGE_2_ on premature ejaculation in human and rodents.

### B. Prostate

Several types of PGs are normally synthesized in human and rodent prostate and play a major role in prostate cancer development. It is known that the name “prostaglandin” is derived from prostate gland, as PGs were first discovered in this gland and in seminal fluid (von Euler, 1936; Bergstroem et al., 1963). Human prostate strips contain thromboxane B_2_, a stable metabolite of TxA_2_ (Strittmatter et al., 2011). PGI_2_ synthase is also expressed in human prostate (Miyata et al., 1994). In rat prostate, PGE_2_ is reported to inhibit electrical field-induced contraction in a concentration-dependent fashion (Tokanovic et al., 2007).

PGE receptors are expressed in healthy prostate tissue as well as in prostate cancer. For instance, EP3 receptor is expressed in human healthy prostate (Kotani et al., 1995), and its expression level is decreased, whereas the expression levels of EP2 and EP4 receptors are increased in human prostate cancer (Huang et al., 2013). The inhibitory effect of PGE_2_ on rat prostate contractility may be mediated via EP2 but not EP3 since EP3 > EP1 agonist, sulprostone, failed to mimic the PGE_2_-mediated action (Tokanovic et al., 2010).

AA is found at a low level in tumor specimens obtained from radical prostatectomy, presumably because of an increase in its metabolic conversion into PGE_2_ (Chaudry et al., 1994). PGE_2_ induces the production of vascular endothelial growth factor in prostate cancer cells through EP2 receptor-cAMP pathway, which in turn promotes angiogenesis (Wang and Klein, 2007).

In this regard, inhibition of PGE_2_ synthesis may exert an antitumor effect. It was demonstrated in both human and rat prostate cancer that metformin is able to inhibit migration of prostate cancer cells and tumor invasion by decreasing COX-2 level and PGE_2_ production (Tong et al., 2017).

PGI_2_ is the major component in both benign and malignant prostate tissues, as shown by using mass spectrometric analysis. Because its plasma level is elevated in patients with prostate carcinoma, 6-oxo-PGF_1*α*_, a metabolite of PGI_2_, may serve as a reliable diagnostic marker for prostate cancer (Khan et al., 1982).

### C. Corpus Cavernosum

Prostanoids play a significant role in erectile process. PGE_1_ is highly effective in management of erectile dysfunction via induction of corpus cavernosal relaxation (Hanchanale and Eardley, 2014). PGs may also inhibit platelet aggregation, modulate collagen synthesis, and regulate fibrosis in corpus cavernosal tissues (Moreland et al., 1995).

Human corpus cavernosum produces all major PGs, including PGE_2_, PGD_2_, PGF_2*α*_, PGI_2_, and TxA_2_ (Khan et al., 1999), and expresses their receptors, like EP1, EP2, EP3-type I, EP3-type II, EP4, and DP (Brugger et al., 2008). There is no sufficient data showing their expression in rodent corpus cavernosal tissues.

In contrast to PGF_2*α*_ and TxA_2_, which induce human penile contraction, PGE_1_ and PGE_2_ have been shown to induce corpus cavernosal relaxation via EP2 and EP4 receptors and subsequently stimulate AC/cAMP pathway, resulting in stimulation of penile erection (Khan et al., 1999). It was suggested that PGE_1_-induced relaxation of human corpus cavernosal smooth muscle is related to activation of the large conductance Ca^2+^-activated K^+^ channels, resulting in hyperpolarization (Lee et al., 1999). Moreover, both PGE_1_ and PGE_2_ are reported to inhibit noradrenaline release in human penile tissue, suggesting that PG receptors may affect penile erection by modulating the presynaptic release of neurotransmitters (Minhas et al., 2001). Interestingly, cholinergic stimulation of human corpus cavernosum induces the release of PGI_2_, which may take part in maintenance of erection (Jeremy et al., 1986).

Similarly, PGE_1_ stimulates the relaxation of phenylephrine-precontracted isolated rat corpus cavernosum, and both an EP_4_ agonist and iloprost also exert relaxant actions, suggesting pivotal roles of EP4 and IP receptors in penile function in rat (Bassiouni et al., 2019). EP4/IP-receptor signaling presumably elicits a decrease in the intracellular Ca^2+^ level in corpus cavernosal smooth muscles via the AC/cAMP pathway, resulting in corpus cavernosal relaxation (Ricciotti and FitzGerald, 2011).

PG actions in penile erection are mediated by not only relaxation of corpus cavernosum but also by tone regulation of penile arteries. PGE_1_ induces relaxation of penile resistance arteries, resulting in an increase in the blood flow to the corpus cavernosal muscles and induction of penile erection (Ruiz Rubio et al., 2004). PGI_2_ also induces vasodilation of human penile artery (Khan et al., 1999).

Although PGE_1_ is considered an effective therapeutic option for management of erectile dysfunction, PGs may be involved in some other penile pathologic conditions. For instance, PGE synthase-1 is reported to be overexpressed in human penile intraepithelial neoplasia and carcinoma (Golijanin et al., 2004).

### D. Uterus

Endogenous PGs are known to have an important role in the normal uterine motility and its regulation during the menstrual cycle. In human, PGE_2_, PGF_2*α*_, and PGI_2_ were reported to be released during different phases (Jensen et al., 1987). However, in rats, PGF_2*α*_, PGE_2_, and PGD_2_ were shown to be present in pseudopregnant rat uterus (Fenwick et al., 1977).

Multiple EP receptors are expressed in human uterine tissue and play roles during pregnancy and labor. Depending on their signaling pathway, EP receptors may promote or inhibit the uterine smooth muscle contraction (Astle et al., 2005). EP3 is suggested to be the predominant receptor responsible for PGE_2_-induced contraction of pregnant human myometrium during term labor (Arulkumaran et al., 2012). EP3 expression is reported to be higher in the upper segment of the uterus, whereas EP2 is more expressed in the lower segment (Astle et al., 2005). On the other hand, there are only a few studies showing the dynamic changes in uterine expression of PG receptors in rodents. PGE_2_, 17-phenyl trinor PGE_2_ (EP1 agonist), sulprostone (EP3 > EP1 agonist), and misoprostol (EP2/EP3/EP4 agonist) selectively contract pregnant guinea-pig myometrium, in which the EP3-receptor activation is more likely involved since mRNA of EP1 receptor was not found in this tissue (Terry et al., 2008). Uterine expression levels of EP1 and EP3 are upregulated in response to estradiol and progesterone in ovariectomized rat (Blesson et al., 2012). Another study showed that EP2 expression in the myometrium is elevated during preterm labor (Molnár and Hertelendy, 1990). Activation of EP2 is responsible for the relaxant effect of PGE_2_ on pregnant rat uterine (Khan et al., 2008).

During human menstrual cycle, PGE_2_ induces vasodilation of the endometrial vessels, and PGI_2_ elicits relaxation of the smooth muscle, vasodilation of the myometrial vessels, and inhibition of thrombocyte aggregation (Jensen et al., 1987). Intrauterine or oral administration of PGE_2_ was also shown to inhibit uterine contractility during active menstrual bleeding in both normal and dysmenorrheal women (Bygdeman et al., 1979). During pregnancy, PGI_2_ was reported to maintain the uterus in quiescent state during early pregnancy via inhibition of its contractile activity (Patel and Challis, 2001). However, during labor, the concentrations of PGE_2_ and PGF_2*α*_ are augmented in amniotic fluid, and their metabolites are detectable in maternal plasma and urine (Novy and Liggins, 1980). Moreover, PGE_2_ has been shown to stimulate the fundal myometrium in vitro before and during labor (Wikland et al., 1984). When comparing the oxytocic activities of different PGs in pregnant rats, PGE_2_ and PGI_2_ elicit the most potent uterine contraction in vitro, but PGE_2_ exerts more potent oxytocic activity (Williams et al., 1979).

Therapeutically, different clinical studies showed that PGE_2_, probably by EP3 activation, can be used as potent oxytocic agent (Gillett, 1972). For examples, PGE_2_ is used in the form of vaginal suppositories as oxytocic for induction of abortion during the midtrimester or fetal demise during the third trimester of pregnancy (Wiley et al., 1989). Misoprostol, EP2/EP3/EP4 agonist, is used off-label for abortion and induction of labor (Allen and O'Brien, 2009).

Pathologically, EP3 receptor is an important biomarker for endometrial cancer, and blockade of EP3 activation exerts an antitumor effect; EP3 receptor may serve as a possible therapeutic target for endometrial cancer patients (Zhu et al., 2017). Furthermore, PGE_2_ has been shown to stimulate proangiogenic factors in endometrial adenocarcinoma cells probably via the EP2 receptor, whose expression is elevated in tumor cells (Sales et al., 2004). Additionally, PGE_2_ may be involved in the pathogenesis of cervical cancer because it promotes angiogenesis, proliferation, and invasiveness of tumor cells and inhibits the antitumor immune responses (Fitzgerald et al., 2012).

### E. Gonads

Previous studies reported the production of PGE_2_, PGF_2*α*_, and PGI_2_ as well as expression of IP, EP1–4, and FP receptors in sertoli cells, the somatic cells of the testis, in both prepubertal and adult rats (Cooper and Carpenter, 1987; Ishikawa and Morris, 2006). In addition, Walch et al. (2003) demonstrated that multiple EPs as well as FP receptors are expressed in stem cells of rat Leydig cells and PGE_2_-EP1 and PGF_2*α*_-FP pathways stimulate IL-1*β* expression in these cells. PGE_2_ may be involved in homeostatic regulation of human testicular peritubular cell function (Rey-Ares et al., 2018). Moreover, expression of COX-2 and production of PGE_2_ were detected in rat spermatogenic cells (Winnall et al., 2007).

Functionally, PGE_2_ and PGF_2*α*_ were reported to decrease the plasma levels of testosterone in male rat (Saksena et al., 1973). Similarly, intratesticular administration of PGE_2_ or PGD_2_ elicited a significant decrease in the levels of testosterone in rat testicular tissues (Yamada et al., 1985). Moreover, expression of COX-1 and COX-2 isozymes is induced in human testicular cancer tissue, suggesting a potential role of PGs in the pathogenesis of testicular cancer (Hase et al., 2003).

PGs have been shown to play a remarkable role in the female reproductive system. PGE_1_, PGE_2_, and PGF_2*α*_ are detectable in human ovarian follicular fluid (Pier et al., 2018). Functionally, PGE_2_ seems to be important for ovulation because it is mainly synthesized within the follicle and acts as an essential mediator in the gonadotropin-induced ovulation (Goldberg and Ramwell, 1975). In rat, upon gonadotropin stimulation, COX-2 is induced in follicular cells, resulting in the release of a large amount of PGE_2_ into the follicular fluid (Brown and Poyser, 1984). Similarly, expression of the EP2 receptor is induced in cumulus cells, a type of granulosa cell surrounding the oocyte, and mice lacking the EP2 receptor exhibit reduced ovulation and impaired fertilization (Hizaki et al., 1999). These findings need to be further examined in humans.

PGE_2_ also plays a key role in the growth and progression of ovarian cancer in human, which is supported by the reports demonstrating the elevated expression of EP1/EP2 receptors in epithelial ovarian cancer (Rask et al., 2006). On the other hand, PGI_2_ inhibits invasion of human ovarian cancer cells in an IP receptor–dependent manner (Ahn et al., 2018).

## IX. Gastrointestinal Tract

The role of prostaglandins in gastrointestinal tract and liver physiology has been distinctly established in rodent models and human using in vivo and in vitro experiments. In the last decades, the aim of pharmacological studies was to determine how EP and IP receptors are acting in inflammatory process in gut, stomach, and liver.

### A. Prostaglandin E_2_ and Prostacyclin in Stomach and Intestine

#### 1. Mucosal Protection

In rodents, endogenous PGI_2_ and PGE_2_ are constitutively produced in the stomach through constitutive COX-1. These two PGs reduce stomach acid secretion, activate mucosal blood flow, and facilitate the release of viscous mucus (Amagase et al., 2014; Takeuchi and Amagase, 2018). In human, the protective effect of PGE_2_ analogs, such as 16,16-dimethyl PGE_2_ (EP3 > EP2/EP4 agonist) against gastric-acid secretion and gastric-ulcer formation, was shown in the late 1960s (Robert et al., 1968, 1974). Further pharmacological results obtained in human and dogs proved the involvement of the EP3 receptor in inhibition of gastric-acid secretion (Robert et al., 1974; Tsai et al., 1995). In mice, the inhibitory action of prostaglandins (i.e., PGE_2_ and PGI_2_) on gastric-acid production in the damaged stomach by taurocholate sodium is mediated by activation of EP3 and IP receptors (Nishio et al., 2007; Takeuchi and Amagase, 2018). Similarly, EP3-receptor activation in rat is responsible for a reduced production of gastric acid induced by pentagastrin/histamine stimulations (Kato et al., 2005). PGI_2_ and PGE_2_ play crucial roles in gastric mucosal defense during induced cold-restraint stress through IP and EP4, respectively. Gastric lesions induced by 18 hours of cold-restraint stress are significantly increased in IP knockout mice compared with WT mice. In WT mice, pretreatment with iloprost and indomethacin in combination prevented gastric lesions caused by cold-restraint stress (Amagase et al., 2014).

NSAIDs are well known to be responsible for gastric lesions and stomach injuries, and PGE_2_ can prevent and reverse these effects (Johansson et al., 1980). Gastric damage induced by indomethacin or by HCl/ethanol in rat could be prevented by PGE_2_ or EP1-receptor agonists [17-phenyl trinor PGE_2_ (EP1 agonist) and sulprostone (EP3 > EP1 agonist)], and this protection disappears in EP1 knockout mice (Araki et al., 2000; Suzuki et al., 2001; Takeuchi, 2012, 2014). This EP1-mediated effect is attributed to the inhibition of gastric hypermotility induced by NSAIDs (Takeuchi et al., 1986); most of these experiments have been done with rodents, so these receptor subtype roles in the gastrointestinal tract need to be confirmed in human. On the other hand, the clinical perspective to use EP1-receptor agonists should be limited to treat NSAID-induced damage, to stimulate bicarbonate production in the stomach, and to reduce acid reflux in esophagus (Takeuchi et al., 1997; Suzuki et al., 2001; Takeuchi and Amagase, 2018). Surprisingly, in a recent clinical study (20 patients), the EP1 antagonist ONO-8539 had a positive effect on acid-induced heartburn in healthy male subjects with gastroesophageal reflux disease (Kondo et al., 2017). Because this antagonist would probably act on symptoms or sensorial responses and not on the endogenous cause of acid reflux in esophagus, further investigations are necessary.

Mucus and bicarbonate secretions by epithelial cells are further physiologic mechanisms involved in preventing/healing gastric lesions. Indomethacin-induced gastric-mucosa lesions could be prevented by the administration of misoprostol (EP2/EP3/EP4 agonist), resulting in a lower edema average in gastric mucosa (Cavallini et al., 2006). Furthermore, administration of an EP4-selective agonist also significantly reduced indomethacin-induced apoptosis of human gastric-mucous epithelial cells (Jiang et al., 2009). These results suggest that EP4-activating reagents may be used to prevent NSAID-induced ulcers by maintaining mucous epithelial-cell survival.

Globally, gastrointestinal cytoprotection induced by selective EP3/EP4-receptor agonists could be very promising. On the one hand, these agonists may reduce gastric-acid secretion and mucosal inflammation (e.g., in colitis), and on the other hand, they increase mucus and bicarbonate productions (Robert et al., 1974; Takeuchi et al., 1997; Kato et al., 2005; Larsen et al., 2005; Takeuchi and Amagase, 2018). For this reason, misoprostol (*Cytotec*, the EP2/EP3/EP4 agonist), with its mucoprotective and antiacid properties, is already an effective treatment of gastrointestinal injury in clinic, which has been shown to be more effective than omeprazole (a proton pump inhibitor) (Taha et al., 2018; Kim et al., 2019). The effect of misoprostol could be probably through activation of the EP4 receptor. PGE_2_-EP4 signaling has also a protective role in colon mucosal barrier in human and murine models. In EP4 knockout mice (EP4^−^/^−^) treated with dextran sodium sulfate (DSS), a loss of the colon epithelial barrier and the accumulation of neutrophils and CD4+ T cells in the colon were observed (Kabashima et al., 2002). In WT mice, the use of selective EP4-receptor antagonist (ONO-AE3-208) led to the development of severe DSS-induced colitis (Kabashima et al., 2002). However, the administration of selective EP4-receptor agonist (ONO-AE-734) to wild-type mice suppressed DSS-induced colitis (Kabashima et al., 2002). In human, the use of selective EP4-receptor agonist rivenprost (ONO-4819) in patients with mild-moderate ulcerative colitis significantly improved histologic scores and reduced the disease activity index after 2 weeks of therapy (Nakase et al., 2010).

#### 2. Gastrointestinal Motility and Muscular Tone

Gastric cytoprotection and lesions are also associated with gastrointestinal motility (contraction/frequency of contraction) (Suzuki et al., 2001). In vivo, inhibition of PGE_2_ and PGI_2_ synthesis by indomethacin and many other NSAIDs in rat stomach or intestine cause increased motility (Takeuchi et al., 1986; Takeuchi, 2012). This increase is reversed by exogenous PGE_2_ and by selective EP1 agonist and EP4 agonists when motility is measured in rat stomach and intestine, respectively (Suzuki et al., 2001; Kunikata et al., 2002; Takeuchi and Amagase, 2018). The mechanism associated with this NSAID increased motility in vivo is still unknown, despite that it was suggested to a *central vagal* stimulation (Yokotani et al., 1996). In contrast, many other in vitro studies using isolated gastrointestinal preparations (longitudinal muscle) have shown a contractile role for exogenous PGE_2_ in human stomach and colon (Bassil et al., 2008; Fairbrother et al., 2011), rat gastric fundus and colon (Abraham et al., 1980; Sametz et al., 2000; Bassil et al., 2008; Iizuka et al., 2014), guinea-pig ileum and fundus (Coleman and Kennedy, 1985; Sametz et al., 2000), and mice ileum (Fairbrother et al., 2011). Numerous in vitro pharmacological studies using PGE_2_ or selective agonists (e.g., ONO-DI-004 for EP1, ONO-AE-248 for EP3, sulprostone for EP3 > EP1) and antagonists (e.g., EP1A, ONO-8713, SC51089, and SC19220 for EP1, ONO-AE3-240 and L-798106 for EP3) have determined that EP1-receptor activation is responsible for contractions of human longitudinal muscle, whereas in rodent gastrointestinal muscles it is EP1- and EP3-receptor activations that are responsible (Coleman and Kennedy, 1985; Sametz et al., 2000; Fairbrother et al., 2011; Iizuka et al., 2014). This difference of effects induced by PGE_2_ (or mimetics) on motility versus contraction of gastrointestinal tract in vitro, dependent or independent of neuronal (central) component, and the difference between species need more experiments.

One possible clinical application of this EP1 receptor on motility in humans is illustrated by lubiprostone (a PGE_1_ derivate and chloride channel type 2 channel opener) as a treatment of constipation. On rat and human stomach longitudinal muscle and also on mouse intestinal circular muscle, lubiprostone has a contractile effect mediated by activation of EP1 receptor. However, EP4-receptor antagonists reduced the contractile action of lubiprostone on circular colon muscle in rat and human (Bassil et al., 2008; Chan and Mashimo, 2013).

In human, the pronounced contractile effect of PGI_2_ analogs on isolated gastric smooth muscle may contribute to explaining abdominal pain and cramping associated with the use of these compounds in clinic. However, selexipag and its active metabolite MRE-269 (highly selective IP agonist) had few gastric side effects (Morrison et al., 2010). In contrast, other PGI_2_ analogs, like iloprost and beraprost that have some affinity for EP3 and EP1 receptors, induced dose-dependent contraction of rat gastric fundus. Furthermore, the contraction to iloprost and beraprost was inhibited by an EP3-receptor antagonist ((2E)-3-(3Ј,4Ј-dichlorobiphenyl-2-yl)-N-(2-thienylsulfonyl)acrylamide) and EP1-receptor antagonists (SC19220 and SC51322), suggesting that EP1 receptors contributed to contraction of gastric fundus to iloprost and beraprost (Morrison et al., 2010).

### B. Prostaglandin E_2_ in Pancreas

The production of PGE_2_ in Langerhans islets can be induced by systemic inflammation and hyperglycemia (Carboneau et al., 2017). Blockade of EP3 by DG-041 (an EP3 antagonist) increased proliferation of human and young (but not old) mouse *β*-cell proliferation, whereas either activation of EP4 (by its agonist CAY10598) or blockade of EP4 (by its antagonist L-161,982) had few effects on both human and mouse *β*-cell proliferation. Moreover, EP3 blockade (with DG-041 or L-798106) or EP4 activation prevented palmitate or cytokine (TNF-*α*, IFN-*γ*, and IL-1*β*)-induced *β* cell death in both human and mouse islets, indicating that EP3 and EP4 reciprocally regulate *β*-cell survival (Carboneau et al., 2017; Amior et al., 2019). In addition, human pancreatic stellate cells isolated from pancreatic adenocarcinoma samples and incubated with a selective EP4-receptor antagonist (ONO-AE3-208) showed a reduced cell migration compared with nontreated cells. These findings suggested that PGE_2_ has a profibrotic effect mediated via the EP4 receptor (Charo et al., 2013). Thus, the EP4 receptor may be a potential target in pancreatic cancer therapy.

### C. Prostaglandin E_2_ and Prostacyclin in Liver

The protective role of PGE_2_ after liver injury has been reported in both rodent and human livers. In mice liver, the protective role of PGE_2_ is mediated by the EP4 receptor as an EP4 agonist dose-dependently protected against ischemia/reperfusion-induced liver injury (Kuzumoto et al., 2005). Injection of a high dose (100 μg/kg) of an EP4 agonist, ONO-AE1-329, significantly reduced the level of alanine aminotransferase in serum, a marker of liver function, when compared with the treatment with a low dose (30 μg/kg) of ONO-AE1-329 or vehicle control (Kuzumoto et al., 2005). Thus, PGE_2_-EP4 signaling protects against hepatocyte damage after ischemic/reperfusion injury via EP4. A similar protective effect of EP4 (using its agonist CAY10598) on liver ischemia/reperfusion-induced, mitochondria-associated cell injury was found in the rat (Cai et al., 2020).

The protective effects of the PGE_2_-EP4 pathway on hepatocytes may also be due to reduced expression of proinflammatory cytokines (e.g., IL-1*β*, TNF-*α*, and IFN-*γ*) and enhanced expression of anti-inflammatory cytokines (e.g., IL-10) (Kuzumoto et al., 2005). For example, PGI_2_ and PGE_2_ inhibit hepatocellular necrosis through downregulation of TNF-*α* and IFN-*γ* in mice liver (Yin et al., 2007). In contrast, Sui and colleagues (2018) reported that PGE_2_ and PGI_2_ increased hepatic stellate cell proliferation and activity via PKC in LX-2 human hepatic stellate cell line, and enhanced secretion of proinflammatory cytokine TGF-*β*1 and PDGF was observed compared with the control. Treatment of hepatocarcinoma cells with a selective EP1 agonist (17-phenyl trinor PGE_2_) induced cell invasion via the upregulation of Y box–binding protein 1 expression, suggesting that PGE_2_-EP1 signaling promotes hepatocarcinoma cells invasion (Zhang et al., 2014). These findings suggest that the EP1 receptor may be a therapeutic target to prevent and/or treat hepatocellular carcinoma.

## X. Bones, Joints, and Skeletal Muscle

### A. Osteoblastogenesis and Osteoclastogenesis

Bone mass results from the balance between osteoblast and osteoclast activities that are responsible for bone formation and resorption, respectively. These cells derive from different stem cells: mesenchymal for osteoblasts or hematopoietic for osteoclasts. In vitro studies concerning the control by PGs of these cell activities were mostly based on their capacity to modulate osteoblastogenesis or osteoclastogenesis (Blackwell et al., 2010; Lisowska et al., 2018a).

Cartilage and synovial fluids are other important components of connective tissues. The physiopathologic state in cartilage and synovium depends on the cellular activities of chondrocytes, synovial fibroblasts, and macrophages. PGs, PGE_2_ in most cases and PGI_2_ in some cases_,_ produced by these cells are surely involved in the onset and progression of chronic inflammation processes in cartilage tissues (Li et al., 2009; Nakata et al., 2018; Loef et al., 2019). These PGs are frequently responsible for an increase in IL-6, a proinflammatory cytokine for connective tissue (Hoxha, 2018). However, most of the current concept regarding the roles of PGs in bone-related diseases was based on the results in rodent models, and human relevance should be carefully confirmed.

The enzymes responsible for PGE_2_ and PGI_2_ synthesis (mPGES-1, PGIS) are present in bone and joint (Molloy et al., 2007; Nakalekha et al., 2010; Tuure et al., 2019). COX-1/2 and mPGES-1 enzymes have been shown to be expressed in human and rodent osteoblasts (Okiji et al., 1993; Murakami et al., 2000; Arikawa et al., 2004). The inflammatory stimuli, such as cytokines (IL-1*β*, TNF-*α*, TGF-*β*) and LPS, usually result in an increase in COX-2 and/or mPGES-1 expression in these osteoblast cells (Xu et al., 1997; Kobayashi et al., 2012; Pecchi et al., 2012). In consequence, PGE_2_ is preferentially synthesized among the PGs in osteoblasts (Xu et al., 1997). However, PGI_2_ is also abundantly found in synovial fluid in rheumatoid arthritis, and its main metabolite, 2,3-dinor-6 keto-PGF_1*α*_, was detected in urine of patients with rheumatoid arthritis (Hoxha, 2018).

#### 1. Effect of Nonsteroidal Anti-Inflammatory Drugs

In the in vivo situation, inhibition/deletion of COX-2 reduces pain; however, this enzyme has been suggested to be responsible for the loss of bone mass or mineral density both in humans and rodents (Robertson et al., 2006; Blackwell et al., 2010; Nakata et al., 2018). Many studies have shown that in humans and rodents treated with NSAIDs as well as in mice lacking COX-2 gene the bone-healing process after fracture is impaired (Pountos et al., 2012; Vuolteenaho et al., 2008). In a similar way, other anti-inflammatory drugs, such as glucocorticosteroids (e.g., dexamethasone), are responsible for impaired osteogenesis and could account for osteoporosis induction (Weinstein, 2012; Whittier and Saag, 2016). Treatment of human bone-marrow stromal cells (BMSCs) in vitro with dexamethasone induces the expression of the EP2 and EP4 receptors. In this case, osteoblast differentiation from human BMSCs is impaired, and PGE_2_ promotes adipogenesis instead of osteogenesis (Noack et al., 2015; Mirsaidi et al., 2017). Such a differentiation-controlling effect of PGE_2_ on BMSC differentiation was not observed in mouse cells; the adipocyte differentiation is inhibited by PGE_2_ (Inazumi et al., 2011; Fujimori et al., 2012). Interestingly, the beneficit of using NSAIDs to suppress the pathologic bone growth and heterotopic ossification has been shown in human and rat model (Zhang et al., 2013b; Lisowska et al., 2018b).

### B. Osteoblast

Among the PGE_2_ and PGI_2_ receptors, it was shown that the mRNAs for EP4 and IP are expressed in cultured osteoblasts derived from human trabecular bone (Sarrazin et al., 2001; Graham et al., 2009). Similarly, the presence of EP4 receptor has been described in rat osteoblasts (Nemoto et al., 1997) and in mice osteoblast-like cell line, MC3T3-E1 (Kasugai et al., 1995). In the latter report, the “EP2 receptor” detected by RT-PCR is in fact the subtype EP4 receptor.

In rodent BMSC or osteoblasts, PGE_2_ has been shown to exert pro-osteogenic effects and bone formation (Keila et al., 2001; Yoshida et al., 2002; Alander and Raisz, 2006; Ke et al., 2006; Weinreb et al., 2006; Mirsaidi et al., 2017). In agreement with the previous reports, the involvement of EP4 receptor in PGE_2_-induced bone formation was clearly demonstrated; a selective EP4-receptor agonist (ONO-4819) enhances the osteoblastogenic activity of bone morphogenetic proteins (BMPs) in mouse primary osteoblast (Nakagawa et al., 2007). Among the four PGE_2_ receptor–deficient mice, only EP4 knockout mice are unable to restore de novo bone formation via osteoblast stimulation (Narumiya and FitzGerald, 2001; Yoshida et al., 2002). Furthermore, knockout of the EP4 gene specifically in the sensory nerves inhibited bone formation because of PGE_2_ produced by osteoblastic cells in mice, suggesting a crosstalk in which sensory nerves sense bone density (Chen et al., 2019).

Pharmacological analysis of rat calvaria cells using the selective agonists (EP1: ONO-DI-004; EP2: ONO-AE1-259; EP3: ONO-AE-248; EP4: ONO-AE1-437) exhibited that EP2 and EP4 receptors mediate osteoblastogenic actions of PGE_2_ (Minamizaki et al., 2009); however, to date, no functional EP2 receptor has been identified on human osteoblasts or osteoclasts. For these reasons, an EP4-receptor agonist was selected for bone-targeting dual-action prodrugs: two classes of active agent, the EP4 agonist and a bone-resorption inhibitor (bisphosphonate), were coupled using metabolically labile linkers. Such a conjugate was efficient to reverse osteopenia in a rat model (Arns et al., 2012; Liu et al., 2015). Finally, there is no strong evidence showing the contribution of other EP subtypes (EP1 and EP3) in rodent bone formation.

### C. Osteoclast

In mouse and human, osteoclast precursors express EP2 and EP4 mRNA, but their expression levels were downregulated during differentiation into mature osteoclasts (Kobayashi et al., 2005; Take et al., 2005). In contrast, EP1 mRNA is expressed in the mouse mature osteoclast but not in the human osteoclast; there may be species difference in EP1 expression. Moreover, the difference in cell preparations may affect the expression of EP3 and EP4 receptors in human osteoclast; EP3/4 mRNA and proteins were detected in mature osteoclasts extracted from tibias and femurs of human fetuses (Sarrazin et al., 2004), whereas none of them were detectable in osteoclasts derived from human peripheral blood mononuclear cells treated with receptor activator of NF-*κ*B ligand and GM-CSF (Take et al., 2005).

It was reported that PGE_2_ promotes osteoclast differentiation from mouse bone marrow–derived macrophages through EP2 and/or EP4 receptor (Kobayashi et al., 2005), and this result may explain the impaired osteoclastogenesis detected in EP2 and EP4 knockout mice (Narumiya and FitzGerald, 2001). In addition, a pharmacological study using the selective EP-receptor agonists (EP1: ONO-DI-004; EP2: ONO-AE1-259; EP3: ONO-AE-248; EP4: ONO-AE1-329) in mouse bone-marrow cultures showed that activation of EP2 or EP4 receptor promotes the osteoclast formation. This study also revealed that bone resorption is mostly mediated by EP4 and partially by EP2 receptor (Suzawa et al., 2000).

In contrast to the PGE_2_ actions in mouse osteoclast formation, Takahashi and coworkers found that PGE_2_ inhibits osteoclastogenesis from human macrophages through the activation of EP2 and EP4 receptors (Take et al., 2005). This report excluded the involvement of EP3 and/or EP1 receptor(s) since 17-phenyl trinor PGE_2_ (EP1 agonist) and sulprostone (EP3 > EP1 agonist) failed to alter the differentiation. In both human and mouse cases, PGE_2_-EP2/EP4 pathway regulates osteoclastogenesis by modulating receptor activator of NF-*κ*B ligand signaling (Wani et al., 1999; Kobayashi et al., 2005; Take et al., 2005; Noack et al., 2015).

Compared with PGE_2_, the involvement of PGI_2_ in bone formation has been noted to a lesser extent in literature. However, IP receptor appears to be expressed in both human osteoblast and osteoclast (Fortier et al., 2001; Sarrazin et al., 2001). A selective IP-receptor agonist, ONO-1301, has been shown in rodent models to promote BMP-induced bone formation and, more specifically, the osteoblast differentiation in vitro and the ectopic and orthotopic bone formation in vivo (Kanayama et al., 2018).

### D. Arthritis

In rodent models of osteoarthritis and rheumatoid arthritis, in vivo administration of a selective IP-receptor antagonist or IP-receptor deficiency significantly reduced the symptoms observed in such chronic joint inflammation (Pulichino et al., 2006). This study indicates a detrimental role for PGI_2_ beside PGE_2_ in arthritis-like diseases, in which the concentrations of both PGs are increased in synovial fluids. These results are in accordance with another study using multiple mutant mouse strains and indicating deleterious roles of IP-, EP2-, and EP4-receptor signaling during collagen-induced arthritis (Honda et al., 2006). In contrast, the PG molecule mainly detected in human arthritis is PGE_2_, and there are few data showing the involvement of PGI_2_ (Sellam and Berenbaum, 2010; Brouwers et al., 2015) with the exception of two clinical studies (Gao et al., 2002; Mayerhoefer et al., 2007). These two studies show an analgesic effect of PGI_2_ analogs, which is in opposition to the rodents’ studies as discussed above (section *V. B. 3. Roles of Prostaglandin E*_*2*_ *Receptor 1, Prostaglandin E*_*2*_ *Receptor 2, Prostaglandin E*_*2*_ *Receptor 4, and Prostacyclin Receptor in Pain Perception*).

PGE_2_ is found in large amounts in the synovial fluid of patients with rheumatoid arthritis (Trang et al., 1977), and in mice a major role for the EP4 receptor has been shown as discussed already in Section *II. B. 2. Rheumatoid Arthritis*. For these reasons, novel approaches directed toward the EP4 receptor for human and animal use are being developed for arthritic pain and inflammation, including the EP4 antagonists CR6086 and Grapiprant (Nagahisa and Okumura, 2017; Caselli et al., 2018), the partial EP4 agonist GSK726701A (Healy et al., 2018), and the inhibitor of EP4-receptor internalization CP-25 (Jia et al., 2019; Han et al., 2020). Although these different therapeutic approaches appear on the surface to be paradoxical, an intriguing hypothesis is that they may all block PGE_2_-induced signaling resulting from an internalized EP4 receptor normally initiated by the PKA-dependent activation of G-protein–coupled receptor kinase 2 (GRK), which is interestingly the target for CP-25 (Jia et al., 2019). It is becoming increasingly evident that internalized receptor complexes (e.g., *β*-adreneric receptors), by being retained at various subcellular membrane compartments, can in general lead to a more sustained cellular response than signaling at the plasma membrane (Plouffe et al., 2020). Future studies should therefore be directed toward unraveling this with respect to the role of EP4 receptors in arthritis and other chronic inflammatory diseaes.

### E. Synovial Fibroblast

In rheumatoid arthritis or osteoarthritis, fibroblast and macrophages in synovial fluids are also responsible for PGE_2_ and PGI_2_ production (Mathieu et al., 2008; Peng et al., 2019). In human osteoarthritis synovial fibroblasts, mRNA for IP receptor and PGIS were detected. Indeed, stimulation of these cells with an endogenous proarthritis agent augmented PGI_2_ synthesis and mRNA levels of the IP receptor and MMP-13 (Molloy et al., 2007). Such an increase in MMP-13 expression was suppressed when fibroblasts were stimulated with an IP-receptor agonist, iloprost (Molloy et al., 2007). In contrast, PGE_2_ has been suggested to exert a proinflammatory action by stimulating triggering receptor expressed on myeloid cell-1 expression in monocytes via EP2/4 receptors (Peng et al., 2019). Since EP2 and EP4 mRNA were abundantly expressed in human synovial fibroblasts, PGE_2_ has been shown to stimulate IL-6 release from fibroblasts and to downregulate IFN-*γ–*induced anti-inflammatory actions, presumably via the EP2/EP4 receptors (Mathieu et al., 2008). An important role for EP4 receptor could be suggested since polymorphisms in *PTGER4* loci are associated with increased *PTGER4* gene expression in synovial biopsy samples from patients with spondyloarthritis, and *PTGER4* is a susceptibility gene for ankylosing spondylitis and RA (Evans et al., 2011; Rodriguez-Rodriguez et al., 2015).

PGI_2_-IP and PGE_2_-EP2/4 pathways appear to play a proinflammatory role in mouse synovial fibroblasts. In mouse model of collagen-induced arthritis, IL-6 release from synovial fibroblasts was significantly increased by the selective IP-receptor agonist, cicaprost and by either EP2 or EP4 agonist (Honda et al., 2006).

Globally, in every research field related to bone formation and cartilage degradation, it could be of importance to specify or confirm which receptors (mostly EP2 and/or EP4) are involved. It will be easier nowadays by using cell type–specific receptor knockout mice (conditional mice using Cre-Lox system) or the recent pharmacological tools, which allow us to discriminate more clearly between EP4- and EP2-receptor subtypes: EP4 (e.g., ONO-AE3-240; ONO-AE3-208) and EP2 (e.g., PF-04418948) antagonists or selective agonists for the EP2 (e.g., ONO-AE1-259) and EP4 (e.g., ONO-AE1-329, L-902,688) receptors. Few studies have used these specific agonists, and it is more obvious for the antagonists. The use of conditional mice or these pharmacological tools should increase the significance of conclusions.

### F. Chondrocyte

Cartilage degradation is the main deleterious effect in osteoarthritis and rheumatoid arthritis. It occurs when cartilaginous tissues are submitted to excessive mechanical loading. As a consequence, human or rodent chondrocytes produce proinflammatory cytokines (IL-6, IL-1*β*, TNF-*α*…) and PGE_2_. These mediators are significantly increased in synovial fluid of arthritic patients (Li et al., 2009; Lee et al., 2013; Brouwers et al., 2015; Sun et al., 2019). These molecules and, in particular, PGE_2_ are responsible for the increase in MMP expression and activity and thereby destruction of extracellular matrices, leading to sustained proinflammatory responses in rodents and human (Lee et al., 2013; Wang et al., 2013).

The EP1 receptor is unlikely to participate in such PGE_2_-elicited proinflammatory effects since the expression of subtype EP1 was hardly detected in human primary cultured chondrocytes, T/C-28a2 chondrocytes, and osteoarthritis cartilages (Aoyama et al., 2005; Wang et al., 2010; Sato et al., 2011). In contrast, EP2 receptor was reported to be expressed in various types of human chondrocytes (Li et al., 2009; Wang et al., 2010; Sato et al., 2011), and contribution of EP4 and EP3 receptors was to a lesser extent described. A detrimental role for PGE_2_-EP4 (or EP2)-receptor signaling is frequently concluded in the development of arthritis. PGE_2_ via EP2 receptor stimulates IL-6 production in human chondrocyte cell lines (T/C28a2) or primary articular chondrocytes subjected to high fluid shear stress (Wang et al., 2010; Sato et al., 2011). The activation of EP2 and EP4 receptors by either PGE_2_ or butaprost exerted antianabolic actions, resulting in decreased densities of collagen and aggrecan in human articular cartilage (Li et al., 2009). Similarly, in chondrocytes isolated from human knee cartilage, PGE_2_-EP4–receptor signaling has been identified to be responsible for the extracellular matrix degradation by increasing expression of MMP-13 and A disintegrin and metalloproteinase with thrombospondin motif 5 (Attur et al., 2008). Although the authors compared the suppressing potencies of EP2 and EP4 antagonists on the PGE_2_ actions, it might be difficult to discriminate EP4-mediated actions from EP2-mediated ones, since the antagonists (AH6809 and AH23848) they used in this study were the most “classic” compounds with lower binding affinities.

Aoyama et al. (2005) found in human cartilage that EP2-receptor mRNA was the most abundantly detected and that this tendency was also observed in mouse cartilage. The authors further showed that EP2 agonist promotes growth and cAMP content in human articular chondrocytes (Aoyama et al., 2005). Clark et al. (2009) demonstrated in mouse sternal chondrocytes that PGE_2_ attenuates chondrocyte maturation in a cAMP-dependent manner, presumably via EP2/EP4 receptor. The authors also found that PGE_2_ delays chondrocyte maturation at least partly by inhibiting BMP/Smad signaling in rat cell line (Clark et al., 2009). The same group also demonstrated in mouse primary costosternal chondrocytes that COX-2-PGE_2_-EP4–receptor pathway mediates BMP-2–induced phosphorylation of a transcription factor (activating transcription factor 4), a key transcription factor regulating bone formation (Li et al., 2014).

### G. Skeletal Muscle

Mouse myoblasts express all PGE_2_ receptors (i.e., EP1**–**4). Before skeletal muscle and myotube formations, myoblast proliferations were induced by PGE_2_ that was mediated by activation of the EP4 receptor since a similar result was found only with a selective EP4 agonist (CAY10598), and the effect of PGE_2_ was blocked by L161,982 (Mo et al., 2015). Similarly, in muscle-specific stem cells, the EP4 receptor is also involved in the expansion and regeneration of skeletal muscle in mice via cAMP/phospho-CREB pathway and activation of the transcription factor, Nurr1 (Ho et al., 2017).

In human, EP3 and EP4 receptors are expressed in skeletal muscle biopsies of the thigh (*vastus lateralis*), and mostly EP4 expression is associated to anti-inflammation profile in muscle and linked to exercise training (Lavin et al., 2020). These receptors are also described and are more expressed in human skeletal muscle of the leg containing mostly type-1 fibers (*soleus*) (Liu et al., 2016).

Finally, in humans or mice, PGE_2_ and EP4 agonists, as in many other cells, could have similar role in skeletal muscle in healthy and pathologic conditions. In myopathy, a greater level of PGE_2_ was found in skeletal muscle samples derived from humans and mice. The levels of PGE_2_ were strongly increased in muscular biopsies of patients with Duchenne dystrophy or myotonic dystrophy type 1 in comparison with control patient samples (Jackson et al., 1991; Beaulieu et al., 2012). This increase was associated with upregulation of COX-2, mPGES-1, and EP2/4 receptors, which could be involved in the pathogenesis since inhibition of PGE_2_ secretion by blocking COX (using aspirin) or mPGES1 (using a short hairpin RNA) in myoblasts reversed PGE_2_’s inhibitory effects on myogenic differentiation (Beaulieu et al., 2012). Similarly, in the animal model of myopathy (dystrophin-deficient mice), PGE_2_ production was also significantly increased after stimulation of leg skeletal muscle (*extensor digitorum longus*) with ionophore A23187 or electrical stimuli (McArdle et al., 1991).

## XI. Conclusion

The mechanisms whereby PGE_2_ and PGI_2_ exert their pleiotropic actions, once a mystery in physiology, have been clarified through the biochemical identification and cDNA cloning of the four EP subtype receptors and IP receptor. Furthermore, development of highly selective agonists and antagonists to each EP subtype and information obtained by studies on mice deficient in each EP receptor now provide opportunities to apply our knowledge to manipulate various PG-mediated pathologic processes (Table 3). In most cases, animal models and studies on human preparations give similar results with some exceptions. Although gene deletion of mPGES-1 has extensively been used experimentally to demonstrate an important role of PGE_2_ in regulating many types of inflammatory disease, the interpretation of studies is confounded by the biosynthesis of PGI_2_ being intrinsically linked to the downregulation mPGES-1. Although EP2 and EP3 receptors have important roles in human physiology and therapeutics globally, the number of recent publications during the last 10 years and the works presented in this review and in Table 3 show increasing interest for research involving the EP4 or the IP receptors. The activation of these two receptors is, or could be, an excellent therapeutic target in human by inducing vasodilatation, bronchodilation, skeletal, and bone-mass regulation by stimulating anti-inflammatory cytokine release from innate lymphoid cells or by inhibiting neointimal-, thrombosis-, and macrophage-associated inflammation. However, side effects of these activators are not excluded and could be related to pain induction. In addition, a number of drugs targeting/blocking the EP4 receptor are in clinical development and look promising against osteoarthritis-related pain, with one already approved for veterinary use. Finally, human genome-wide association studies are increasingly providing confirmation of experimental findings and insight into controversies or unearthing novel functions of the synthetic pathway of PGE_2_ and PGI_2_ and their associated receptors.

**TABLE 3:** Major similarities or differences between human and rodent EP1–4 or IP receptors Receptors less frequently reported are shown in parentheses.

System/Tissue	Point of Comparison	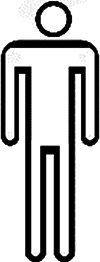 Receptor	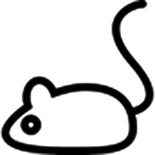 Receptor
Whole body	EP3 protein	5 isoforms	3/4 isoforms
Immune	Increased IL-23 production by DCs and macrophages	EP4/EP2	EP4/EP2
	PGE_2_ facilitation of Th17 cell function	EP2, EP4	EP2, EP4
	PGE_2_ inhibition of ILC2	EP4	EP4
	PGE_2_ stimulation of ILC3	unidentified	EP4
	PGE_2_ inhibition of M1 macrophage activity	EP4	EP4
	PGE_2_ stimulation of M2 macrophage activity	EP4	EP4
	PGE_2_ induced inhibition of eosinophils	EP2, EP4	EP2
			
Cardiovascular	PGI_2_ induced vasorelaxation (in most vessels)	IP	IP
	PGE_2_ induced vasoconstriction (in most vessels)	EP3	EP1 (EP3)
	PGE_2_ induced vasorelaxation in pulmonary vein	EP4	EP2 (EP4)
	PGE_2_ induced vasorelaxation in pulmonary artery	No relaxation	EP2 and/or EP4
	PGE_2_ induced vasorelaxation in renal artery	EP4	EP2 and EP4
	Vascular tone induced by PGI_2_ in diabetes	Increased relaxation	Increased contraction, decreased relaxation
	Increased expression of receptor in obesity	EP3	EP3
	PGI_2_ or analog-induced beneficial effect in stroke	IP	IP
	PGE_2_ increase of endothelial barrier	EP4	EP4
			
Thrombosis	PGI_2_ and PGE_2_ inhibition of platelet aggregation	IP (EP4 > EP2)	IP (EP4 > EP2)
			
Neuronal	Enhanced neuroinflammation in CNS (including AD, PD, and ALS)	unidentified	EP2 and/or EP4
	Enhanced neuroinflammation in CNS (MS)	EP2	EP2 and/or EP4
	Enhanced pain perception in PNS (depending on pain sensation)	unidentified	EP1, EP2, EP4 and IP
	Increased expression of receptor in AD	EP3	EP3
			
Respiratory	PGE_2_ induced airway smooth muscle relaxation	EP4	EP2 in mice, EP4 in rat
	Inhibition of allergic lung inflammation	EP2 and EP4	IP, EP4
	Antifibrotic effect	EP2 and IP (EP4)	EP2 and IP
			
Urinary	Increased expression in interstitial cystitis	EP1 and EP2 mRNA	EP1 and EP2 mRNA
	Receptor expression in ureters	EP1	EP1, EP2 and EP4
	Stimulation of renin secretion and renin gene expression in juxtaglomerular cells	EP4	EP4
			
Reproductive	Corpus cavernosum relaxation	EP2 and EP4	EP4
	Uterus contraction	EP3	EP3
			
Gastrointestinal	PGE_2_ inhibition of acute colitis	EP4	EP4
	Gastric-ulcer protection by EP agonist	EP3	EP3
	Colon contraction induced by PGE_2_	EP1	EP1, EP3
	Gastrointestinal muscle cells contraction	EP1	EP1
	Gastrointestinal muscle cells relaxation	EP4	EP4
			
Bone and joint	Osteoblastogenesis stimulated by PGE_2_	EP4	EP4 (EP2)
	Osteoclastogenesis regulated by PGE_2_	EP2 and EP4	EP2 and EP4
	IP activation by PGI_2_ on synovial fibroblast	Anti-inflammatory	Proinflammatory

## Abbreviations

AA: arachidonic acid
AAA: abdominal aortic aneurysm
AC: adenylate cyclase
AD: Alzheimer disease
Akt: protein kinase B
ALS: amyotrophic lateral sclerosis
Ang-II: angiotensin 2
APPS: Swedish amyloid precursor protein
A*β*: *β* amyloid
BMP: bone morphogenetic protein
BMSC: bone marrow stromal cell
CCL: chemokine ligand
CD: cluster of differentiation
CES1: carboxylesterase 1
CML: chronic myelogenous leukemia
CNS: central nervous system
COPD: chronic obstructive pulmonary disease
COX: cyclooxygenase
CRC: colorectal cancer
CREB: cAMP response element-binding protein
CSC: cigarette smoke condensate
CSF: cerebrospinal fluid
CXCL: C-X-C motif chemokine ligand
DC: dendritic cell
DRG: dorsal root ganglion
DSS: dextran sodium sulfate
EAE: experimental autoimmune encephalomyelitis
EP: prostaglandin E_2_ receptor
Epac: exchange protein directly activated by cAMP
ERK: extracellular receptor kinase
FP: prostaglandin F receptor
GM-CSF: granulocyte-macrophage colony-stimulating factor
HBEC: human bronchial epithelial cell
HD: Huntington disease
HEK: human endothelial kidney
HPC: hematopoietic progenitor cell
HSC: hematopoietic stem cell
IBD: inflammatory bowel disease
IFN: interferon
IL: interleukin
ILC: innate lymphoid cell
iNOS: inducible nitric oxide synthase
IP: prostacyclin receptor
IP_3_: inositol triphosphate
LPS: lipopolysaccharide
MCP-1: monocyte chemoattractant protein-1
MMP: matrix metalloproteinase
mPGES: microsomal PGES
MS: multiple sclerosis
NET: neutrophil extracellular trap
NF-*κ*B: nuclear factor *κ* light chain enhancer of activated B cells
NSAID: nonsteroidal anti-inflammatory drug
Nurr1: nuclear receptor related-1
OVA: ovalbumin
PAH: pulmonary arterial hypertension
PCR: polymerase chain reaction
PD: Parkinson disease
PDGF: platelet-derived growth factor
PG: prostaglandin
15-PGDH: 15-hydroxyprostaglandin dehydrogenase
PGE_2_: prostaglandin E_2_
PGES: prostaglandin E synthase
PGI_2_: prostacyclin
PGIS: PGI_2_ synthase
PH: pulmonary hypertension
PI3K: phosphatidylinositol 3-kinase
PK: protein kinase
PLC: phospholipase C
PNS: peripheral nervous system
PPAR: peroxisome proliferator–activated receptor
PS1: presenilin-1
RA: rheumatoid arthritis
RT-PCR: real-time PCR
SMC: smooth muscle cell
SNP: single-nucleotide polymorphism
TGF-*β*: transforming growth factor-*β*
Th1: type 1 helper T cell
Th17: type 17 helper T cell
Th2: type 2 helper T cell
TNF: tumor necrosis factor
TP: prostanoid TP receptor
Treg: regulatory T cell
TRPV-1: transient receptor potential vanilloid 1
TxA_2_: thromboxane A_2_
WT: wild type
